# Sub-Doppler rubidium atom cooling using a programmable agile integrated PZT-on-SiN resonator

**DOI:** 10.1038/s41467-026-77526-9

**Published:** 2026-09-16

**Authors:** Andrei Isichenko, Steven Carpenter, Nick Montifiore, Jiawei Wang, Mayand Dangi, Nitesh Chauhan, Pritha Mukherjee, Xuting Yang, Nitin Indukuri, Mark W. Harrington, Chuan Zhong, Iain M. Kierzewski, Ryan Q. Rudy, Jennifer T. Choy, Daniel J. Blumenthal

**Affiliations:** 1 Department of Electrical and Computer Engineering, University of California Santa Barbara, Santa Barbara, California USA; 2 Department of Physics, University of Wisconsin-Madison, Madison, Wisconsin USA; 3 Department of Electrical and Computer Engineering, University of Wisconsin-Madison, Madison, Wisconsin USA; 4 Department of Materials Science and Engineering, University of Wisconsin-Madison, Madison, Wisconsin USA; 5 U.S. Army Combat Capabilities Development Command Army Research Laboratory, Adelphi, Maryland USA

**Keywords:** Lasers, LEDs and light sources, Optical physics, Atom optics, Atomic and molecular physics

## Abstract

Programmability and precise control of laser frequency are essential for quantum experiments and applications such as atomic clocks, quantum computers, and cold-atom sensors. Current systems use bulky, power-hungry modulators and frequency shifters which are difficult to integrate and limit portability and scalability. We report an electrically controllable, agile optical frequency source based on a semiconductor laser stabilized to a photonic integrated resonator cavity with a lead zirconate titanate (PZT) actuator. We demonstrate this approach with precision programmable frequency control of a 780-nm laser that can periodically reference to rubidium-87 (^87^Rb) spectroscopy followed by fast, programmable, arbitrary frequency tuning sequences for quantum control. We use this approach to demonstrate sub-Doppler cooling of ^87^Rb without any external modulators, achieving atom-cloud temperatures as low as 16 *μ*K. The device achieves a tuning strength up to 1 GHz/V with 11 MHz modulation bandwidth while consuming only 10 nW of electrical power. This work establishes a route toward compact, low-power, and chip-scale laser systems for next-generation quantum and atomic sensing technologies.

## Abstract (web summary)

Frequency programmability often comes at the expense of setup complexity in cold-atoms experiments. Here, the authors advance lead zirconate-titanate (PZT) on silicon nitride (SiN) technology by demonstrating a stress-optic tunable ring resonator at 780nm to achieve sub-Doppler laser cooling of Rb atoms in a 3D magneto-optical trap, eliminating the need for external modulators.

## Introduction

Precision laser frequency control underpins a wide range of quantum technologies, including optical atomic clocks^1^, quantum computing^2^, and cold-atom inertial sensors^3,4^. These systems rely on narrow-linewidth lasers that must resolve and be stabilized with respect to atomic transitions. For example, rubidium-87 (^87^Rb) systems operating near 780-nm wavelengths require absolute frequency drift below the 100 kHz level for precision sensor performance^5–7^. In a cold-atom magneto-optical trap (MOT), this frequency is red-detuned by tens of megahertz for trapping and then rapidly returned to near resonance for fluorescence detection. In addition, achieving sub-Doppler temperatures requires dynamic, precisely timed detuning sequences: polarization-gradient cooling (PGC)^8^ and *Λ*-enhanced gray molasses^9^ depend on rapid, millisecond-speed frequency ramps of tens of megahertz, while Raman transitions used for coherent state manipulation often demand gigahertz-scale frequency jumps. The ability to control these frequency offsets with both long-term stability and short-term agility is therefore fundamental to reaching the ultra-low temperatures and coherence needed for high-precision sensing and quantum-state readout.

Techniques for controlling laser frequency have achieved a high degree of maturity, with ongoing efforts to miniaturize and scale. Long-term laser frequency stability is conventionally achieved by referencing to atomic vapor-cell spectroscopy, such as saturation-absorption spectroscopy (SAS), while fast, programmable detuning is realized through external modulation or direct laser control (e.g., by tuning laser current and temperature) in combination with offset beat-note locking^10^. Acousto-optic modulators (AOMs) can frequency shift by hundreds of megahertz, and electro-optic modulators (EOMs) apply sidebands that extend this range to multi-gigahertz offsets. These tools form the basis of many compact cold-atom instruments: single-seed laser architectures employing AOMs and EOMs have enabled field-deployable atom interferometers^11^ and recent photonic implementations at the C-band have demonstrated high-speed, single-sideband frequency shifting and modulation^4,12^. However, these approaches achieve frequency agility at the cost of system complexity and power consumption. In particular, bulk-optic and fiber-coupled AOMs require watt-level radio-frequency (RF) power amplifiers and lose optical power to unwanted diffracted orders, while EOMs generate parasitic sidebands that can degrade atom interferometer sensitivity^7,13^. Integrated solutions operating at C-band must also rely on frequency doubling to reach visible wavelengths, introducing conversion inefficiency and packaging overhead. Offset beat-note locking of the frequency-agile cooling laser is a reliable solution, but requires extra lasers for operation^13^. These limitations constrain the scalability of conventional architectures toward photonic integrated, compact, low-power, field-deployable quantum systems.

A critical next step is the development of visible and near-infrared (NIR) precision frequency control at atomic transition wavelengths, such as the 780-nm D_2_ line of ^87^Rb. Frequency modulation realized in the CMOS-compatible silicon nitride (Si_3_N_4_, hereafter referred to as SiN) waveguide platform is especially important for atomic applications due to its wide transparency window (405–2350 nm)^14^, ultra-low losses for laser frequency stabilization^15^, and compatibility with heterogeneous or hybrid integration with active materials such as GaAs, GaN, and InP. In particular, stress-optic modulation using lead zirconate titanate (PZT) on SiN is well suited to atomic frequency control as it enables wideband (DC-coupled to tens of MHz bandwidth) and low-power control. PZT integration on silicon nitride introduces negligible additional optical loss and operates with nanowatt-level power consumption^16–20^. PZT provides significantly stronger electromechanical coupling, enabling larger frequency shifts at lower drive voltages. To date, most PZT modulation is in the 1550-nm waveband, while recent work extending stress-optic modulation into the visible spectrum^21,22^ has been reported. However, agile and arbitrary frequency control using integrated stress-optic tuning in the visible-to-shortwave infrared (SWIR) range has not yet been demonstrated or applied in a cold-atom experiment.

In this work, we demonstrate a 780-nm PZT-on-SiN stress-optic ring resonator that functions as a frequency-agile programmable optical cavity for laser frequency control. We then apply it to achieve sub-Doppler cooling of ^87^Rb atoms without any external modulation. Programmed voltage waveforms applied to the PZT actuator enable deterministic and repeatable control of the Pound-Drever-Hall (PDH)-locked laser frequency. We measure a static tuning efficiency of 1 GHz V^−1^, 11-MHz small-signal modulation bandwidth, and sub-10 nW electrical power consumption, enabling precise DC tuning and agile modulation without degrading the optical Q factor or requiring additional optical power. Using this frequency-programmable cavity, we realize rapid optical frequency ramp sequences for PGC in a 3D MOT, achieving atomic temperatures as low as 16 μK—well below the 146 μK Doppler limit—using only the PZT resonator and a semiconductor optical amplifier (SOA), without an AOM in the loop for frequency shifting or intensity control. The PZT cavity provides holdover between periodic re-referencing to ^87^Rb spectroscopy, illustrating a compact, low-power route to laser frequency synthesis for cold-atom control. This approach is inherently compatible with self-injection locking, establishing a pathway toward fully integrated, frequency-programmable laser systems for quantum sensing and atom interferometry.

## Results

**Fig. 1:**
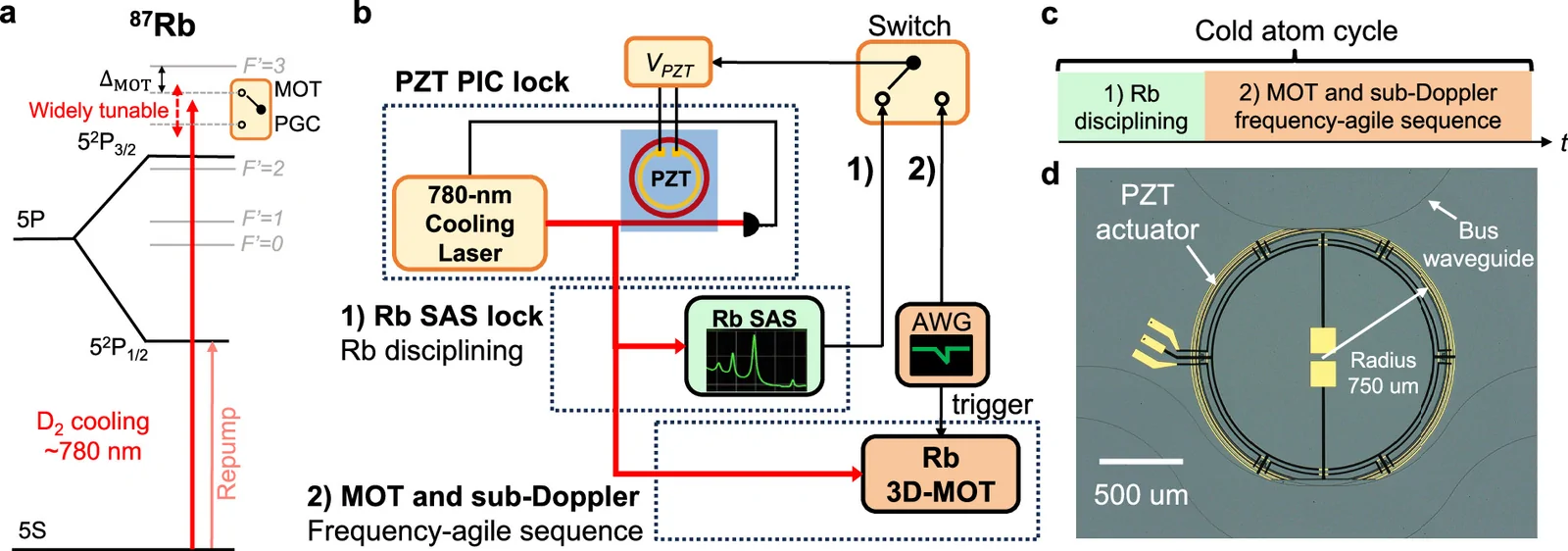
Working principle of rubidium-87 (^87^Rb) sub-Doppler cooling using an agile integrated PZT-controlled silicon nitride cavity. **a** Simplified ^87^Rb energy-level diagram showing the cooling and repump transitions and the rapid PZT-controlled detuning of the 780 nm cooling laser enabling switching between the magneto-optical trap (MOT) loading, spectroscopy referencing, and dynamic frequency control during the sub-Doppler cooling ramp. **b** A 780-nm distributed Bragg reflector (DBR) laser is Pound-Drever-Hall (PDH) locked to a photonic integrated circuit (PIC) resonator containing a lead zirconate titanate (PZT) stress-optic actuator. The laser closely tracks the resonator (PZT-to-PIC lock), and the laser drift is reduced at short timescales (i.e., drift holdover). The PZT is actuated such that the locked laser is stabilized to an ^87^Rb hyperfine transition using saturation absorption spectroscopy (SAS), which provides ^87^Rb disciplining and long-term frequency stability. During the cooling cycle, we switch off the ^87^Rb lock (while PZT PIC lock remains active) and apply a fast control signal to the PZT to rapidly shift the laser frequency for the ^87^Rb MOT and for sub-Doppler atom cooling. **c** The entire cold atom cycle consists of times when the laser and PZT PIC are ^87^Rb disciplined (1) and when the PZT PIC provides frequency agility and short-term holdover during the atom cooling experiment (2). **d** Micrograph of the PZT-actuated SiN ring resonator cavity, ring radius 750 μm.

In laser-cooled ^87^Rb systems, rapid frequency tuning of the cooling laser is required to transition from magneto-optical trap (MOT) loading to sub-Doppler cooling, as illustrated in Fig. 1a. The PZT-on-SiN resonator provides an integrated solution for this requisite locked laser frequency control, combining short-term stability with fast tuning for programmable control and long-term atomic referencing. The working principle of the PZT-controlled agile resonator cavity used to implement this functionality is shown in Fig. 1b–d. A commercial single-frequency 780-nm distributed Bragg reflector (DBR) semiconductor laser (Photodigm) is PDH-locked to the resonator (Fig. 1b) near the middle of the 20 GHz laser mode-hop-free region. This enables the locked laser to frequency-track the agile cavity while reducing the short-term laser frequency drift. During the laser stabilization step, we implement a dual-stage lock^5,6,23^ to an ^87^Rb-SAS module, where we feed the signal from the SAS back into the PZT tuner, anchoring the PIC-locked laser to an atomic transition and reducing the long-term laser frequency drift. Our laser system combines commercial off-the-shelf (COTS) components with a custom PIC. The COTS components include the cooling and repump DBR lasers, a fiber-coupled SOA for optical power modulation and shuttering, and the free-space ^87^Rb SAS setup. Combined with SOA-based power modulation, the PIC-based frequency control removes the need for external acousto-optic or electro-optic modulators (AOMs, EOMs) for sub-Doppler cooling.

To perform the detuning ramp sequences necessary for sub-Doppler atom cooling, the ^87^Rb-SAS lock is periodically disabled, and an arbitrary waveform generator (AWG) drives the PZT with a pre-programmed voltage signal. This produces laser frequency ramps and jumps used for MOT formation and PGC. The process is repeated, periodically re-referencing to the ^87^Rb-spectroscopy to prevent any significant long-term frequency drifts, while the PIC resonator serves as a holdover cavity to bound the frequency drift between cycles (Fig. 1b, c). The mm-scale PZT-on-SiN resonator device is shown in Fig. 1d.

### Resonator characterization

**Fig. 2:**
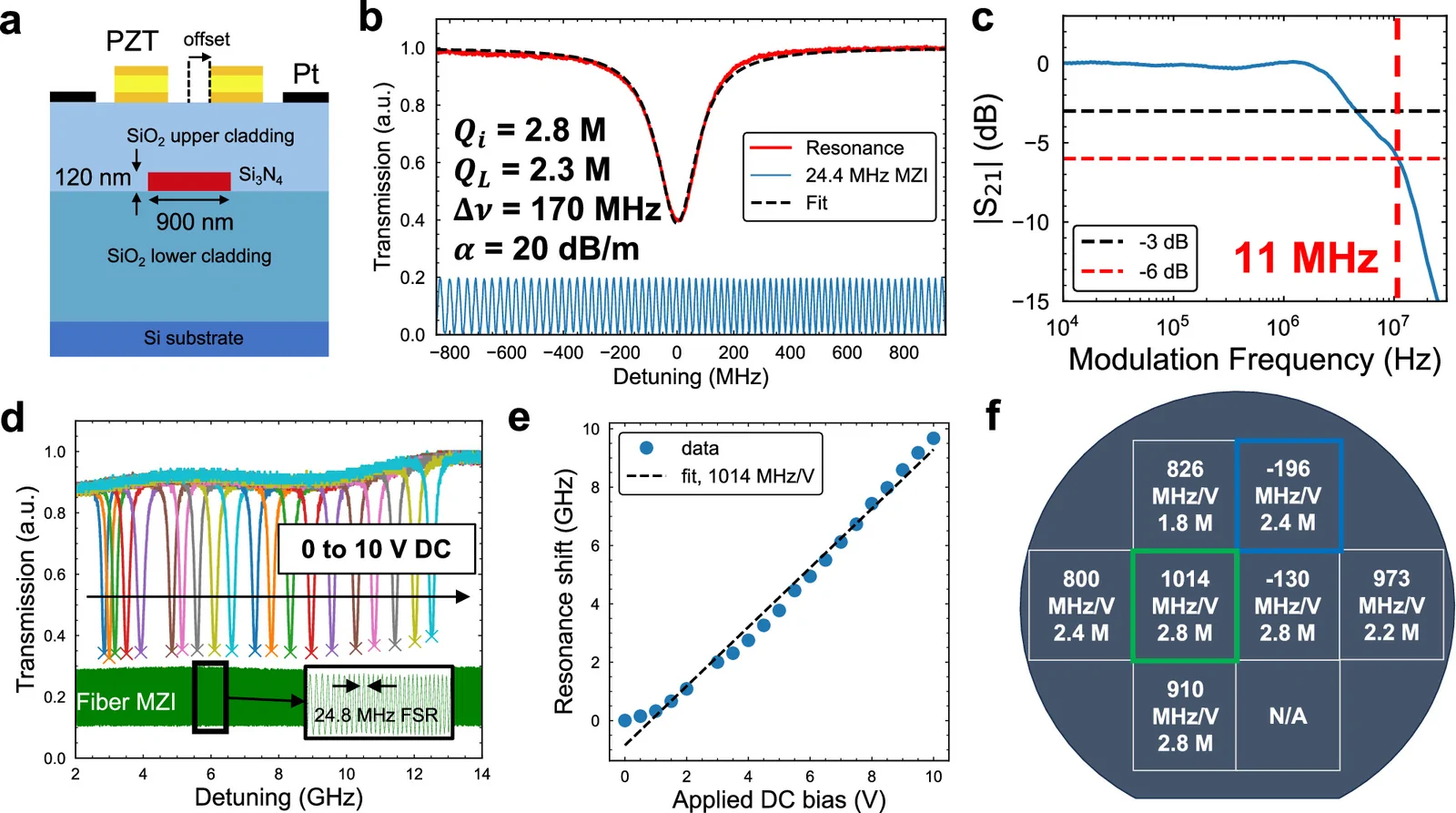
PZT PIC resonator design and characterization. **a** Cross-section of the silicon nitride ring resonator with PZT actuator and Pt thermal tuner, where the offset represents the lateral displacement between the PZT and the waveguide. **b** Transmission spectrum quality factor (Q) measurement of the ring resonator results in an intrinsic quality factor (*Q*_*i*_) of 2.8 million, a loaded quality factor (*Q*_*L*_) of 2.3 M, loss *α* = 20 dB m^−1^, and a total linewidth Δ*ν* of 170 MHz. The frequency detuning is calibrated using an unbalanced Mach-Zehnder interferometer (MZI, blue trace) due to laser current and frequency scan nonlinearity. **c** Frequency response of the PZT stress-optic small-signal modulation. The 6-dB modulation bandwidth is 11 MHz. **d** Transmission spectra for a single resonance as a function of applied bias voltage to the PZT. The frequency tuning is calibrated with an unbalanced fiber MZI (green trace). **e** The resonance frequency shift as a function of the applied DC bias. The measured tuning strength is 1 GHz V^−1^ ≈ 2 pm V^−1^. **f** Map of the 4-inch wafer DUV stepper lithography exposure reticles. Each reticle contains many devices; we report the data (PZT tuning strength, *Q*_*i*_) for only a single add-thru resonator device in the reticle. Green outline: device with the largest tuning strength, blue outline: device used for atom cooling demonstration, N/A: not applicable due to device damage. Variation in tuning strengths between dies is due to the designed PZT electrode offsets (which affect the stress field), variations in modal overlap between the waveguide and the stress field, and variations in PZT film quality and uniformity.

The cross-section design of the ring resonator is shown in Fig. 2a. The waveguide thickness is chosen for a moderately confined mode for a smaller bend radius, and the width is designed to support the TM_0_ mode. The resonator has a 750-μm radius, corresponding to a 38-GHz free-spectral range (FSR). The layer stack consists of a 15-μm SiO_2_ lower cladding, a 120-nm-thick and 900-nm-wide SiN core, and a 4-μm SiO_2_ upper cladding. The planar layer of PZT of 1-μm thickness is deposited on top of the waveguide with a lateral offset from the waveguide center. A pair of platinum electrodes is included for thermal tuning of the resonance, however, the thermal tuner is unused in this work. The PZT actuator deposition process does not require an undercut, unlike previous work^17,18^. The details of the fabrication process are in the Materials and Methods section.

The combination of the high quality factor (*Q*), fast modulation, and large-range PZT tuning is central to the device performance. For the TM-mode resonance, we measure an intrinsic *Q*_*i*_ = 2.8 M, loaded *Q*_*L*_ = 2.3 M, and a propagation loss of 20 dB m^−1^ (Fig. 2b). We measure the small-signal electrical-to-optical modulation response (*S*_21_) by tuning the laser onto the quadrature point of the resonance and using a network analyzer. We find the 6-dB cutoff frequency to be 11 MHz (Fig. 2c), and the 180^∘^ phase-lag point is 5.8 MHz (Supplementary Fig. 1a). The PZT actuator enables low-power frequency tuning over a broad range. The optical transmission spectrum (Fig. 2d) shows resonance tuning as a function of a 0- to 10-V DC control voltage with a 1 GHz V^−1^ linear tuning strength (Fig. 2e). Due to hysteresis in the stress-optic tuning, forward and reverse voltage sweeps yield slightly different resonance shifts, as shown in Supplementary Fig. 1b. The increased nonlinearity at low voltages is discussed further in Supplementary Note 1. We measure < 1 nA leakage current at 10 V applied voltage, indicating a < 10 nW power consumption. To evaluate wafer-scale uniformity, we measure the static tuning and intrinsic *Q*_*i*_ for devices across a 4" wafer (Fig. 2f). All seven measured devices exhibit > 100 MHz V^−1^ tuning strengths with the tuning polarity determined by the lateral offset of the PZT actuator relative to the waveguide, where positive (negative) tuning corresponds to no (2 μm) offset. The device shown in the green-outlined reticle achieved the highest *Q*_*L*_ and tuning strength. The device shown in the blue-outlined reticle was packaged (Supplementary Fig. 2) and used for the atom cooling demonstration.

### Sub-Doppler atom cooling

We next demonstrate the application of the PZT-on-SiN resonator as an agile frequency tuner for a PDH-locked laser used in a ^87^Rb cold-atom ensemble. This enables cooling of atoms in a three-dimensional MOT to temperatures well below the 146-μK Doppler limit through PGC^8^. Optimal PGC requires the MOT cooling light to be rapidly red-detuned, typically by 30–100 MHz within several milliseconds, while simultaneously reducing the optical power^4^. This dynamic detuning decreases the scattering rate and allows atoms to localize in the spatially varying light field, achieving sub-Doppler temperatures.

In this work, we focus on using the integrated resonator for frequency control. Conventionally, sub-Doppler cooling sequences rely on an AOM for two functions: (1) shifting the laser frequency to the desired detuning for MOT formation, and (2) applying a chirped RF drive to ramp up the detuning during the sub-Doppler stage. AOMs can also act as optical shutters, although this role can alternatively be fulfilled by an SOA or eventually by an extension of the same PZT mechanism presented in this work.

Using the agile frequency control of our PZT optical resonator, we replace the function of the AOM for both MOT formation and sub-Doppler cooling. In fact, no AOM is present in the path of the cooling beam. We control the system via a Vescent D2-125-PL servo controller, which enables rapid switching between spectroscopy-referenced operation and free-running, resonator-stabilized operation. In the free-running mode, arbitrary voltage waveforms applied to the PZT allow rapid frequency control of the cooling laser.

**Fig. 3:**
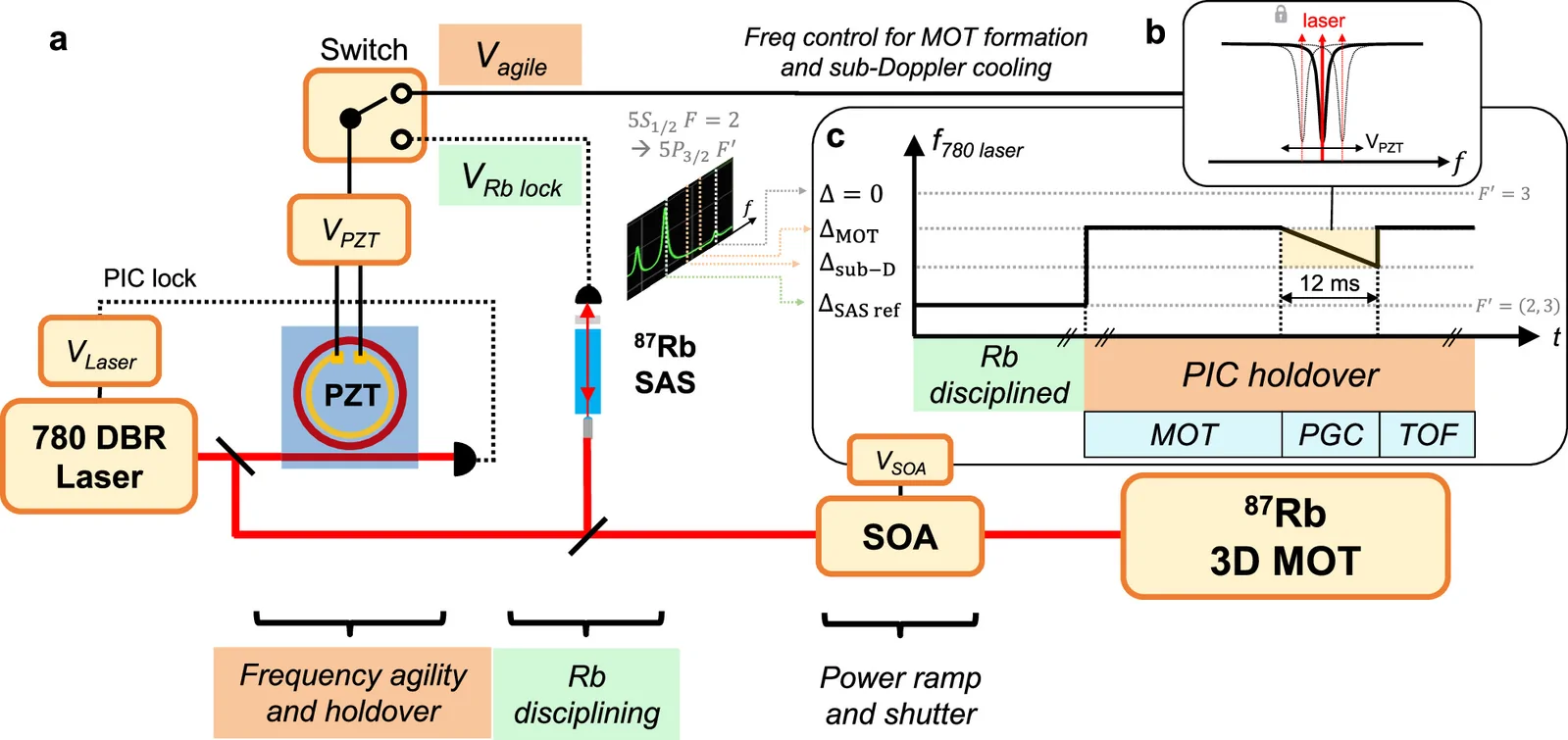
Cold atom experiment diagram. **a** The 780-nm DBR cooling laser is stabilized to the PZT PIC resonator cavity for reduced laser drift (i.e., holdover) and locked laser frequency control applied with *V*_PZT_. The PZT control switches between fast frequency control (*V*_agile_) used to control the cavity for 1) ^87^Rb disciplining, by locking to the ^87^Rb saturation absorption spectroscopy (SAS) and 2) MOT formation and for sub-Doppler cooling. The cooling laser light is amplified with a semiconductor optical amplifier (SOA), which is voltage-controlled (*V*_SOA_) for power ramping and shuttering, used for sub-Doppler cooling temperature measurements. A full description of optical train power losses is provided in Supplementary Note 1. **b** The *V*_agile_ frequency control tunes the cooling laser frequency, enabling arbitrary laser frequency control within the tuning range of the PZT actuator and at speeds within the locking range of the laser lock. **c** Timing diagram for one cold atom experiment cycle consisting of the ^87^Rb disciplining of the PZT PIC cavity and tuning the PIC to set the cooling laser frequency with respect to ^87^Rb hyperfine transitions. The ^87^Rb disciplining is done at the strong ${F}^{{\prime} }=(2,3)$ crossover transition for 25 ms (see Supplementary Fig. 7). The PZT is jumped by a set voltage to set the cooling laser at Δ_MOT_ detuning from the cooling (${F}^{{\prime} }=3$) transition. After full MOT loading, the fast frequency control signal is applied to the PZT to rapidly ramp the laser by 15 MHz over 12 ms for polarization gradient cooling (PGC) to achieve sub-Doppler cooling, measured during the time-of-flight (TOF) stage.

The schematic used to demonstrate and measure the locked-laser tuning is shown in Fig. 3a. We PDH-stabilize the 780-nm cooling DBR laser to the ring resonator and achieve locked laser frequency control with *V*_PZT_. The applied PZT signal alternates between the Rb lock control (*V*_Rb lock_) for ^87^Rb-disciplining and the fast frequency control (*V*_agile_) for MOT formation and for sub-Doppler cooling. The *V*_agile_ signal tunes the cooling laser frequency, enabling arbitrary laser frequency control within the tuning range of the PZT actuator and at speeds within the locking bandwidth of the PDH laser lock, as shown in Fig. 3b. A detailed schematic of the lock is shown in Supplementary Fig. 3, while measurements of the laser frequency noise, Allan deviation, and frequency-step response are provided in Supplementary Fig. 4 and discussed in Supplementary Note 1.

We amplify the cooling light via a fiber-coupled SOA, whose output power is voltage-controlled (*V*_SOA_) for programmable power ramping and shuttering. The SOA serves as a power modulator for PGC and as an optical shutter during time-of-flight (TOF) temperature measurements. The ^87^Rb MOT employs a six-beam (three beams with retro-reflection) configuration. The 780-nm laser drives a red-detuned ${}^{87}{{{\rm{Rb}}}}\,5{S}_{1/2}\,F=2\to 5{P}_{3/2}\,{F}^{{\prime} }=3$ transition, while a separate 795-nm DBR laser acts as the repump, addressing the ${}^{87}{{{\rm{Rb}}}}\,5{S}_{1/2}\,F=1\to 5{P}_{1/2}\,{F}^{{\prime} }=2$ transition (see Supplementary Fig. 5).

The timing diagram for one experimental cycle is shown in Fig. 3c. Each sequence begins with ^87^Rb disciplining at the ${F}^{{\prime} }=(2,3)$ crossover transition for 25 ms. While F’ = 3 may be a more natural locking point since it matches the cooling transition, the F’ = (2, 3) crossover feature is used instead as it has a six-fold better signal-to-noise ratio (SNR) in the absorption. This allows robust and consistent laser relocking during ^87^Rb re-referencing. The PZT voltage is then stepped by ~1 V to set the cooling laser at the MOT detuning Δ_MOT_ relative to the ${F}^{{\prime} }=3$ cooling transition, and the MOT is loaded for 223 ms. Following MOT loading, the *V*_agile_ waveform linearly ramps the laser detuning by an additional 15 MHz over 12 ms to implement polarization-gradient cooling (PGC). After this ramp, all MOT and repump light is switched off at *t* = 250 ms, initiating a TOF measurement with delays ranging from 0 to 6 ms. A 0.5 ms illumination pulse, applied at Δ_MOT_ (and optionally at Δ = 0 in future runs), captures the atomic fluorescence image. The illumination pulse is generated by the cooling laser, which is shuttered from the atoms via an SOA during the time of flight (TOF). During this period, the laser is set to the MOT formation (red-detuned) frequency and subsequently flashed onto the atom cloud via SOA intensity control. At *t* = 290 ms, the system re-engages ^87^Rb referencing by first returning the PZT to the lock point and then re-enabling the SAS feedback. The 290-ms total interval between ^87^Rb-spectroscopy re-referencing events was chosen to optimize fluorescence measurement SNR while keeping sufficiently fast (Hz-level) repetition rates needed for applications such as atom interferometry. Additional total interval times, including 100 and 500 ms, were tested without noticeable effects on single-cycle laser frequency drifts.

**Fig. 4:**
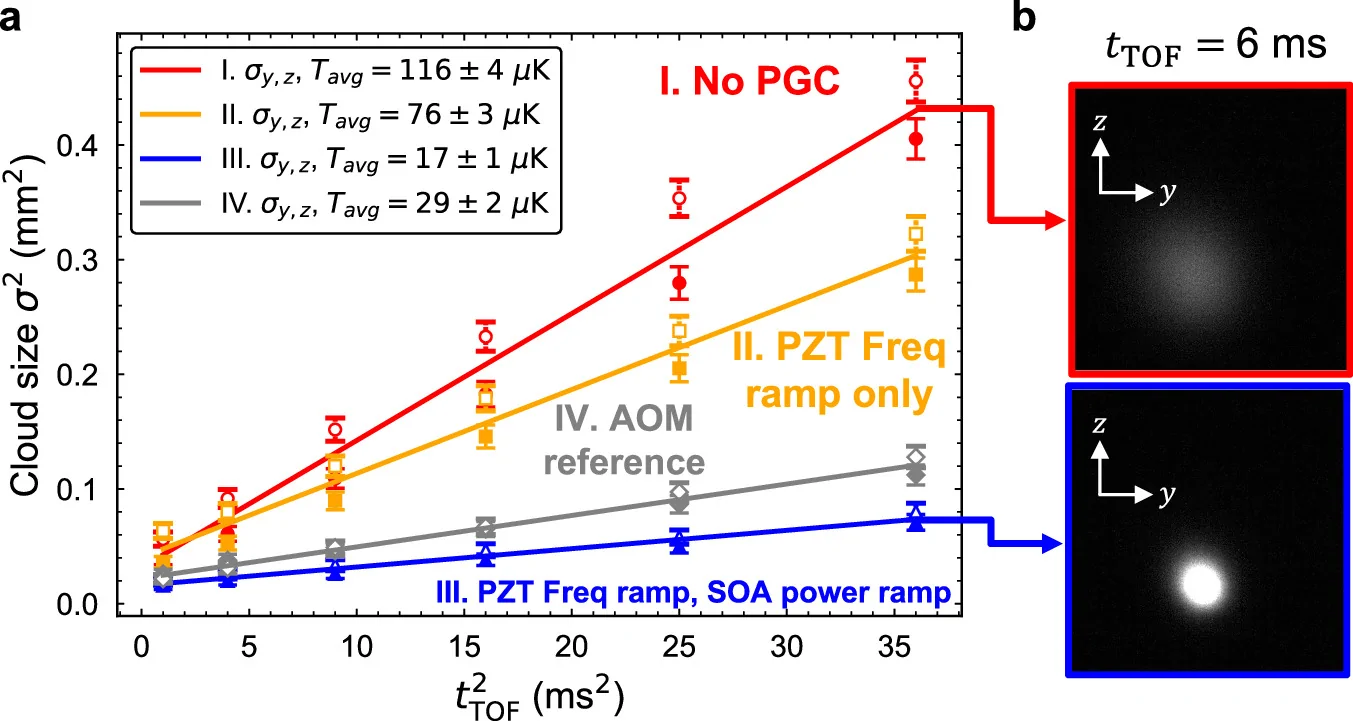
MOT temperature measurements for different cooling laser control configurations. **a** Time-of-flight (TOF) atom cloud temperature measurements, with the squared cloud radius, *σ*^2^, along the *z* (open markers) and *y* (filled markers) dimensions for different squared TOF times ${t}_{{{{\rm{TOF}}}}}^{2}$. The linear fits are for ${\sigma }_{i}^{2}={\sigma }_{i,0}^{2}+\frac{{k}_{B}{T}_{i}}{m}{t}_{{{{\rm{TOF}}}}}^{2}$, where *σ*_*i*_ is the Gaussian standard deviation of the cloud along an axis (*y*, *z*), *σ*_*i*,0_ is the initial width of the cloud, *k*_*B*_ is the Boltzmann constant, *m* is the mass of a single ^87^Rb atom, and *T*_*i*_ is the temperature along an axis. The temperatures reported in the legend are an average of both axial temperatures (which slightly differ due to asymmetries in beam alignment and in the magnetic field gradient in each axis). The schematic for the experiment configurations (I–IV) is shown in Supplementary Fig. 6. In each case, identical parameters were used for sequence timing, cooling, and repump frequencies and powers, and fluorescence detection. Each datapoint is extracted from a 10-shot accumulated fluorescence image (each with a 0.5 ms exposure). Vertical error bars represent the propagated Gaussian-fit standard deviation in *σ*^2^, including a one-pixel error floor. The quoted temperature uncertainties include both the fit/extraction uncertainty and the systematic uncertainty from the imaging calibration added in quadrature. **b** Fluorescence images of the MOT cloud after free-expansion for *t*_TOF_ = 6 ms. The color scale is identical for both images.

Atomic cloud temperatures are determined from the TOF measurements^24^ (see Supplementary Note 2). Squared TOF time values are plotted to linearize the extraction of atom cloud temperature (see Supplementary Equation S5 and Supplementary Table 2). Four test configurations are reported in Fig. 4a. Three of the test configurations employ the dual-stage laser lock. These tests, and their resulting temperatures with standard deviation uncertainty are as follows: (I) neither frequency nor laser intensity PGC ramps (no intentional sub-Doppler cooling) for which we measured 116 ± 4 μK, (II) only a frequency PGC ramp (via PZT) for which we measured 76 ± 3 μK, and (III) both a frequency (via PZT) and laser intensity (via SOA) ramp for which we measured 17 ± 1 μK. The fourth (IV) test configuration uses an AOM for a frequency ramp and the SOA for an intensity ramp, for which we measured 29 ± 2 μK. Supplementary Fig. 6 illustrates the four cooling configurations, and Supplementary Fig. 7 provides details of the experimental timing for configuration III. More discussion on the temperature data collection can be found in Supplementary Note 2.

The coldest measured temperature reaches 16 μK (in the y-axis) via configuration (III), with a comparison shown in Fig. 4b. In principle, the AOM-based configuration (IV) should enable similarly low temperatures; however, optimization is more challenging because the cooling laser power becomes detuning-dependent. As a result, the minimum temperature achieved using the AOM configuration was 28 μK.

### Resonator agility measurements

**Fig. 5:**
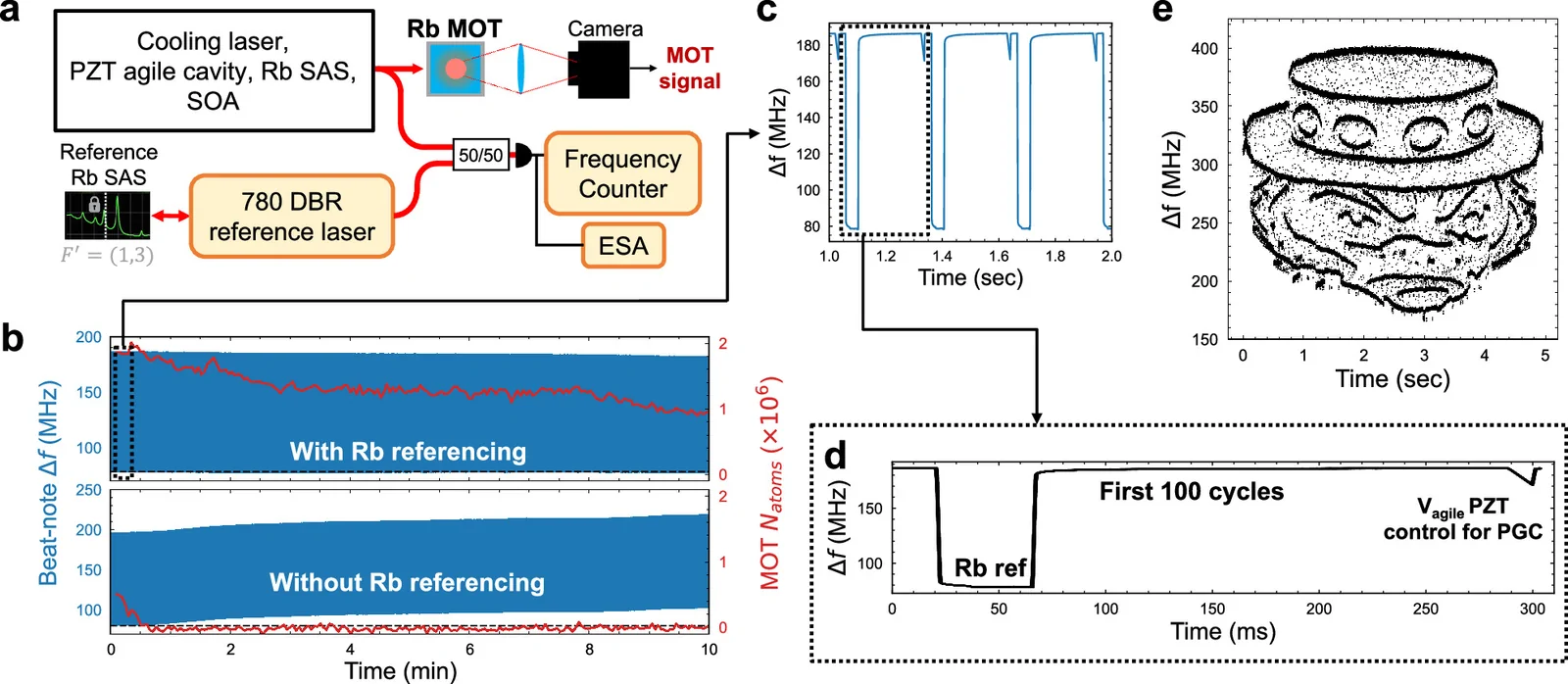
Cooling laser frequency agility measurements. **a** Schematic for monitoring the PZT-controlled cooling laser frequency during the MOT and sub-Doppler cooling. A reference 780-nm DBR laser is locked to another ^87^Rb spectroscopy system, and the heterodyne beat-note between the reference and the cooling lasers is recorded on a photodiode and a frequency counter. During the sequence, the beat-note is monitored on an electronic spectrum analyzer (ESA), and the MOT fluorescence signal is extracted from a camera. **b** Laser beat-note recorded on a frequency counter during 20 min of continuous operation. The reference laser is continuously locked to the ^87^Rb F = 1 → F' = (1,3) crossover transition. When the PZT cavity is re-referenced to the ^87^Rb SAS (with ^87^Rb reference, top), the beat-note remains near 78 MHz for the referencing (MOT formation) parts of the cycle. We record the MOT atom number the entire time. Note that atom number uncertainty (see Supplementary Note 2: Atom number calculation and Supplementary Table 1) is dominated by systematic error in imaging calibration and is ~20%. This constant error bar is neglected from the plot for clarity. Without the ^87^Rb referencing (bottom), the drift results in the loss of trapped atoms within the first minute of operation. Supplementary Fig. 8a demonstrates a strong correlation between cooling laser frequency drift and trapped atom number changes, suggesting that laser frequency drift is a primary factor in the observed variation in atom fluorescence. **c** Zoom-in of the beat-note in (b, top) for six cycles. **d** One period of **c** overlaid with 100 consecutive cycles, showing the stability of the PZT response during this period. The first jump has a 25 ms ^87^Rb referencing time, and the triangular ramp at 290 ms is the PGC ramp. Laser drift, which would smear the overlaid traces in this figure, only becomes prominent at timescales greater than one minute. **e** Measured laser beat-note trajectory generated by an arbitrary programmed waveform applied to the PZT actuator, demonstrating arbitrary, repeatable frequency synthesis.

We characterize the frequency-agility of the PDH-locked PZT-SiN resonator by monitoring how the laser frequency tracks cavity tuning within the PDH feedback bandwidth. Figure 5a shows the measurement schematic. A 780-nm DBR cooling laser, PDH-locked to the PZT resonator, is heterodyned with a second 780-nm reference DBR laser stabilized to an independent ^87^Rb D_2_-line SAS system. Using a fast photodiode, an electronic spectrum analyzer (ESA), and a frequency counter, we record the beat note between the two lasers. Simultaneously, a camera monitors the fluorescence from the ^87^Rb MOT.

Figure 5b shows the laser beat-note recorded over 20 minutes of continuous operation. While we periodically referenced the PZT cavity to the ^87^Rb SAS (top), the beat-note remained stable near 78 MHz during the ^87^Rb lock. However, a slow drift in resonator response to a fixed PZT drive voltage leads to a gradual shift in cooling-light detuning during extended operation, particularly during the first hour after system startup. Quantitatively, Fig. 5b shows a 0.7 MHz per minute (predominantly upward) drift in cooling detuning, Δ_MOT_. This behavior is attributed to long-time-scale effects in the PZT actuator. These effects, as well as hysteresis and creep^25^, are discussed further in Supplementary Note 1. Importantly, the periodic re-referencing to the ^87^Rb SAS maintains repeatable frequency states and prevents runaway drift. In future operation, we aim to further suppress the residual fluorescence drift by adding a slow trim loop using MOT fluorescence as an error signal^26^.

Without ^87^Rb referencing (Fig. 5b, bottom), frequency drift accumulates rapidly, leading to the complete loss of the MOT signal within the first minute of operation. This confirms that the hybrid PDH with the SAS scheme provides both short-term stability from the cavity and long-term drift suppression from the atomic reference. The re-referencing period can be selected according to the short-term frequency-stability requirements of the experiment. We have demonstrated re-referencing rates from 30 mHz to 100 Hz, including stable relocking after a 30 second MOT formation period in which the spectroscopy reference was applied only once per cycle. This further demonstrates the long-term drift suppression provided by the cavity.

Because the resonance shift is a stress-optic mechanical effect and the PDH laser-to-PIC lock must follow that shift (set by the PDH locking range and strength), the switching time depends on the frequency difference between starting and target frequencies. In a benchmark test, a 100 MHz frequency jump takes 300 μs, giving an effective slew rate of 0.33 MHz μs^−1^ (Supplementary Fig. 4c).

The zoomed-in section of three sequential cycles, shown in Fig. 5c, highlights the repeatable frequency trajectory of each cycle. Figure 5d overlays 100 consecutive cycles, showing consistent operation over the entire PZT drive range. Each sequence begins with a 25-ms ^87^Rb referencing interval followed by the MOT stage and concludes with a 12-ms triangular frequency ramp corresponding to the polarization-gradient cooling (PGC) sequence. The 25-ms ^87^Rb referencing interval used for this data collection was an overly generous choice; it was selected to ensure re-locking stability. However, this re-referencing time has since been reduced to a 4-ms interval without additional control complexity.

Finally, Fig. 5e illustrates the flexibility of the system: an arbitrary waveform applied to the PZT produces a programmed laser-frequency trajectory. This highlights the ability of the integrated PZT-SiN resonator to provide deterministic, high-speed, and repeatable frequency control over hundreds of MHz. Together, these results confirm that the resonator enables robust, long-term frequency holdover when referenced to ^87^Rb spectroscopy and precise, agile tuning on millisecond timescales—sufficient for implementing sub-Doppler cooling and dynamic frequency control in cold-atom experiments.

## Discussion

We have demonstrated an integrated 780-nm PZT-on-SiN resonator that enables programmable, precise, and agile laser frequency control for cold-atom applications. By locking a 780-nm DBR laser to the programmable high-Q (2.8 million intrinsic) resonator and driving the PZT actuator with sub-Doppler cooling voltage sequences, we cool ^87^Rb atoms to 16 μK in a 3D MOT using rapid 15-MHz linear detuning over 12 ms for PGC. In this demonstration, the PZT-based agile cavity fully replaces the role of the AOM in the sub-Doppler-cooled 3D MOT. This approach maintains stable laser lock and achieves atomic temperatures far below the 146-μK Doppler limit. These results demonstrate that wideband stress-optic tuning can provide both long-term stability and fast, programmable frequency agility within a single integrated photonic component. This approach reduces power consumption and mitigates issues with conventional RF-based approaches, such as AOMs or EOMs that generate unwanted optical sidebands and can parasitically excite nearby atomic transitions. By programming the optical reference resonator itself, the laser remains single-frequency and inherits a deterministic frequency trajectory from the high-Q resonator. Furthermore, this scheme is compatible with frequency noise reduction and linewidth narrowing due to the low thermo-refractive noise (TRN), high Q, and high extinction ratio (ER) of the resonator^23,27^. This sideband-free frequency synthesis eliminates spurious optical tones during the cooling sequence, simplifying system architecture and reducing undesired atomic excitations.

The SiN waveguide layer here matches the layer used in photonic integrated beam-delivery systems for magneto-optical traps^28^, underscoring the compatibility of this approach with large-area emitter arrays and integrated cold-atom platforms. Beyond frequency tuning, the same PZT actuation mechanism can be engineered for direct amplitude modulation and optical shuttering, either through interferometric or resonant configurations. Previous demonstrations of multi-ring and Mach-Zehnder interferometer (MZI) modulators in SiN and AlN platforms^29,30^ indicate that similar stress-optic actuation could enable fast amplitude modulation and switching. In parallel, SOAs have recently been demonstrated as integrated amplitude modulators and shutters^31^, providing a complementary path toward all-on-chip control of laser power and timing.

Once disciplined to the atomic reference, the resonator maintains frequency stability across the millisecond-scale cooling window without re-referencing, enabling reproducible 100-MHz frequency ramps over hundreds of cycles. This highlights the cavity’s intrinsic ability to serve as a short-term holdover cavity, which itself arises from the excellent short-term coherence provided by the high-Q SiN cavity and its low-power PZT actuation. This functionality eliminates the need for bulk optic reference cavities and further enables integration, compactness, and low-power operation. Experiments requiring multiple GHz-spaced optical frequencies used asynchronously throughout an experimental sequence, typically generated using multiple spectroscopy-referenced lasers and combinations of AOMs and EOMs, may instead be enabled by a single rapidly tunable integrated frequency reference. In addition, resonator-based tuning may be advantageous in applications where parasitic optical frequency components are undesirable, such as Raman-based atomic control schemes. However, conventional AOM and EOM architectures may still remain advantageous for applications requiring very large frequency shifts, broadband operation, or higher modulation bandwidths.

Looking forward, this stress-optic tuning approach is compatible with high-bandwidth self-injection-locked (SIL) architectures^27,32^, where direct feedback between the PZT-tuned resonator and the laser can further enhance frequency stability and tuning range. In the present work, however, we employ a fiber-coupled configuration, as achieving robust SIL is currently limited by modal mismatch between the edge-emitting laser and the integrated waveguide, which reduces feedback strength and locking stability.

The PZT-actuated resonator thus represents one element of a broader set of integrated photonic components required for atomic and quantum systems. When combined with narrow-linewidth external-cavity lasers at 852 nm^33^, self-injection-locked lasers at 780 nm^27^, and integrated vapor-cell spectroscopy references, this platform can enable fully photonic implementations of cold-atom sensors and clocks. Moreover, we may perform the ^87^Rb referencing using either a vapor-cell SAS or the MOT itself, as in self-locked MOT architectures^34^, offering a compact route to autonomous frequency stabilization.

**Fig. 6:**
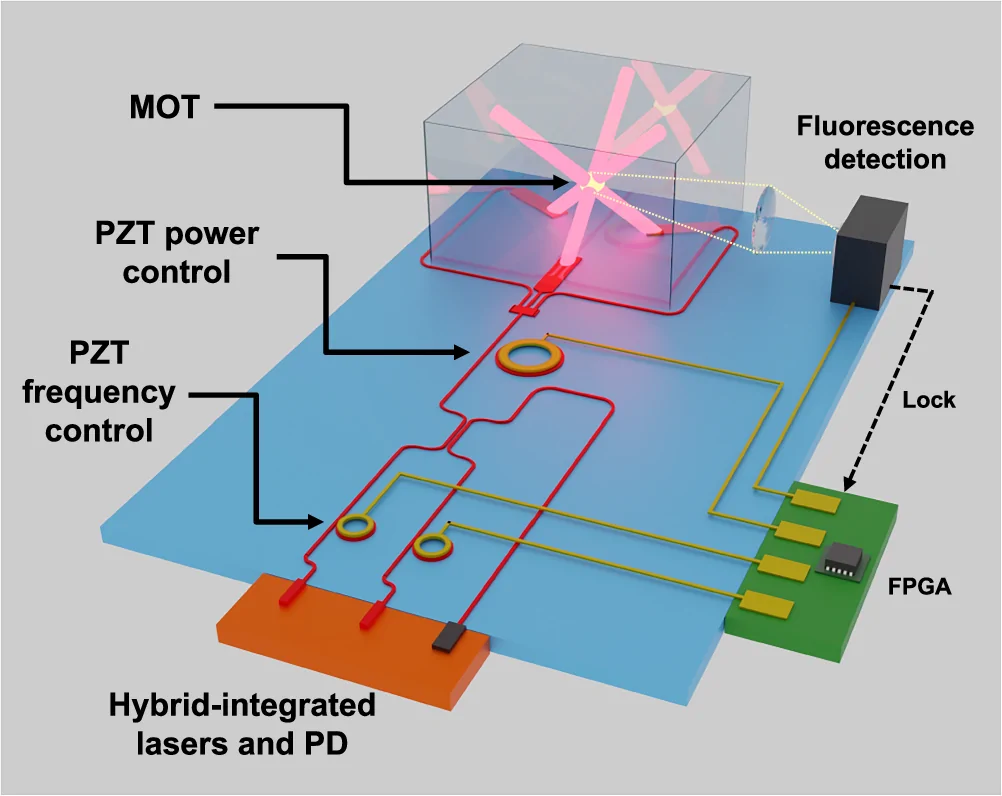
Example integrated cold atom system enabled by the 780 PZT resonators. A pair of self-injection-locked lasers consisting of hybrid-integrated edge-emitting lasers and PZT-on-SiN resonators form the frequency-agile cooling and repump lasers for a MOT. The retro-reflector mirrors and waveplates are omitted from the 3D-MOT beam delivery portion for clarity. The frequency spacing between the lasers is controlled with an offset beat-note lock realized with a directional coupler splitter and a photodetector. The power shuttering and ramping are controlled with a critically-coupled PZT resonator. The absolute frequency of the cooling laser is set based on feedback from the MOT fluorescence.

Figure 6 presents our vision for a fully integrated cold-atom control system enabled by the fast-tunable PZT-SiN resonators we demonstrate here. In this architecture, a pair of self-injection-locked 780-nm lasers provides the cooling and repump light. Independent PZT actuators stabilize and tune each laser. The frequency spacing between the lasers is set by an on-chip offset beat-note lock using an integrated directional coupler and photodetector. We aim to implement power ramping and shuttering through critically coupled resonators driven by PZT amplitude control, while fluorescence feedback from the MOT provides frequency referencing. We can achieve > 10 dB extinction ratio^19,22^ via shifting this resonance onto and off of the laser frequency. Recent demonstrations of PZT-actuated photonic devices have achieved optical shuttering with a 1.7 μs (90–10%) switching time and 21.5 dB extinction ratio^22^. Square wave shutoff or programmable power sequences may be implemented via mapping the resonance transmission transfer function in the control hardware. Additionally, we are developing an FPGA-based controller for complex waveform generation, data processing, and feedback control to increase frequency precision and agility^35–37^. This system concept represents a path toward a chip-scale cold-atom instrument where laser frequency, power, and atomic readout are all governed by the same photonic platform.

In summary, the programmable, agile, PZT-on-SiN resonator combines low optical loss, high tuning efficiency, wideband response, low power consumption, and the ability to operate as a holdover cavity and perform repeatable, rapid, and programmable frequency tuning for atomic quantum experiments. The successful demonstration of sub-Doppler cooling establishes a paradigm for integrated frequency control in atomic physics. By integrating laser stabilization, agile modulation, and atomic referencing on a single photonic platform, this work paves the way toward compact, power-efficient, and scalable cold-atom instruments for quantum sensing and precision metrology.

## Methods

### Device fabrication and packaging

We fabricate the PZT-on-SiN resonator device waveguides on a 15-μm thick lower cladding layer consisting of thermal silicon dioxide (SiO_2_) on a 1 mm thick, 100 mm diameter silicon substrate. Next, we deposit a 120 nm thick stoichiometric silicon nitride layer by low-pressure chemical vapor deposition (LPCVD) directly on top of the thermal oxide layer. We pattern the waveguides in the silicon-nitride layer using a photoresist mask with a 248 nm DUV stepper lithography tool and an inductively coupled plasma etch with CF4/CHF3/O2 gas. Following the pattern etch, we deposit a 4 μm thick layer of SiO_2_ upper cladding by plasma-enhanced chemical vapor deposition (PECVD) with tetraethylorthosilicate (TEOS) used as a precursor. We anneal the wafer at 1050 °C for 7 h and at 1150 °C for 2 h. The PZT actuator stack consists of a sputtered 40 nm thick TiO_2_ adhesion layer, a sputtered 150 nm thick platinum (Pt) bottom electrode, and a 1 μm thick layer of PZT (zirconium-to-titanium ratio of 52:48) deposited via chemical solution deposition (CSD). The stack is capped with a sputtered 100 nm thick Pt top electrode. The PZT and Pt electrodes are patterned by argon ion milling. With the actuator patterned, electrical traces are evaporated and patterned through lift-off and consist of a Cr/Pt/Au stack with thicknesses of 20 and 730 nm, respectively. To reduce resistivity and minimize gold coverage on the electrodes, a 10 μm thick copper layer is electroplated using photoresist molds and a sputtered copper seed layer. The photoresist molds and copper seed layer are removed with solvents to release the device.

We dice the wafer to isolate several ring resonator devices from each wafer reticle. We then bond the die with thermally conductive epoxy on top of a thermoelectric cooler (TEC) mounted inside an aluminum enclosure. We affix a thermistor on the TEC for temperature stabilization. We then align a pair of polarization-maintaining single-fiber v-grooves to the resonator bus and bond them in place with UV-cured epoxy. We make electrical contacts to the PZT actuator via gold wire-bonds to a circuit board with an electrical feed-through to outside the enclosure. During MOT operation and beat-note measurements, a Vescent^TM^ SLICE temperature controller maintains a resonator stability of ≤ 0.1 mK.

The compact, integrated package can facilitate (via a second layer of packaging) additional thermal insulation and vibrational isolation to further mitigate the effects of temperature swings and vibrations if employed in harsh environments, which could not be compensated for by an aluminum package and TEC temperature control alone.

## Supplementary information

Supplementary Information

Transparent Peer Review file

## Data Availability

The data generated in this study support the findings of Figs. 2, 4 and 5 have been deposited in the Zenodo database under accession code https://doi.org/10.5281/zenodo.21817543^38^.

## Code availability

The custom code used to process and plot the data in this study is available in the Zenodo database under accession code https://doi.org/10.5281/zenodo.21817543^38^.
