# Supplementary material for: Sub-Doppler rubidium atom cooling using a programmable agile integrated PZT-on-SiN resonator
Source: Nat Commun. 2026 Sep 16;17:10275. doi: 10.1038/s41467-026-77526-9 (PMC13619608; doi:10.1038/s41467-026-77526-9)
Supplement: Supplementary file 1 [file 41467_2026_77526_MOESM1_ESM.pdf]

Supplementary Materials for

Sub-Doppler rubidium atom cooling using a programmable agile integrated PZT-on-SiN resonator

Andrei Isichenko and Steven Carpenter *et al.*

Corresponding authors:

Jennifer T. Choy (jennifer.choy@wisc.edu) and Daniel J. Blumenthal (danb@ucsb.edu)

This PDF file includes:

Supplementary Notes 1 and 2

Supplementary Figs. 1 to 8

Supplementary Tables 1 and 2

Supplementary Note 1: PZT resonator characterization and operation

Small signal modulation and hysteresis

We characterized multiple ring resonator devices based on lead zirconate titanate on silicon nitride (PZT-on-SiN) from a single wafer, and here summarize representative results for two devices: die 3, which was used for the sub-Doppler cooling demonstration, and die 6, which exhibited the strongest tuning efficiency (Supplementary Fig. 1). We measured the small signal electrical-to-optical response (S_{21}) with a Keysight N5247B PNA-X vector network analyzer connected to the PZT actuator electrodes and a fast photodetector. By tuning the laser to the quadrature point of the optical resonance and biasing the PZT at 3 V, we measured the amplitude-modulation frequency response of the PZT-tuned resonator.

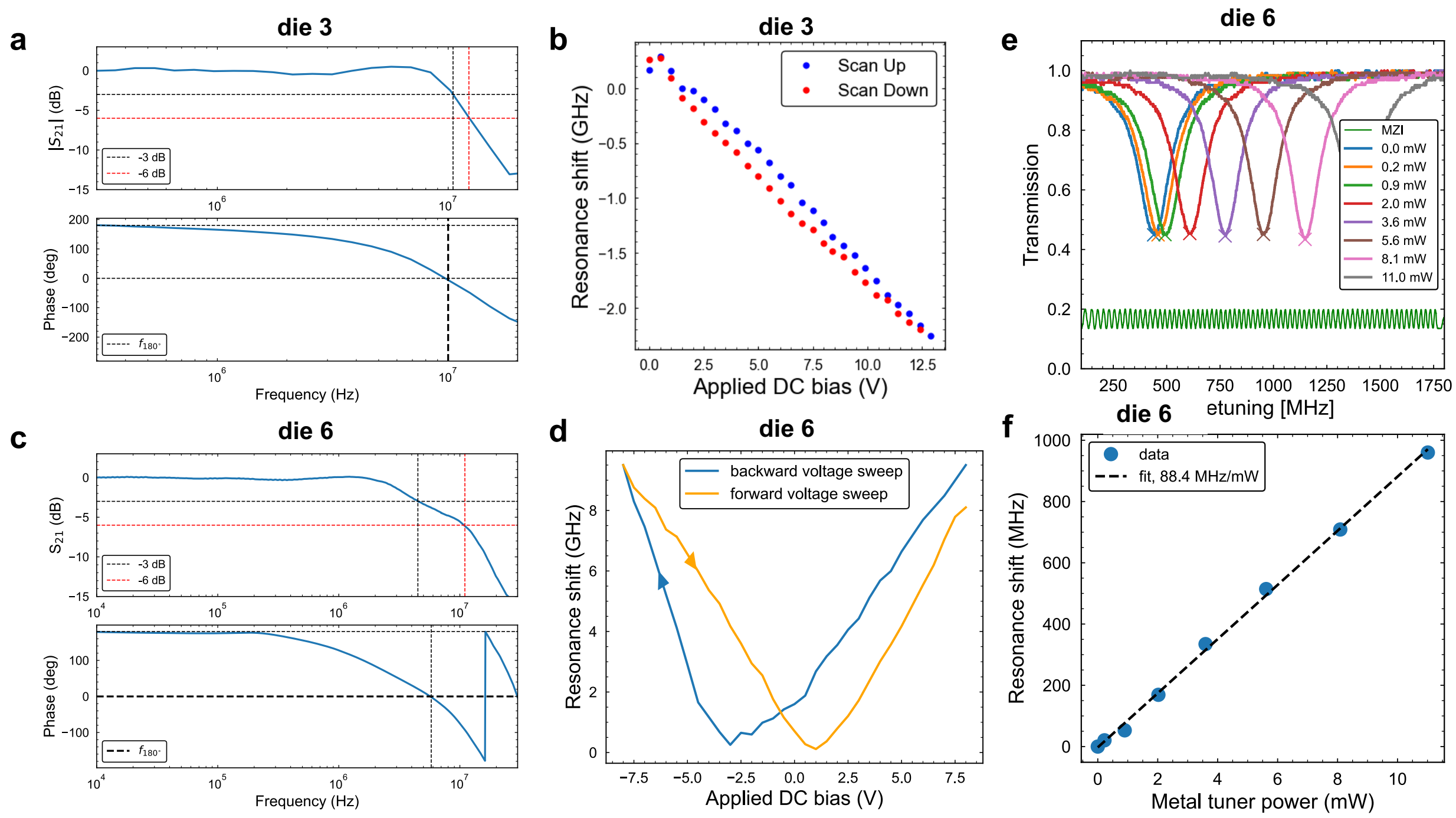

Supplementary Fig. 1: **PZT device characterization. a)** Frequency response measurements of device die 3 used for the sub-Doppler demonstration. The -3 dB and -6 dB response roll-offs are at 10.5 MHz and 12.3 MHz, respectively. The 180 degree phase lag point is at 10 MHz. **b)** Hysteresis measurements for die 3 for positive applied voltage. **c)** Frequency response measurements of device die 6. The -3 dB and -6 dB response roll-offs are at 4.5 MHz and 11 MHz, respectively. **d)** Hysteresis for die 6, for voltage sweeps from negative to positive. **e)** Thermal tuner measurements for die 6, **f)** demonstrating a tuning strength of 88.4 MHz mW^{-1}.

For die 3, the -3 dB and -6 dB response roll-offs occur at 10.5 MHz and 12.3 MHz, respectively, and the 180° phase-lag point is observed at 10 MHz (Supplementary Fig. 1a). For die 6, the -3 dB and -6 dB response roll-offs occur at 4.5 MHz and 11 MHz, respectively, and the 180° phase-lag point is at 5.8 MHz (Supplementary Fig. 1c). The average electrical resistance and capacitance of these PZT ring resonator devices are approximately 2 Ω and 700 pF, respectively, corresponding to an RC-limited electrical bandwidth of $f_e = 1/(2\pi RC) \approx 114$ MHz. The bandwidth limit imposed by the photon lifetime of the cavity is $f_o = c/(\lambda Q_L) = 170$ MHz for die 6. The total modulation bandwidth can be expressed as the combined contribution of the electrical, opto-mechanical (OM),

and optical limits:

$$\frac{1}{f_{\mathrm{BW}}^2} = \frac{1}{f_e^2} + \frac{1}{f_{\mathrm{OM}}^2} + \frac{1}{f_o^2} \tag{S1}$$

This analysis indicates that the measured bandwidth of approximately 11 MHz is limited by the opto-mechanical response of the piezoelectric actuator, rather than by electrical or optical constraints.

The PZT actuator exhibits hysteresis and creep which can affect the absolute frequency of the locked laser after the spectroscopy referencing step. PZT hysteresis is a reversible, history-dependent response between applied voltage and induced strain within a single voltage sweep, while creep describes a time-dependent strain relaxation following a single voltage step[1]. This differs from the single-MHz-scale, minute-scale drift observed in Fig. 5b which is attributed to long-timescale changes in effective mechanical response of the actuator, consistent with accumulated domain evolution and thermal or fatigue-related effects in the PZT material. While hysteresis and creep are endemic to piezoelectric actuation, their effects on frequency positioning can be compensated by closed-loop control[2–4].

Supplementary Figure 1b shows static tuning measurements of die 3, the device used in the sub-Doppler cooling demonstration, where forward (scan-up) and reverse (scan-down) DC voltage sweeps reveal a small bias-dependent hysteresis. This hysteresis manifests primarily as an offset between the two tuning curves, while the tuning slope remains approximately linear over the full voltage range. We also measure the hysteresis tuning curve for die 6 over a larger voltage range, from negative to positive voltages, with different directions for sweep (Supplementary Fig. 1d). This behavior originates from the ferroelectric nature of the PZT, where domain-wall motion and polarization switching lead to a field-history-dependent strain response, particularly near the coercive voltage regime[5]. This results in a history-dependent mapping between applied voltage and laser frequency. During repeated actuation cycles, this effect can lead to slow variations in the cooling laser detuning, which in turn produces observable changes in magneto optical trap (MOT) fluorescence as discussed below.

Thermal tuning

The resonator includes integrated platinum (Pt) thermal tuners. When used with the resonator, these devices exhibit a thermal tuning efficiency of 88.4 MHz mW^{-1} (Supplementary Fig.1e,f).

The tuners consist of 250 nm thick Pt heaters with a width of 5 μm and an edge-to-edge separation of 20 μm from the waveguide. The Pt tuners were not used in the experiments reported here. Instead, frequency control was achieved using the PZT actuator and a thermo-electric cooler (TEC) integrated into the package. Similar Pt thermal tuners were previously employed for large-range frequency control and atomic-referenced laser stabilization in Ref.[6]. In future work, the Pt tuners could serve as a slow trim actuator for compensating long-term frequency drift or PZT hysteresis while reserving the PZT actuator for rapid frequency tuning.

Laser locking

The PZT-on-SiN resonator device is packaged and integrated into the experimental system as shown in Supplementary Fig. 2. The packaged photonic integrated circuit (PIC) is mounted on a temperature-controlled stage and wirebonded to provide electrical access to both the PZT actuator and integrated thermal tuners (Supplementary Fig. 2b,c). Laser stabilization is implemented using a Pound–Drever–Hall (PDH) locking scheme and the schematic of the full laser stabilization architecture is shown in Supplementary Figure 3a. The 795-nm repump laser is stabilized to ^{87}Rb saturation absorption spectroscopy (SAS) via a Red Pitaya field-programmable gate array (FPGA) platform running the open-source Linien laser-locking software[7], which modulates the laser current through a Vescent D2-105 laser controller. The 780-nm cooling laser incorporates a dual-stage laser-to-PIC and PIC-to-SAS locking scheme. The laser-to-PIC lock employs a second Red Pitaya FPGA, which receives a photodiode signal from the output fiber of the PIC resonator and stabilizes the laser to the transmission minimum of the PIC resonance. During operation, the PIC-to-SAS lock provides periodic referencing of the PIC resonator to a second stable ^{87}Rb SAS setup. This lock employs another Vescent D2-105 laser controller and a Vescent D2-125 laser servo. The PIC-to-SAS lock is routinely unlocked and relocked during the experiment to allow the laser frequency to be rapidly driven to different values by shifting the PIC cavity resonance via the PZT element.

To generate error signals for both locking stages, we apply a single 1.2-MHz modulation tone to the 780-nm distributed Bragg reflector (DBR) laser via direct current modulation. This signal is attenuated to ensure operation in the small-signal regime (16 mV$_{\text{pp}}$ at the laser current controller input, which is 16 μA$_{\text{pp}}$ with a 1 mA V^{-1} modulation coefficient, for a 200-mA bias current) which

corresponds to a modulation depth of order 10^{-4}. The modulation depth was chosen to balance a large error signal with minimal added noise, and we confirmed this by comparing the frequency noise of the free-running laser with and without modulation. This tone is used as the local oscillator (LO) demodulation for both locks. The resulting in-loop frequency noise power spectral density (PSD), extracted from the PDH error signal, is shown in Supplementary Fig. 3b. With the laser-to-PIC lock engaged, frequency tuning of the locked laser is achieved by applying a voltage to the PZT actuator. Supplementary Figure 3c shows the error signal obtained while linearly ramping the PZT voltage and scanning across the ^{87}Rb saturation-absorption spectroscopy (SAS) features.

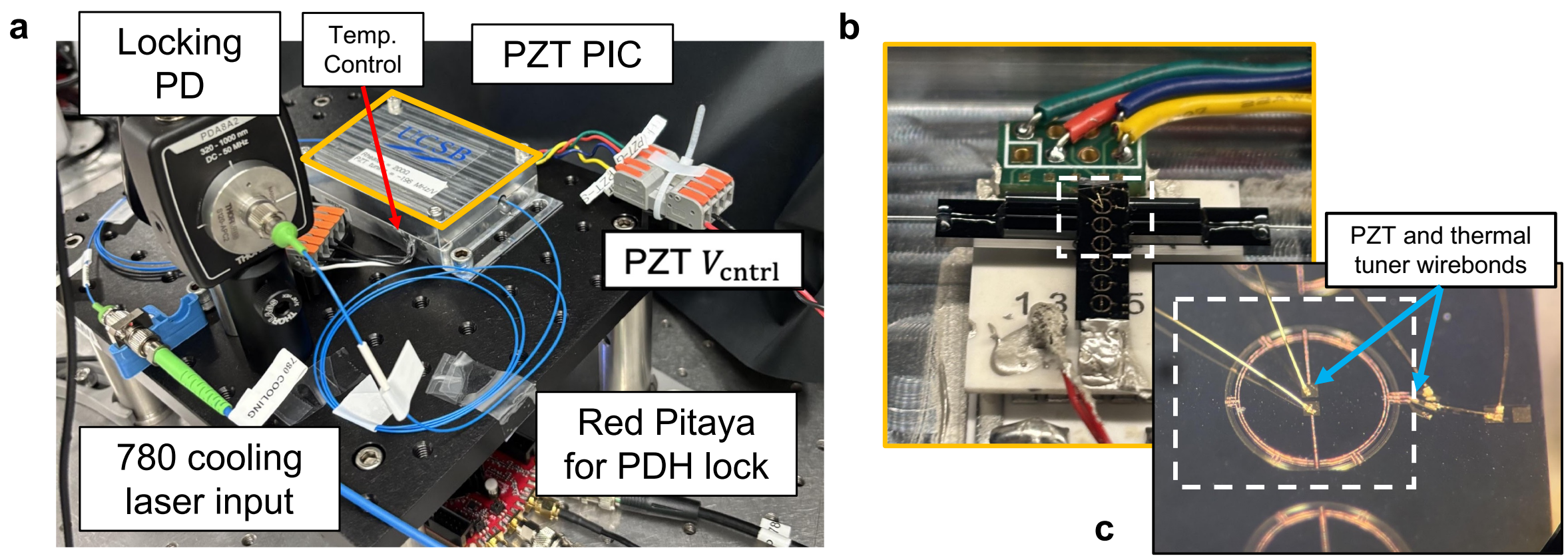

Supplementary Fig. 2: **PZT PIC packaging. a)** Setup for laser stabilization and PZT PIC control, with the packaged PIC inside the enclosure highlighted in orange. The DBR laser (not shown) is external to the PIC package and laser light enters the PIC through an optical fiber. The package is temperature controlled and has inputs for the PZT control. The Red Pitaya FPGA is used as the servo for the 780 laser lock to the PZT PIC resonator. **b)** PZT PIC resonator die with v-groove fiber attachments. The die is bonded to a TEC. **c)** Wirebonded resonator for control of the PZT actuator and thermal tuners.

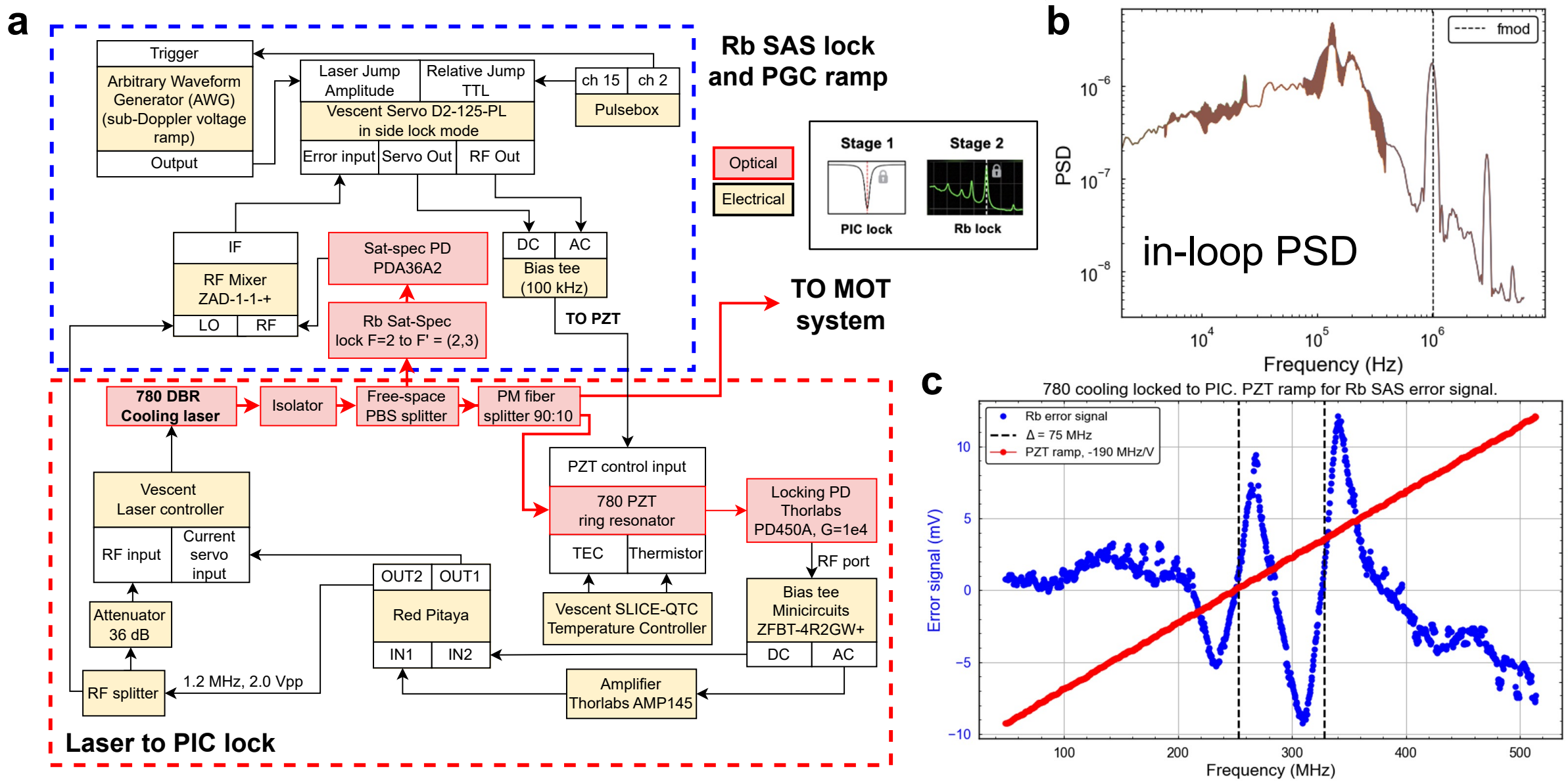

Supplementary Fig. 3: **Laser stabilization. a)** Schematic for both the laser-to-PIC lock and the PZT-to-SAS (^{87}Rb saturation absorption spectroscopy) lock with ramping for polarization gradient cooling (PGC). **b)** In-loop power spectral density (PSD) for the laser-to-PIC Pound-Drever-Hall (PDH) lock. We use a direct-laser-current 1.2 MHz modulation frequency for generating the error signals for both locks. **c)** Error signal for the ^{87}Rb SAS while ramping the PZT with the laser-to-PIC lock engaged. From the known separation of the hyperfine peaks ($F' = (1,3)$ and $F' = (2,3)$, 75 MHz) we extract a frequency tuning strength of -190 MHz V^{-1} which is similar to the measured value in Figure 2. Note that a frequency sweep as shown in this figure is only necessary once when setting up the experiment. When re-referencing during looping experimental cycles, the PZT is simply driven back to a stored voltage setting corresponding to the $F' = (2, 3)$ spectroscopy feature.

The laser frequency-noise spectra measured using both an optical frequency discriminator (OFD) and an independent laser–laser beat note are shown in Supplementary Fig.4a. Locking the DBR laser to the PZT-actuated resonator suppresses low-Fourier-frequency frequency noise relative to the free-running laser, while the residual frequency noise is limited by the modest PDH discriminator slope and servo noise. Based on the cavity linewidth (184 MHz), detected optical power ($\sim 50\mu$W), and agreement between the measured in-loop and out-of-loop noise spectra, we estimate a PDH discriminator slope of approximately 1–5 nV Hz^{-1}. Supplementary Fig.4b further shows that Rb disciplining improves long-term frequency stability for averaging times

above approximately 4 s. We further note that the Rb-disciplined ADEV does not exhibit ideal $\tau^{-1/2}$ scaling because the resonator is only loosely disciplined to the atomic reference. The finite spectroscopy discriminator slope, residual servo error, and environmental perturbations limit the transfer of atomic-reference stability to the resonator, resulting in Allan deviation behavior that differs from that expected for purely white-frequency noise. In addition to frequency stability, rapid and repeatable frequency actuation is required for laser-cooling sequences. Supplementary Fig.4c therefore characterizes the dynamic response of the PZT actuator. A 100-MHz programmed frequency step exhibits a 10-90% rise time of approximately 300 μs and settles within 1.1 ms, confirming the agility required for sub-Doppler cooling sequences.

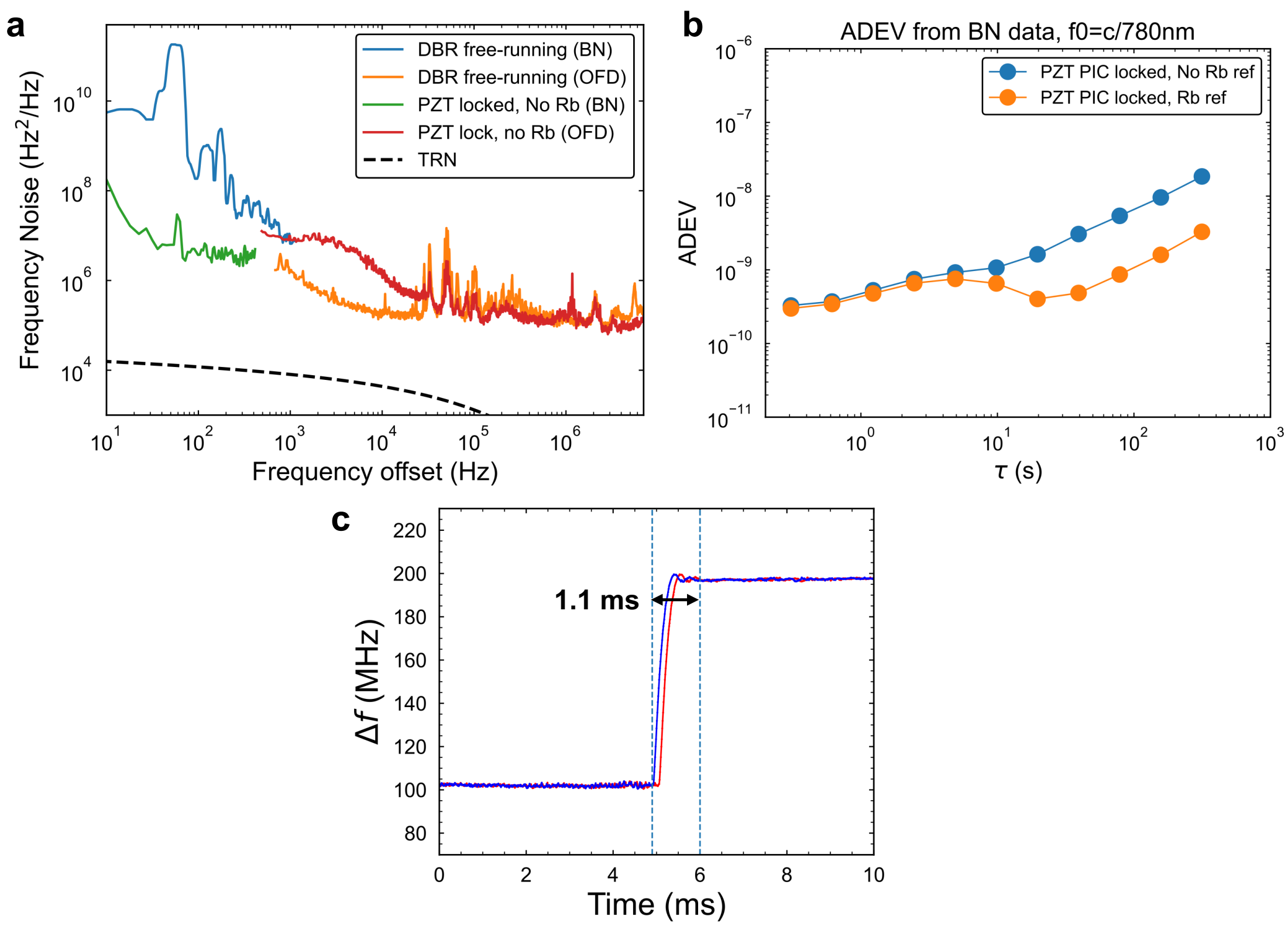

Supplementary Fig. 4: **Frequency-noise, stability, and agility measurements. a)** Frequency-noise spectra of the free-running and PZT-locked 780 nm DBR laser measured using both an optical frequency discriminator (OFD) and a laser–laser beat-note (BN). The PZT lock suppresses low-Fourier-frequency laser noise, while residual frequency noise is limited by the PDH discriminator slope and servo noise. The dashed black curve shows the calculated thermo-refractive noise (TRN) limit of the resonator. **b)** Allan deviation (ADEV) calculated from the beat-note frequency time series of Fig. 5b for operation with and without Rb disciplining. The Rb-disciplined configuration exhibits improved long-term frequency stability for averaging times above approximately 4 s. **c)** Laser beat-note of the PZT-locked laser during a voltage step applied to the PZT actuator measured with a 10 μs frequency counter gate time. A 100 MHz frequency step exhibits a rise time of approximately 300 μs and settles to the final value in approximately 1.1 ms.

.

Optical train power losses

After isolation, the 780-nm cooling DBR laser outputs ~68 mW at the chosen drive current. Of this, 2.4 mW is sent into the SAS branch. The elliptical beam profile of the DBR free-space output limits fiber-coupling efficiencies downstream. The remaining power is sent to the following arms:

- **Cooling arm.** We supply 22 mW of free-space optical power to the fiber coupling into our polarization maintaining (PM) semiconductor optical amplifier (SOA). After amplification, the cooling light is launched back into free space by a collimator, giving a (Gaussian) free-space output power of 34 mW, most of which is bound for the atom trap. Separately, we deliver 3.5 mW of 795-nm repump light to the atom trap.

- **Resonator arm.** We supply 16 mW of free-space optical power to the resonator arm. The light is coupled into a fiber and delivered toward the resonator through a fiber-to-fiber coupling, at which point we measure 1.4 mW; the coupler itself adds a further 20% loss. The packaged device then has a fiber-to-chip coupling loss of 9 dB, higher than our typical 3-4 dB due to poor modal matching between the fiber mode and the PIC. The 900-nm waveguide width was not optimized for fiber coupling at the time of the device design. Adding a 250-nm taper is estimated to reduce the fiber-to-chip loss to around -3 dB and future devices for this SiN thickness will use this taper.

- The rest of the power is delivered to a beam dump.

Supplementary Note 2: MOT system characterization and measurement

We used a six-beam (three beams with retro-reflection) free-space ^{87}Rb MOT. The 780-nm laser drives a red-detuned ^{87}Rb $5^2S_{1/2}$ $F = 2 \rightarrow 5^2P_{3/2}$ $F' = 3$ transition, while a separate 795-nm DBR laser acts as the repump, addressing the ^{87}Rb $5^2S_{1/2}$ $F = 1 \rightarrow 5^2P_{1/2}$ $F' = 2$ transition. The relevant energy level transitions are shown in Supplementary Fig. 5. Our system uses a home-built 2D printed circuit board (PCB) coil originally inspired by Chen et al.[8]. The coils are switched

by a custom circuit with $\sim$ 100 μs switching time. In all configurations, we use a SOA (Thorlabs BOA780P) for cooling power control and shuttering.

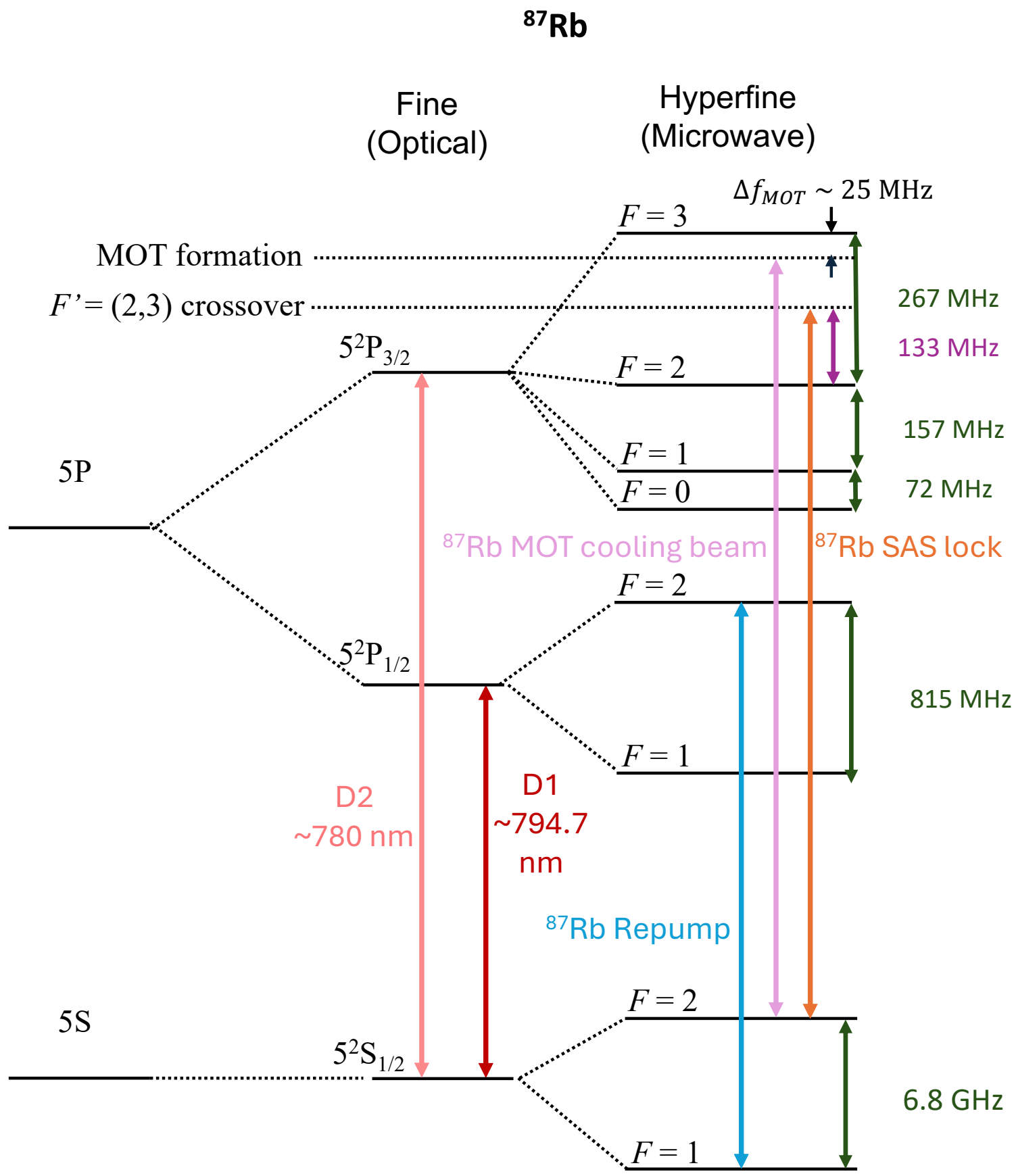

Supplementary Fig. 5: **Energy level diagram.** Energy level diagram of ^{87}Rb including the D_1 (light red) and D_2 (dark red) transitions and the hyperfine structure. Relevant experimental laser frequencies are color coded: the 795-nm repump beam (light blue), and the 780-nm MOT cooling beam (pink, red-detuned by 25 MHz) and the ^{87}Rb saturation absorption spectroscopy (SAS) lock frequency (orange). During the MOT formation, the cooling laser rapidly switches between the green and orange frequency settings by means of the PZT-actuated resonator.

System configurations in this work

We performed sub-Doppler cooling using four hardware and control configurations, summarized in Figure 4a. A reference configuration, labeled Case IV in Figure 4, employed an AOM (see Supplementary Fig. 6). In this conventional setup, the laser was locked to the ^{87}Rb, $5^2S_{1/2}, F$ =

$2 \rightarrow 5^2P_{3/2}, F' = (1,3)$ crossover and double-passed through an AOM driven by ~95-MHz RF, yielding a ~22-MHz red-detuning from the cooling transition. Both frequency and intensity ramps were applied by modulating the AOM drive and SOA bias, respectively. Figure 4 shows the TOF data for the coldest ^{87}Rb cloud achieved in this configuration.

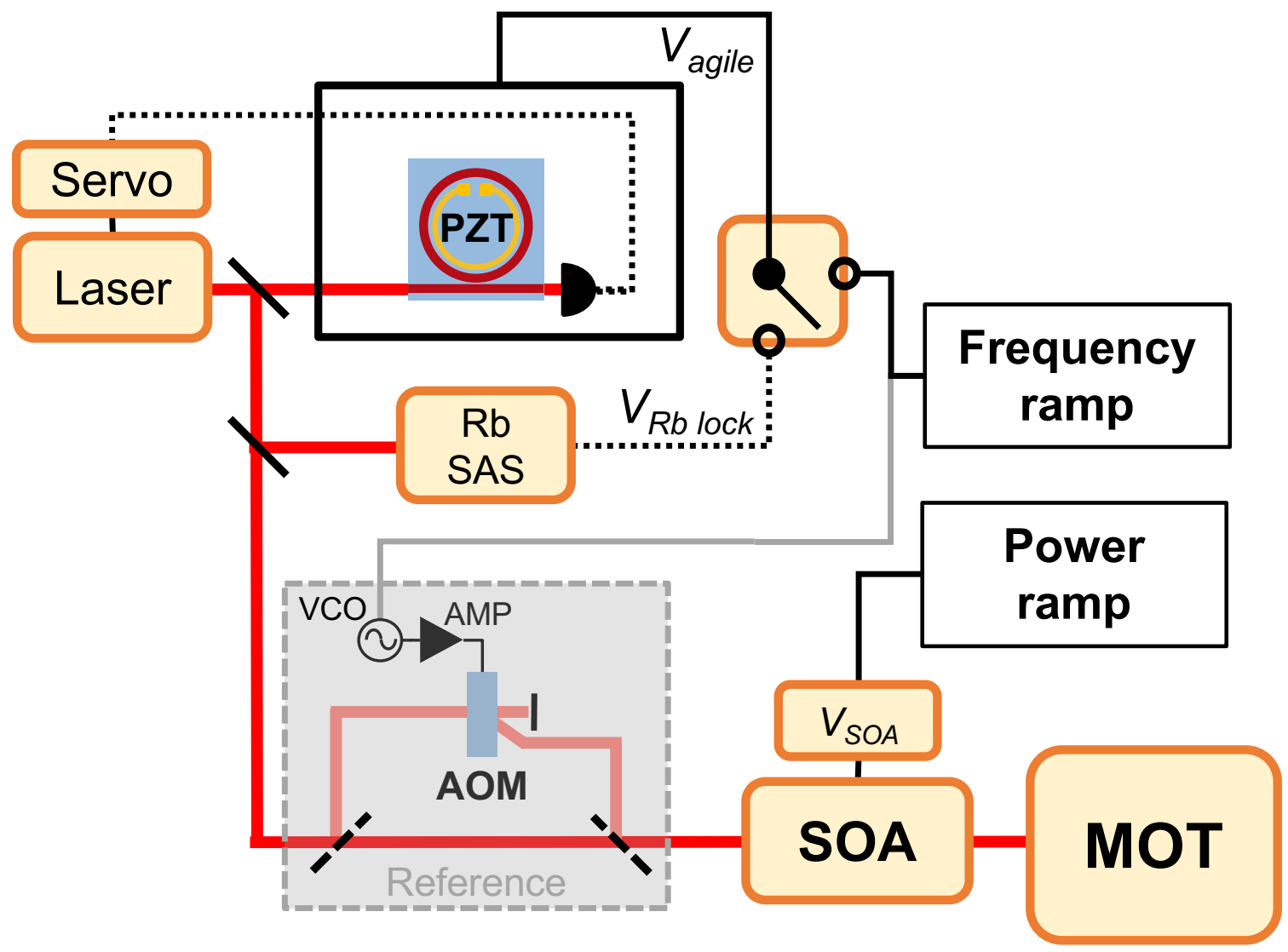

Supplementary Fig. 6: **MOT system configurations for temperature measurements.** The 780-nm cooling laser is locked to the frequency-agile PZT cavity. The laser tracks the resonance frequency of the cavity, which is itself either tied to ^{87}Rb SAS or to the applied frequency ramp signal. The power ramp signal modulates the cooling beam power delivered to the MOT via current through the semiconductor optical amplifier (SOA). MOT temperatures were recorded for four cases: (I) the MOT without PGC (i.e. without any frequency or power ramp), (II) PZT-controlled frequency ramp (without any power ramp), (III) PZT-controlled frequency ramp and SOA-controlled power ramp, and (IV) AOM-controlled frequency ramp and SOA-controlled power ramp (AOM reference). In each case identical parameters were used for sequence timing, cooling and repump frequencies and powers, and fluorescence detection. For cases I, II, III the AOM is not used. The AOM is powered by an RF amplifier (AMP) and a voltage controlled oscillator (VCO).

All other configurations (I, II, III) employ the dual-stage lock (laser-to-cavity and cavity-to-^{87}Rb SAS) with the AOM removed from the optical path. In each case, the optical cavity was locked to the ^{87}Rb, $5^2S_{1/2}, F = 2 \rightarrow 5^2P_{3/2}, F' = (2,3)$ crossover (note the distinction from the $F' = (1,3)$ crossover of configuration IV) during the ^{87}Rb-disciplined portion of the experiment (Fig. 3c). After

223 ms of MOT formation, either a 12 ms polarization-gradient cooling (PGC) ramp sequence was applied (II and III) or the MOT was held constant (I). The atomic cloud temperature is determined from time-of-flight (TOF) imaging (more information below). Two experimentally optimized PGC setups are tested: frequency-only ramping (II), where the laser was further red-detuned via a voltage ramp on the PZT actuator, and combination frequency and intensity ramping using the PZT and SOA, respectively (III). For comparison, Figure 4a includes the AOM-based configuration. The integrated tunable-cavity approach provides a larger and more stable (in optical power) tuning range without affecting beam alignment, offering a compact and power-efficient alternative for sub-Doppler atom cooling.

Atom number calculation

MOT fluorescence, in combination with other experimental conditions, can be used to determine the number of trapped atoms:

$$N = \frac{1}{R_{\text{scat}}} \frac{\mathrm{d}N_\gamma}{\mathrm{d}t}, \tag{S2}$$

where $\frac{\mathrm{d}N_\gamma}{\mathrm{d}t}$ is the rate of photons emitted over the entire sphere in a unit time. The photon scattering rate R_{scat} is given by[9]

$$R_{\text{scat}} = \frac{\Gamma}{2} \frac{\frac{I_{\text{tot}}}{I_{\text{sat}}}}{1 + \frac{I_{\text{tot}}}{I_{\text{sat}}} + 4\left(\frac{\Delta_{\text{MOT}}}{\Gamma}\right)^2} \tag{S3}$$

where $\Delta_{\text{MOT}} = 2\pi\, \delta f_{\text{MHz}}$, and δf_{MHz} is the frequency detuning in MHz of the cooling laser with respect to the ^{87}Rb $5^2S_{1/2}\, F = 2 \rightarrow 5^2P_{3/2}\, F' = 3$ transition. I_{tot} and I_{sat} are the total cooling beam intensity and the saturation energy, respectively. We used a plano-convex lens just outside our glass vacuum chamber to collect light. This light was imaged with a traditional compound camera lens onto the sensor of our Thorlabs CS135MU camera.

From the digital images captured by the camera, we can determine $\frac{\mathrm{d}N_\gamma}{\mathrm{d}t}$ by summing the (background-subtracted) pixel counts in our region of interest ($C_{x,y}$) from an exposure and applying conversion factors:

$$\frac{\mathrm{d}N_\gamma}{\mathrm{d}t} = \frac{4\pi \left(\sum_{ROI_{x,y}} C_{x,y}\right) G}{\Omega\, \eta_{\text{QM}}\, \eta_{\text{geo}}\, \Delta t_{exp}}, \tag{S4}$$

where G is the gain coefficient of the camera sensor, η_{QM} is the quantum efficiency of the camera at 780 nm, η_{geo} accounts for a geometric loss in the light collection cone from an obstruction in the

imaging path, Ω is the solid angle of light collection, and Δt_{exp} is the duration of camera exposure. The values for Ω and η_{geo} were found by careful measurement of the setup and simulation in Zemax OpticStudio™. Supplementary Table 1 shows the experimental parameters and uncertainties used in the calculation of the atom number.

Atom number uncertainty was computed via Monte Carlo propagation. Each experimentally uncertain parameter (camera gain and quantum efficiency were neglected) is sampled from independent Gaussian distributions centered on the measured value and with standard deviation given by estimated uncertainty. The full atom number is repeatedly computed from the sampled parameter set. Reported atom number is taken as the mean of this distribution and the associated uncertainty is taken as its standard deviation. This approach avoids linearization assumptions which perform poorly in the highly nonlinear scattering expression.

Atom number uncertainty (20.3% in total) was dominated by systematic error in imaging calibration with slight contribution from shot-to-shot uncertainty in imaging light intensity and detuning.

When capturing MOT images just before PGC (at $\tau_{\text{seq}} = 237$ ms as seen in Supplementary Figure 7), we determined an atom number of ~ 5 million (with 20% uncertainty). However, the MOT is loaded for only 227 ms, which is insufficient for full reloading (despite partial atom recapture) between cycles. Longer loading times would increase the atom number but reduce the achievable sampling rate in a full quantum sensor.

Supplementary Table 1: Experimental parameters used in the determination of MOT atom number.

Parameter	Explanation	Value	Ref.
λ	^{87}Rb D2 transition wavelength	780.241 nm	9
Γ	^{87}Rb natural linewidth for the D_2 ($5^2S_{1/2} \rightarrow 5^2P_{3/2}$) transition	2π (6.066 MHz)	9
σ_0	^{87}Rb resonant cross section intensity for the $F = 2 \rightarrow F' = 3$ and isotropic light polarization	1.36×10^{-12} cm^2	9
I_{sat}	^{87}Rb saturation intensity for the $F = 2 \rightarrow F' = 3$ transition with isotropic light polarization	3.577 mW cm^{-2}	9

Parameter	Explanation	Value	Ref.
Δ_{MOT}	(Angular) frequency detuning from the $F = 2 \longrightarrow F' = 3$ transition (for configurations I, II, and III). Determined by beat-note measurements	-2π (25.5 ± 0.2 MHz)	
$P_{\mathrm{x,y}}$	Average power of the x- and y-axis cooling beams measured just before entering the cell	6.22 ± 0.05 mW	
P_{z}	Average power of the z-axis beam measured just before entering the cell. Since this branch goes through a beam expander, and is cut off by the aperture of the PCB coils (6.5 mm diameter), this value is measured after the aperture	1.00 ± 0.05 mW	
$A_{\mathrm{x,y}}$	Area of the x- and y-axis cooling beams, using the $1/e^2$ beam width of the Thorlabs F810APC-780 collimator	0.442 cm^2	10
A_{z}	Area of z-axis cooling beam, using the width of the PCB coil aperture (6.5 mm diameter)	0.332 cm^2	
$I_{\mathrm{x,y}}$	Average intensity of x- and y-axis cooling beams before the glass cell	$P_{\mathrm{x,y}}/A_{\mathrm{x,y}} =$ 14.07 ± 0.11 $\mathrm{mW\,cm^{-2}}$	
I_{z}	Average intensity of z-axis cooling beam	$P_{\mathrm{z}}/A_{\mathrm{z}} =$ 3.00 ± 0.15 $\mathrm{mW\,cm^{-2}}$	
T_{cell}	Measured transmission through a layer of the uncoated glass vacuum cell at 45° incidence	0.96 ± 0.01	
I_{tot}	Sum of the intensities of the six trapping beams at the location of atoms. Three input beams and three retro-reflected (RR) beams. The z-axis beam passes through the AR cell at normal incidence with negligible reflections. Assume perfect retro-reflection	$2T_{\mathrm{cell}}\, I_{\mathrm{x,y}} + 2T_{\mathrm{cell}}^2\, I_{\mathrm{x,y}} + 2I_{\mathrm{z}} =$ 59.0 ± 1.0 $\mathrm{mW\,cm^{-2}}$	

Parameter	Explanation	Value	Ref.
Ω	Solid angle of collected light. This value was calculated from experimental measurements and lens parameters simulated in Zemax OpticStudio. The resulting half-angle collection cone was 6.0°	0.035 ± 0.006 sr	
η_{geo}	Geometrical light collection efficiency, assuming the transmission through the B-coated lenses are nearly 1 (this light is collected at ≤ 6° from normal incidence). This loss is the result of slight clipping of the collection cone by an optical mount	0.74 ± 0.5	
η_{QM}	Camera (Thorlabs CS135MU) quantum efficiency at λ = 780 nm	0.34 e^- per photon	11
G	Camera sensor (onsemi NOIP1SN1300A-QTI) gain coefficient. LSB means a single "Least-Significant Bit" flip	10.42 e^- per LSB	12
Δt_{exp}	Camera exposure time	0.5 ms (single-shot), 5 ms (10x accumulation)	

Temperature measurement

The temperature of our ^{87}Rb MOT is determined by imaging the ballistically expanding atom cloud with a 0.5 ms exposure for a series of different τ_{TOF} (time-of-flights). At $\tau_{\text{TOF}} = 0$, we shutter both cooling and repump lasers and rapidly shut off the magnetic coils. Note that we use an earlier coil shutoff for PGC. Timing information for our experimental determination of the MOT temperature is shown in Supplementary Fig. 7.

For six distinct τ_{TOF} settings (1, 2, 3, 4, 5, 6 ms), we collected 10-shot accumulated, background-subtracted fluorescence images and fit the cloud widths to the linearized ballistic expansion equations:

$$\sigma_y^2(\tau_{\text{TOF}}) = \sigma_{y,0}^2 + \frac{k_{\text{B}}T_y}{m}\,\tau_{\text{TOF}}^2, \qquad \sigma_z^2(\tau_{\text{TOF}}) = \sigma_{z,0}^2 + \frac{k_{\text{B}}T_z}{m}\,\tau_{\text{TOF}}^2, \tag{S5}$$

where $\sigma_y(\tau_{\text{TOF}})$, $\sigma_z(\tau_{\text{TOF}})$ are the standard deviations of Gaussian fits to the cloud images in each

dimension (after reducing to one-dimensional profiles by summing along the orthogonal image axis), l_{px} is the calibrated pixel size, k_B is the Boltzmann constant, m is the mass of ^{87}Rb, and T_y,T_z are the MOT temperatures along the y,z axes.

The procedure was performed independently for each of the two image axis, yielding fitted widths σ_y and σ_z. The covariance matrix from the Gaussian fit was used to estimate the statistical fitting uncertainty in each extracted width is the square root of the covariance matrix element $\text{Cov}(\sigma, \sigma)$. To avoid underestimating the uncertainty from fitting a smooth image, a conservative one-pixel extraction floor was added in quadrature to the Gaussian fit uncertainty.

The image scale was calibrated using a USAF resolution target, yielding a 12.86 μm/pixel. Based on a spread among measurements of multiple features with manual extraction, a conservative 2% uncertainty was assigned to the calibration. Because the extracted temperature scales as the square of the length calibration, this contributes a 4% systematic uncertainty to the atom temperature. This calibration uncertainty is not included in the individual data-point error bars, since it is common to all measurements and therefore systematic. It is, however, included in the final reported temperature uncertainty according to

$$\delta T_{tot} = \sqrt{\delta T_{\text{fit}}^2 + \delta T_{\text{cal}}^2} \tag{S6}$$

Supplementary Table 2 lists the relevant values for these calculations.

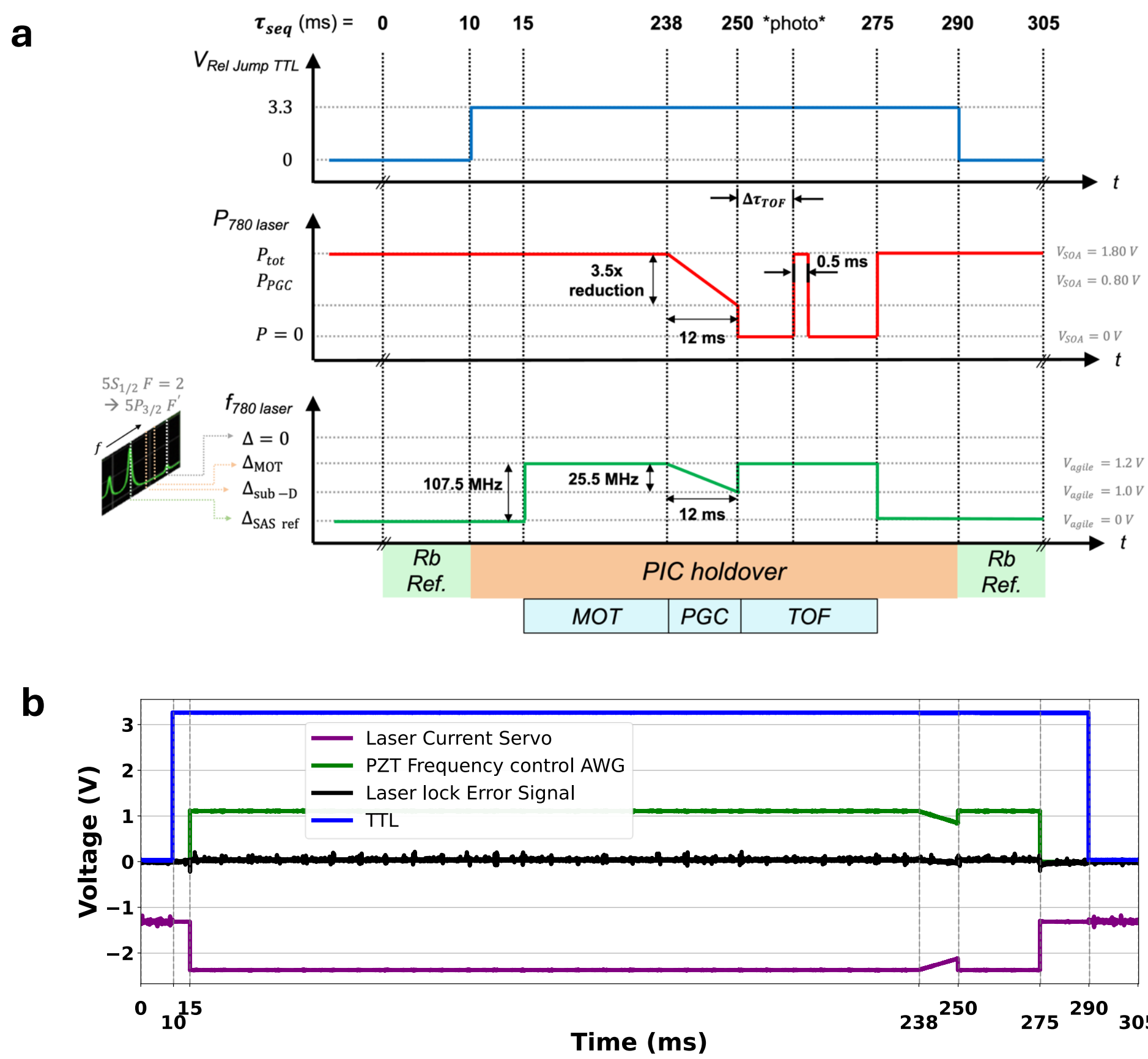

Supplementary Fig. 7: **Experiment timing. a)** Timing diagram for configuration III of Figure 4 and Supplementary Fig. 6. All time-of-flight (TOF) measurements are performed within a repeating 305 ms control sequence, with the TOF delay τ_{TOF} varied between runs. SAS re-referencing occurs during two 25 ms windows at the beginning and end of each cycle when the relay-jump transistor-transistor logic (TTL) pulse is low. MOT loading begins at τ_{seq} = 10 ms and continues until 238 ms for sequences including PGC, or until 250 ms when PGC is omitted. During PGC, the magnetic field is switched off at 238 ms (250 ms without PGC) and remains off until 275 ms. At τ_{seq} = 250 ms, the cooling and repump beams are shuttered via gain switching of the SOA (current modulation), achieving an optical on/off ratio of 55 dB, and the atomic cloud subsequently undergoes ballistic expansion for a variable τ_{TOF}. Imaging is performed using a 0.5 ms camera exposure, after which the laser to the cold atoms is optically shuttered and is returned to the SAS reference point to prepare for the next cycle. **b)** Monitoring signals during the sequence, showing the lock error signal black, which confirms that the laser remains locked to the PZT-stabilized cavity throughout the cycle. In comparison with a), the blue traces (TTL) and green traces (laser frequency control) are complementary. The x-axis shows vertically staggered values for legibility.

Supplementary Table 2: Parameters used in the determination of atom temperatures

Parameter	Explanation	Value	Ref.
k_B	Boltzmann constant	$1.380649 * 10^{-23}$J K^{-1}	
$m_{^{87}\text{Rb}}$	^{87}Rb isotope mass	$1.44316060(11) * 10^{-25}$ kg	9
Δt_{exp}	Camera exposure time	0.5 ms (single-shot), 5 ms (10x accumulation)	
l_{px}	USAF target calibrated pixel size (with 2% error)	12.86 ± 0.26 μm px^{-1}	

Atom-number drift arising from PZT frequency-response drift

Figure 5b in the main text shows MOT atom number measurements (red curve) over several minutes using two schemes: either periodic referencing to ^{87}Rb spectroscopy (upper plot) or halting ^{87}Rb-referencing at $t = 0$ (lower plot). The extended dataset for the former case is shown in Supplementary Fig. 8a, where we simultaneously record the beat-note frequency (blue curve) between the 780-nm DBR cooling laser and a separate 780-nm DBR locked to the ^{87}Rb, $5S_{1/2}, F = 2 \rightarrow 5P_{3/2}, F' = 1, 3$ crossover. The left axis shows the normalized mean squared error (NMSE) of the beat-note signal for each MOT + PGC sequence with respect to the sequence for $t = 0$.

Without ^{87}Rb-referencing (Fig. 5b, lower), the MOT completely dissolves within 30 seconds due to the drift of the PZT cavity-locked laser. With ^{87}Rb-referencing, residual frequency excursion can be attributed to the drift in the PZT cavity voltage-to-frequency response at a fixed voltage jump. As shown in Supplementary Fig. 8a, the isolated effect of PZT hysteresis can be observed: the MOT is still visible for over 20 minutes, though temporal shifts in PZT response result in the loss of ~2/3 of trapped atoms over that time. Without ^{87}Rb-referencing, the cooling laser exhibits a ~50-MHz drift over 20 minutes, whereas with ^{87}Rb-referencing, the drifts are reduced to the single-MHz range over the same interval. For precision quantum applications such as computing or sensing, such laser frequency drifts must be minimized. To address this, future work will implement dynamic adjustment of the PZT voltage to counteract these drifts, initially using spectroscopy signals and then ultimately using MOT fluorescence itself for feedback.

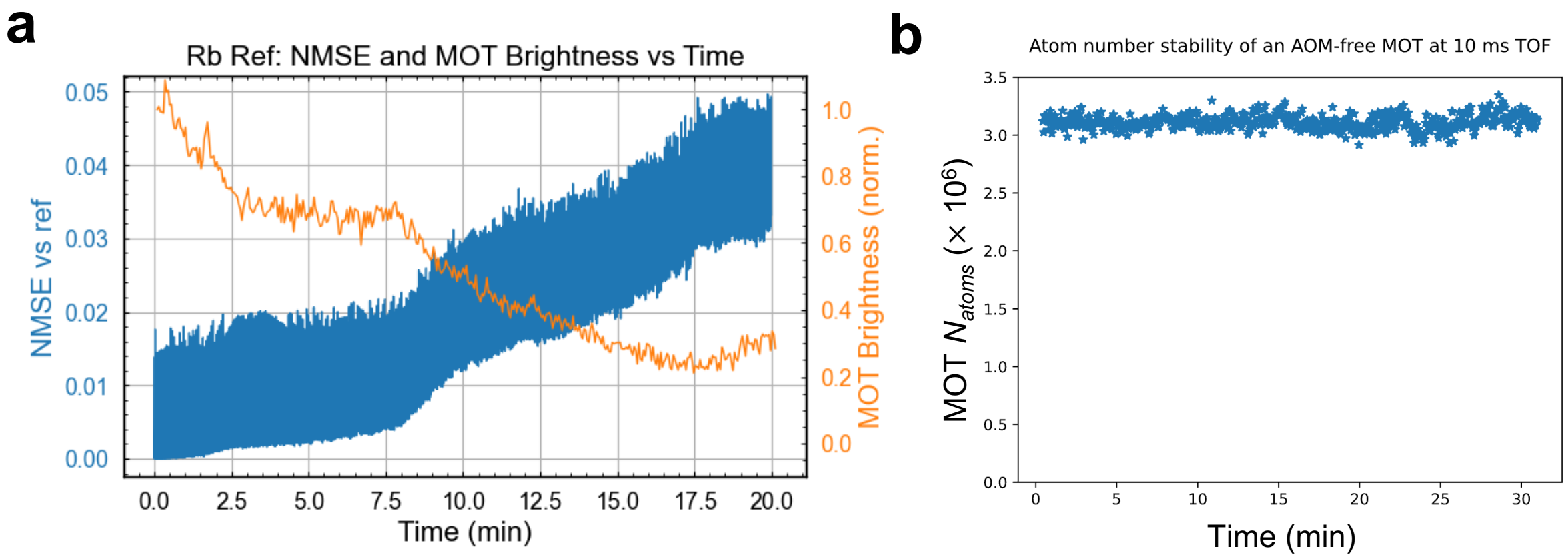

Supplementary Fig. 8: **PZT Hysteresis and atom number stability. a)** Extended time-series data for Figure 5b. Normalized mean squared error (NMSE) of the beat note between the cooling and reference lasers (blue) and number of atoms in the MOT (orange) measured over the same time interval. The NMSE is calculated between the frequency trace of each sequence w.r.t. the first sequence. The slow upward drift of the NMSE corresponds to changes in the PZT voltage-to-frequency actuation. Variations in the trapped atom number correlate with this drift. Note that the atom number uncertainty (calculated via Monte Carlo propagation, see Supplementary Materials section "Atom number calculation") is dominated by systematic error in imaging calibration and is 20.3%. This constant errorbar is neglected from the plot for clarity. In this test, the MOT operates with reduced cooling beam power owing to light diverted for the simultaneous beat-note measurement. **b)** Continuous 30-minute record of MOT atom number using the PZT cavity for spectroscopy referencing and for PGC, during which the PZT hysteresis was negligible. This data was collected after powering-on the system for over two hours. This warm-up time allows for the stress-optic response of the PZT to better stabilize, though this level stability is not guaranteed on hours-long timescales. Future work will compensate for the time dependence of PZT drive voltage to resonance shift by means of a slow trim loop using MOT fluorescence as an error signal.[13] Atom number uncertainty is again 20.3% which is dominated by systematic error in imaging system calibration. The constant error bar is again neglected from the plot for clarity.

We note that PZT frequency-response drift is temporally variable, typically strongest shortly after system power-up. The data in Figure 5b was collected during this initial phase. On a separate day, after several hours of continuous operation, the MOT brightness remained stable for 30 minutes

(Supplementary Fig. 8b). In this trial, the cavity was periodically referenced to ^{87}Rb spectroscopy (as in Fig. 5a) and our typical 12 ms power and frequency PGC ramp was performed. The atom number imaging took place after at 10-ms time-of-flight. Although beat note data was not collected for this trial, the stability of atom number directly corresponds to a stable absolute laser frequency, suggesting the anticipated system performance once dynamic PZT control is implemented.

Supplementary References

[1] Devasia, S., Eleftheriou, E. & Moheimani, S. O. R. A survey of control issues in nanopositioning. *IEEE Transactions on Control Systems Technology* **15**, 802–823 (2007).

[2] Wang, X., Budinger, V. & Gourinat, Y. A specific methodology of creep compensation for piezoelectric actuators by open-loop control. *Applied Mechanics and Materials* (2013).

[3] Nie, Z., Cui, Y., Huang, J., Wang, Y. & Chen, T. Precision open-loop control of piezoelectric actuator. *Journal of Intelligent Material Systems and Structures* **33**, 1198–1214 (2022).

[4] Sabarianand, D. V., Karthikeyan, P. & Muthuramalingam, T. A review on control strategies for compensation of hysteresis and creep on piezoelectric actuators based micro systems. *Mechanical Systems and Signal Processing* (2020).

[5] Nguyen, M. D., Houwman, E. P. & Rijnders, G. Large piezoelectric strain with ultra-low strain hysteresis in highly c-axis oriented Pb(Zr0.52Ti0.48)O3 films with columnar growth on amorphous glass substrates. *Scientific Reports* **7**, 12915 (2017).

[6] Isichenko, A. *et al.* Multi-laser stabilization with an atomic-disciplined photonic integrated resonator. *Optica Quantum* **4**, 232–240 (2026).

[7] Wiegand, B., Leykauf, B., Jördens, R. & Krutzik, M. Linien: A versatile, user-friendly, open-source FPGA-based tool for frequency stabilization and spectroscopy parameter optimization. *Review of Scientific Instruments* **93**, 063001 (2022).

[8] Chen, L. *et al.* Planar-Integrated Magneto-Optical Trap. *Physical Review Applied* **17**, 034031 (2022).

[9] Steck, D. A. Rubidium 87 D Line Data. Tech. Rep., Los Alamos National Laboratory (2001).

[10] Thorlabs, Inc. F810APC-780 FiberPort, FC/APC Coupler, 780 nm Design Wavelength. Product specification, Thorlabs (2021).

[11] Thorlabs, Inc. *Compact Scientific Digital Cameras — User Guide (CS Series)* (2025).

[12] Semiconductor Components Industries, LLC. NOIP1SN1300A/D – Python 1.3/0.5/0.3 megapixels global shutter CMOS image sensors: Data sheet. Technical data sheet NOIP1SN1300A/D, ON Semiconductor, Phoenix, Arizona, USA (2021).

[13] Hinton, A. *et al.* A portable magneto-optical trap with prospects for atom interferometry in civil engineering. *Philosophical Transactions of the Royal Society A: Mathematical, Physical and Engineering Sciences* **375**, 20160238 (2017).
