# Supplementary material for: Sub-Doppler rubidium atom cooling using a programmable agile integrated PZT-on-SiN resonator
Source: Nat Commun. 2026 Sep 16;17:10275. doi: 10.1038/s41467-026-77526-9 (PMC13619608; doi:10.1038/s41467-026-77526-9)
Supplement: Supplementary file 2 [file 41467_2026_77526_MOESM2_ESM.pdf]

nature portfolio

Peer Review File

Sub-Doppler rubidium atom cooling using a programmable agile integrated PZT-on-SiN resonator

Corresponding Author: Professor Daniel Blumenthal

This file contains all reviewer reports in order by version, followed by all author rebuttals in order by version.

Attachments originally included by the reviewers as part of their assessment can be found at the end of this file.

Version 0:

Reviewer comments:

Reviewer #1

(Remarks to the Author)
The manuscript entitled "Sub-Doppler rubidium atom cooling using a programmable agile integrated PZT-on-SiN resonator" by Andrei Isichenko et al. investigates the use of a high Q SiN ring resonator, whose resonance frequency can be dynamically tuned as a cavity to which an external DBR laser is locked to provide holdover between referencing to rubidium spectroscopy Sub-Doppler rubidium atom cooling. The experimental demonstrations are carefully described in the manuscript and most of the questions that I had were answered in the manuscript or the supplementary information. The manuscript is well structured and accessible for a broad of the photonic community. The work is of interest to the wider photonic integrated circuit and quantum sensing community as it is a step toward the size and power reduction of next generation quantum photonic technologies. As such I am supportive to publish this work in Nature Communications after addressing the following comments:
1) Was there any reason why the authors did not directly self-injection locking experiments of the laser to the PZT cavity? It feels like the consequential next step that would simplify the setup and reduce the photonic footprint significantly. Currently, it feels a bit strange to have only the piezoelectric controllable cavity on chip and everything else is fiber coupled to it. It would be good to discuss this. (I note that it is pitched in the outlook section)
2) Could the authors discuss the residual amplitude modulation that they get when modulating the drive current of the laser?
3) Fig. 2b). It seems that the oscillations of the unbalanced Mach-Zehnder interferometer are non-period in the narrow frequency sweep. Why is this the case? I find this somewhat surprising.
4) Fig. 2e) Can the authors add a sentence to discuss why the piezo tuning curve is quite nonlinear at low voltage levels?
5) Fig. 4 caption. The authors used c), I believe that they mean b)? Also, I find the timing information confusing. It is labeled that it was a 6ms, but the arrow from the data point in the graph indicates 36ms. Which one is it?
6) Supplementary information, Section 1: Can the authors discuss where the observed hysteresis is originating from?
7) Fig. 2S: Is the DBR laser also in the aluminum box or is it external to it? I was trying to find it, but it was not clear to me.

Reviewer #2

(Remarks to the Author)
In this manuscript, the authors demonstrate sub-Doppler cooling of ^{87}Rb atoms in a 3-D magneto-optical trap (MOT) using a sideband-free scheme based on a fully integrated, low-power, fast-tunable optical frequency reference instead of bulk AOMs/EOMs, especially for cooling and detection beams. Their integrated, PZT-tunable Si_3N_4 ring resonator (Q ≃ 2–3 × 10^6) serves both as a short-term frequency reference and as a fast, low-power frequency shifter for a commercial-off-the-shelf (COTS) 780 nm laser. The laser is Pound-Drever-Hall (PDH) locked to the high-Q resonator, whose resonance is itself periodically disciplined to an 87Rb saturation-absorption-spectroscopy (SAS) feature (the F = 2 → F' = 2, 3 crossover) to ensure long-term stability. During each cooling cycle, the SAS serve is temporarily gated off, and programmable voltages on the PZT actuator shift the cavity resonance with low power (≲10 nW)—and hence the locked laser—through the tens-of-MHz detunings needed for MOT loading, followed by a sub-Doppler polarization-gradient-cooling (PGC) ramp (15 MHz over 12 ms), while a COTS semiconductor optical amplifier (SOA) supplies intensity control. In their device characterization, the ring provides ≃10 GHz of total tuning (≈1 GHz/V) with an 11 MHz small-signal bandwidth. Using this sideband-free scheme —in which no bulk AOMs, EOMs, or multi-watt RF drivers are required—the authors cool atomic clouds to 16 μK and confirm

atomic temperatures and atom numbers through several methods: PIC-based laser cooling for a steady-state MOT, PIC-based frequency ramp only, PIC-based frequency ramp & SOA intensity control, and conventional AOM-based frequency ramp & SOA intensity control.

This multidisciplinary work between atomic physics and integrated photonics is impressive and represents a significant step toward fully integrated, PIC-based cold-atom systems by eliminating bulky, high-power modulator chains. They showed (1) dual-stage PDH and SAS locking in a single PIC package with cycle-to-cycle repeatability; (2) atom temperature comparison and beat-note stability data, and device characterization, as well as a path shown toward a full PIC driven laser system.

I recommend accept with major revisions for publication in Nature Communications. The authors may need to present several results more quantitatively. In particular, although a companion manuscript (Ref [23], doi:10.48550/arXiv.2509.09124) reports static measurements of frequency stability and linewidth narrowing under quasi-static locking, the dynamic switching performance required for laser-cooling cycles warrants its own quantitative analysis.

Version 1:

Reviewer comments:

Reviewer #1

(Remarks to the Author)
The authors addressed all the comments. I am happy for the manuscript to be published.

Open Access This Peer Review File is licensed under a Creative Commons Attribution 4.0 International License, which permits use, sharing, adaptation, distribution and reproduction in any medium or format, as long as you give appropriate credit to the original author(s) and the source, provide a link to the Creative Commons license, and indicate if changes were made.
In cases where reviewers are anonymous, credit should be given to 'Anonymous Referee' and the source.
The images or other third party material in this Peer Review File are included in the article's Creative Commons license, unless indicated otherwise in a credit line to the material. If material is not included in the article's Creative Commons license and your intended use is not permitted by statutory regulation or exceeds the permitted use, you will need to obtain permission directly from the copyright holder.
To view a copy of this license, visit https://creativecommons.org/licenses/by/4.0/

NCOMMS-26-008311: Author Response to Reviewers

Dear Nature Communications Reviewers:

We would like to thank the reviewers for their valuable time, questions, feedback, and comments. The points raised by the reviewers and their active involvement have resulted in a greatly improved manuscript as well as more effective communication of the significance of these results to a broad audience.

We have provided below a detailed response on a point-by-point basis for each reviewer's comment (*blue*) with our response (*black*) and an excerpt of the updated text (*red*).

Reviewer #1 (Remarks to the Author):

The manuscript entitled "Sub-Doppler rubidium atom cooling using a programmable agile integrated PZT-on-SiN resonator" by Andrei Isichenko et al. investigates the use of a high Q SiN ring resonator, whose resonance frequency can be dynamically tuned as a cavity to which an external DBR laser is locked to provide holdover between referencing to rubidium spectroscopy Sub-Doppler rubidium atom cooling. The experimental demonstrations are carefully described in the manuscript and most of the questions that I had were answered in the manuscript or the supplementary information. The manuscript is well structured and accessible for a broad of the photonic community. The work is of interest to the wider photonic integrated circuit and quantum sensing community as it is a step toward the size and power reduction of next generation quantum photonic technologies. As such I am supportive to publish this work in Nature Communications after addressing the following comments:

1) Was there any reason why the authors did not directly self-injection locking experiments of the laser to the PZT cavity? It feels like the consequential next step that would simplify the setup and reduce the photonic footprint significantly. Currently, it feels a bit strange to have only the piezoelectric controllable cavity on chip and everything else is fiber coupled to it. It would be good to discuss this.
(I note that it is pitched in the outlook section)

> **Response:** We thank the reviewer for their careful reading of the manuscript and for their constructive comments. We did not pursue self-injection locking (SIL) in this work due to the complexity of laser packaging (all performed at UCSB), modal mismatch between the laser chip and PIC, and compatibility with lasers used for cooling and trapping experiments at University of Wisconsin - Madison (UW-M). Both labs UCSB and UW-M have Photodigm DBR lasers and corresponding locking electronics and this made it easier to mirror our initial experiments at UCSB. Separately at UCSB we have attempted SIL with these chips; however, the modal mismatch between the edge-emitting laser and the input waveguide of the PZT PIC resulted in a limited locking range and therefore poor locking stability. We agree that fully integrated SIL operation is an important next step and have highlighted this direction in the outlook.
>
> **Revision:** Discussion, paragraph 4
>
> "Looking forward, this stress-optic tuning approach is compatible with high-bandwidth self-injection-locked (SIL) architectures (30, 25), where direct feedback between the PZT-tuned resonator and the laser can further enhance frequency stability and tuning range. In the present work, however, we employ a fiber-coupled configuration, as achieving robust SIL is currently limited by modal mismatch between the edge-emitting laser and the integrated waveguide, which

reduces feedback strength and locking stability. The PZT-actuated resonator thus represents one element of a broader set of integrated photonic components required for atomic and quantum systems."

2) Could the authors discuss the residual amplitude modulation that they get when modulating the drive current of the laser?

Response: The reviewer raises an important point regarding residual amplitude modulation (RAM) arising from current modulation of the DBR laser. In our system, RAM originates from the 1.2 MHz current modulation used to generate the frequency dither. Due to carrier density dynamics in semiconductor lasers, this modulation produces both frequency and amplitude modulation components. To minimize RAM, the modulation depth was kept in the small-signal regime. The RF drive applied to the Vescent laser current controller is attenuated (e.g., a 2.0 Vpp source followed by 42 dB attenuation, as shown in Supplementary Fig. 3) resulting in 16 mV_{pp} at the controller input. With a modulation coefficient of 1 mA/V, this corresponds to a current modulation of 16 μA_{pp}. Compared to the operating current of ~200 mA, this yields a modulation depth on the order of 10^{-4}. The modulation amplitude was adjusted while monitoring the free-running (FR) laser frequency noise and comparing the noise with and without modulation. The chosen modulation depth produces no measurable change in frequency noise or linewidth within our measurement resolution.

Revision: Supplementary Note 1, sub-section "Laser locking"

"To generate error signals for both locking stages, we apply a single 1.2-MHz modulation tone to the 780-nm distributed Bragg reflector (DBR) laser via direct current modulation. This signal is attenuated to ensure operation in the small-signal regime (16 mV_{pp} at the laser current controller input, which is 16 μA_{pp} with a 1 mA/V modulation coefficient, for a 200-mA bias current) which corresponds to a modulation depth of order 10^{-4}. The modulation depth was chosen to balance a large error signal with minimal added noise, and we confirmed this by comparing the frequency noise of the free-running laser with and without modulation."

3) Fig. 2b). It seems that the oscillations of the unbalanced Mach-Zehnder interferometer are non-period in the narrow frequency sweep. Why is this the case? I find this somewhat surprising.

Response: The apparent non-periodicity does not originate from the Mach–Zehnder interferometer itself. Rather, it is caused by the nonlinear relationship between the DBR laser drive current and optical frequency during the frequency sweep. Since the laser frequency does not change linearly with current, equal current steps correspond to unequal frequency steps, causing the MZI fringes to appear non-periodic when plotted against the raw sweep axis. To correct for this effect, we used the unbalanced fiber Mach–Zehnder interferometer as a frequency reference. The calibrated MZI free spectral range (FSR) and fringe peaks were used to determine the frequency detuning and resample the data onto a linearized frequency axis.

Revision: Figure 2 caption

"b) … The frequency detuning is calibrated using an unbalanced Mach-Zehnder interferometer (MZI, blue trace) due to laser current and frequency scan nonlinearity."

4) Fig. 2e) Can the authors add a sentence to discuss why the piezo tuning curve is quite nonlinear at low voltage levels?

Response: The nonlinear behavior at low voltage arises from the ferroelectric hysteresis of the PZT actuator. In particular, when operated near the coercive voltage range (approximately −3 V to 3 V), domain switching and polarization dynamics lead to increased nonlinearity, hysteresis, and time-dependent drift[1]. To mitigate these effects, the PZT is operated over a unipolar range (typically 3–12 V), where the response is more linear and stable. The low-voltage region shown in Fig. 2E reflects operation closer to the coercive regime, where these nonlinearities are expected.

Revision: Main text, Results, "Resonator characterization"

"The optical transmission spectrum (Fig. 2D) shows resonance tuning as a function of DC control voltage from 0 to 10 V and a resonance shift corresponding to a linear tuning strength of 1 GHz/V (Fig. 2E). Due to hysteresis in the stress-optic tuning, forward and reverse voltage sweeps yield slightly different resonance shifts, as shown in Supplementary Fig. 1b. The increased nonlinearity at low voltages is discussed further in Supplementary Note 1."

Revision: Supplementary Note 1:

"Supplementary Figure 1b shows static tuning measurements of die 3, the device used in the sub-Doppler cooling demonstration, where forward (scan-up) and reverse (scan-down) DC voltage sweeps reveal a small bias-dependent hysteresis. This hysteresis manifests primarily as an offset between the two tuning curves, while the tuning slope remains approximately linear over the full voltage range. We also measure the hysteresis tuning curve for die 6 over a larger voltage range, from negative to positive voltages, with different directions for sweep (Supplementary Fig. 1d. This behavior originates from the ferroelectric nature of the PZT, where domain-wall motion and polarization switching lead to a field-history-dependent strain response, particularly near the coercive voltage regime[5]. This results in a history-dependent mapping between applied voltage and laser frequency. During repeated actuation cycles, this effect can lead to slow variations in the cooling laser detuning, which in turn produces observable changes in magneto optical trap (MOT) fluorescence as discussed below."

5) Fig. 4 caption. The authors used c), I believe that they mean b)? Also, I find the timing information confusing. It is labeled that it was a 6 ms, but the arrow from the data point in the graph indicates 36 ms. Which one is it?

Response: Correct, it should be "b". Additional clarity could have been provided on the time of flight (TOF) duration. The TOF was indeed 6 ms, but we plot the squared time data. Squared time data (and squared cloud radius) allow a linear extraction of temperature.

Revision: Figure 4 caption. Replace "c)" with "b)" in Figure 4 caption.

Revision: Results, Sub-Doppler cooling, seventh paragraph

"…Squared TOF time values are plotted to linearize the extraction of atom cloud temperature (see Supplementary Equation S5)."

6) Supplementary information, Section 1: Can the authors discuss where the observed hysteresis is originating from?

Response: The observed hysteresis originates from the ferroelectric nature of the lead–zirconate–titanate (PZT) actuator. In particular, domain-wall motion and polarization switching in the PZT lead to a field-history-dependent response, resulting in hysteresis and nonlinearity in the tuning behavior, especially near the coercive voltage regime.[1]

Revision: Supplementary Note 1, "Small signal modulation and hysteresis", last paragraph

"This hysteresis manifests primarily as an offset between the two tuning curves, while the tuning slope remains approximately linear over the full voltage range. This behavior originates from the ferroelectric nature of the PZT, where domain-wall motion and polarization switching lead to a field-history-dependent strain response, particularly near the coercive voltage regime[5]."

7) Fig. 2S: Is the DBR laser also in the aluminum box or is it external to it? I was trying to find it, but it was not clear to me.

Response: The DBR laser is not in the aluminum box; it is external package and the light is routed via fiber which enters the PIC-package.

Revision: Supplementary information, Supplementary Figure 2a caption:

"a) Setup for laser stabilization and PZT PIC control, with the packaged PIC inside the enclosure highlighted in orange. The DBR laser (not shown) is external to the PIC package and laser light enters the PIC through an optical fiber. The package is temperature controlled and has inputs for the PZT control. The Red Pitaya FPGA is used as the servo for the 780 laser lock to the PZT PIC resonator."

Reviewer #2 (Remarks to the Author):

In this manuscript, the authors demonstrate sub-Doppler cooling of ^{87}Rb atoms in a 3-D magneto-optical trap (MOT) using a sideband-free scheme based on a fully integrated, low-power, fast-tunable optical frequency reference instead of bulk AOMs/EOMs, especially for cooling and detection beams. Their integrated, PZT-tunable Si3N4 ring resonator ($Q \simeq 2$–3×10^6) serves both as a short-term frequency reference and as a fast, low-power frequency shifter for a commercial-off-the-shelf (COTS) 780 nm laser. The laser is Pound-Drever-Hall (PDH) locked to the high-Q resonator, whose resonance is itself periodically disciplined to an 87Rb saturation-absorption-spectroscopy (SAS) feature (the F = 2 → F' =

2, 3 crossover) to ensure long-term stability. During each cooling cycle, the SAS serve is temporarily gated off, and programmable voltages on the PZT actuator shift the cavity resonance with low power ($\lesssim$10 nW)—and hence the locked laser—through the tens-of-MHz detunings needed for MOT loading, followed by a sub-Doppler polarization-gradient-cooling (PGC) ramp (15 MHz over 12 ms), while a COTS semiconductor optical amplifier (SOA) supplies intensity control. In their device characterization, the ring provides $\simeq$10 GHz of total tuning ($\approx$1 GHz/V) with an 11 MHz small-signal bandwidth. Using this sideband-free scheme—in which no bulk AOMs, EOMs, or multi-watt RF drivers are required—the authors cool atomic clouds to 16 μK and confirm atomic temperatures and atom numbers through several methods: PIC-based laser cooling for a steady-state MOT, PIC-based frequency ramp only, PIC-based frequency ramp & SOA intensity control, and conventional AOM-based frequency ramp & SOA intensity control.

This multidisciplinary work between atomic physics and integrated photonics is impressive and represents a significant step toward fully integrated, PIC-based cold-atom systems by eliminating bulky, high-power modulator chains. They showed (1) dual-stage PDH and SAS locking in a single PIC package with cycle-to-cycle repeatability; (2) atom temperature comparison and beat-note stability data, and device characterization, as well as a path shown toward a full PIC driven laser system.

I recommend accept with major revisions for publication in Nature Communications. The authors may need to present several results more quantitatively.

> **Response:** We wish to thank the reviewer for their time, attention, comments, and insight, all of which will greatly improve the quality of our manuscript.

In particular, although a companion manuscript (Ref [23], doi:10.48550/arXiv.2509.09124) reports static measurements of frequency stability and linewidth narrowing under quasi-static locking, the dynamic switching performance required for laser-cooling cycles warrants its own quantitative analysis.

> **Response:** In our prior work, "Multi-laser stabilization with an atomic-disciplined photonic integrated resonator"[2], we reported static measurements of frequency stability and linewidth narrowing using a thermally actuated ultra-high-Q resonator. In contrast, the present work focuses on a PZT-actuated high-Q resonator designed for rapid and repeatable laser frequency tuning during cold-atom experimental sequences, where agile switching performance is the primary objective rather than achieving laser linewidth narrowing.
>
> We agree that quantitative characterization of the laser behavior during dynamic actuation strengthens the manuscript. We include a fast actuation measurement of a laser PDH locked to the resonator with a step-pulse voltage input to the PZT, for two consecutive cycles. This demonstrates the laser's ability to track the resonator, showing 10-90% rise-time of 300 μs.

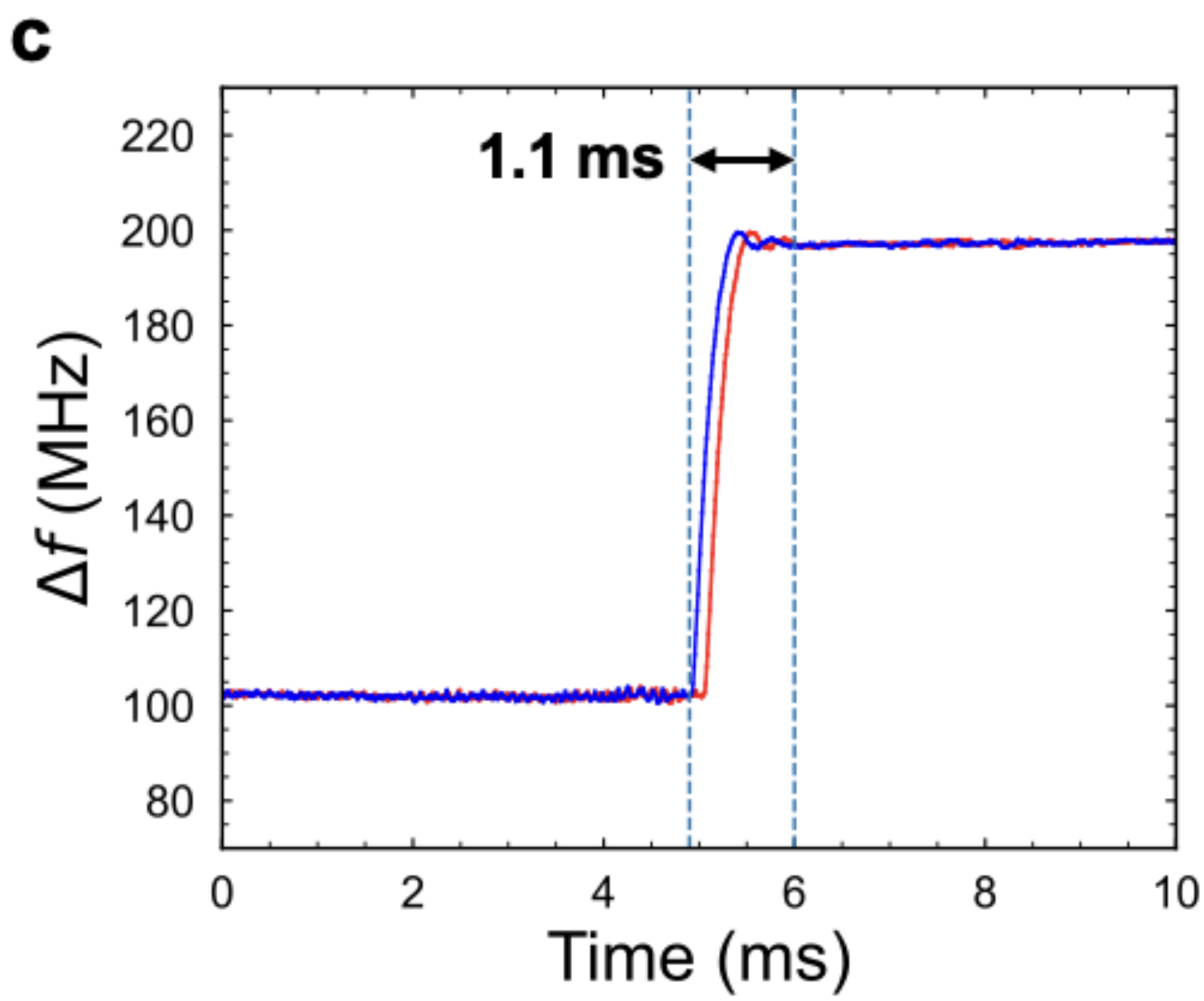

We also include measurements of the laser frequency noise. Due to the relatively low Q and extinction ratio of the PIC resonator, the PDH discriminator slope is substantially smaller than that of the ultra-high-Q resonator reported in Ref. [2], limiting the achievable frequency-noise suppression. Based on measurements of the in-loop error-signal PSD, the measured laser frequency noise, and calculations using the cavity linewidth, modulation depth, photodetector gain, and optical power incident on the detector (~50 uW), we estimate a discriminator slope in the range of 1–5 nV/Hz. The Red Pitaya servo has an output voltage noise of 1e-12 V^2/Hz [3] which corresponds to a servo-injected noise estimate of upper bound of 10^6 Hz^2/Hz. Note that we did not make modifications[4] to the Red Pitaya to reduce voltage noise and we did not high-pass filter the 1.2 MHz output. The exact values for the discriminator slope and Red Pitaya voltage noise at the time of the frequency noise measurements were not recorded. Future experiments will follow the guidelines of Ref. [5] for system discovery to achieve improved FN reduction as well as lower-noise servo. We also note that the noise reduction factor for self-injection locking can be significantly higher for a resonator of this Q.

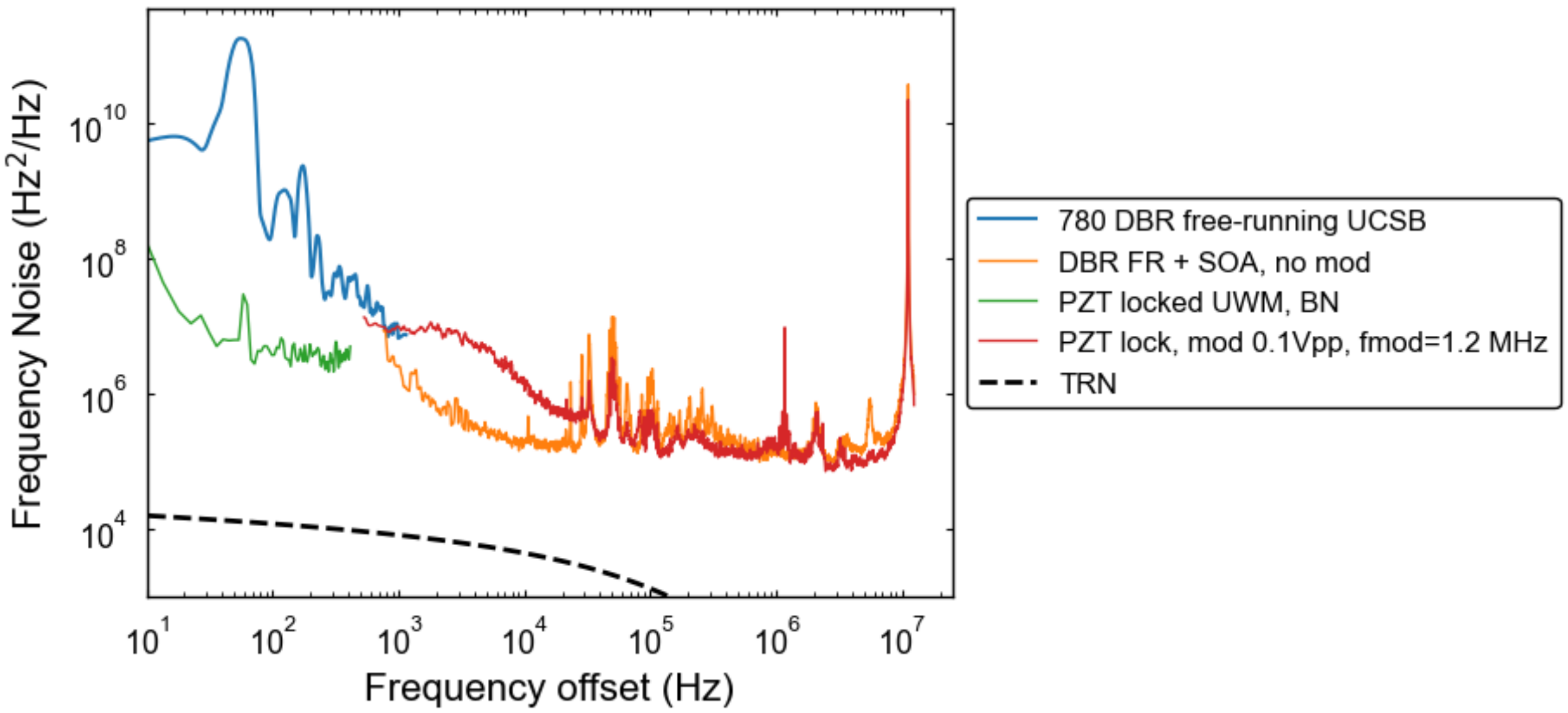

Revision: Supplementary Information, added new Supplementary Figure 4

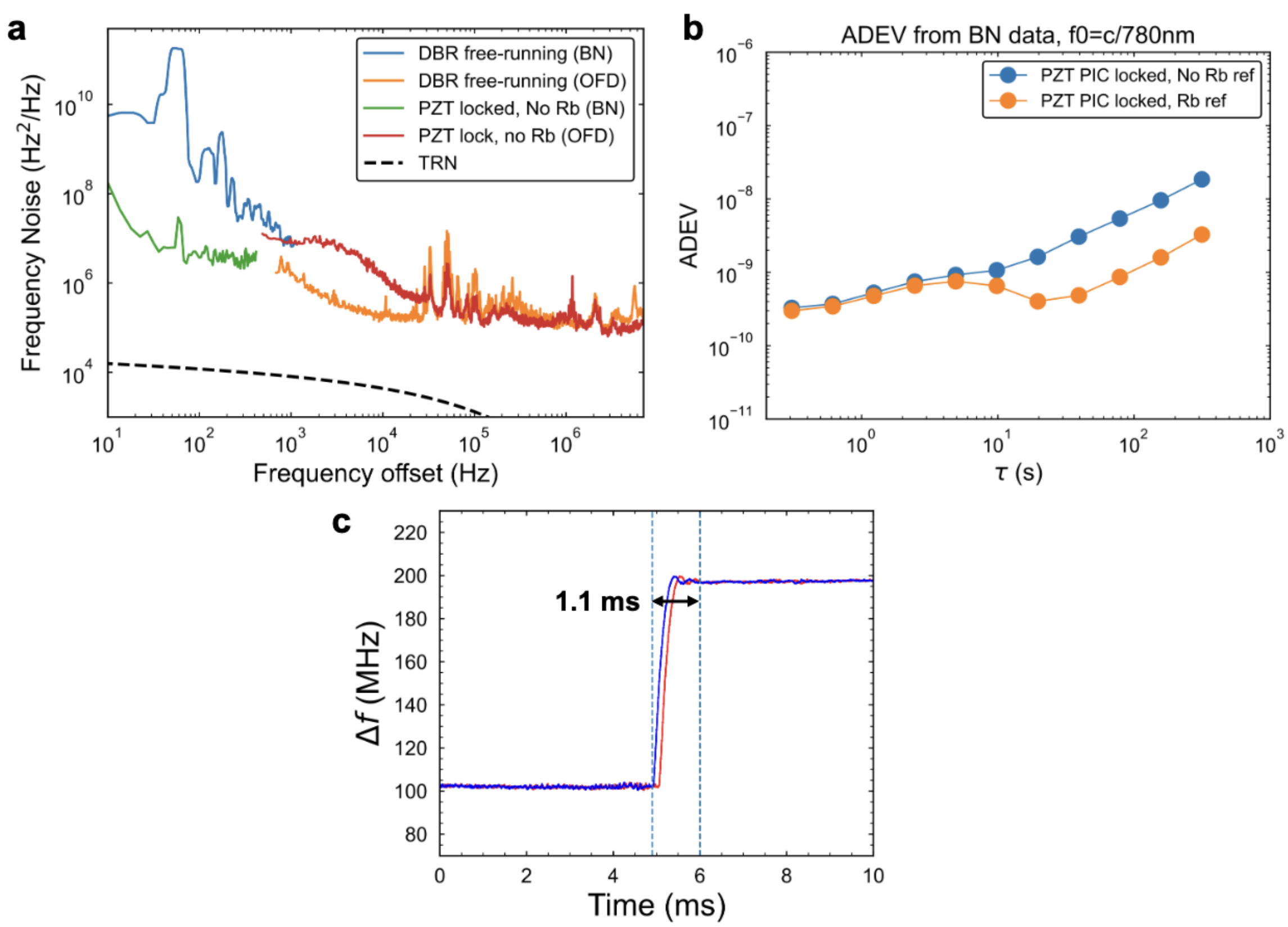

Supplementary Figure 4: Frequency-noise, stability, and agility measurements. a) Frequency-noise spectra of the free-running and PZT-locked 780 nm DBR laser measured using both an optical frequency discriminator (OFD) and a laser–laser beat-note (BN). The PZT lock suppresses low-frequency laser noise, while residual frequency noise is limited by the PDH discriminator

slope and servo noise. The dashed black curve shows the calculated thermo-refractive noise (TRN) limit of the resonator. b) Allan deviation (ADEV) calculated from the beat-note frequency time series of Fig. 5B for operation with and without Rb disciplining. The Rb-disciplined configuration exhibits improved long-term frequency stability for averaging times above approximately 4 s. c) Laser beat-note of the PZT-locked laser during a voltage step applied to the PZT actuator measured with a 10 μs frequency counter gate time. A 100 MHz frequency step exhibits a rise time of approximately 300 μs and settles to the final value in approximately 1.1 ms.

Revision: Supplementary Note 1, Laser locking, last paragraph

"The laser frequency-noise spectra measured using both an optical frequency discriminator (OFD) and an independent laser--laser beat note are shown in Supplementary Fig 4a. Locking the DBR laser to the PZT-actuated resonator suppresses low-frequency frequency noise relative to the free-running laser, while the residual frequency noise is limited by the modest PDH discriminator slope and servo noise. Based on the cavity linewidth (184 MHz), detected optical power ($\sim$50μW), and agreement between the measured in-loop and out-of-loop noise spectra, we estimate a PDH discriminator slope of approximately 1--5 nV Hz^{-1}. Supplementary Fig.4b further shows that Rb disciplining improves long-term frequency stability for averaging times above approximately 4 s. We further note that the Rb-disciplined ADEV does not exhibit ideal $\tau^{1/2}$ scaling because the resonator is only loosely disciplined to the atomic reference. The finite spectroscopy discriminator slope, residual servo error, and environmental perturbations limit the transfer of atomic-reference stability to the resonator, resulting in Allan deviation behavior that differs from that expected for purely white-frequency noise. In addition to frequency stability, rapid and repeatable frequency actuation is required for laser-cooling sequences. Supplementary Fig.4c therefore characterizes the dynamic response of the PZT actuator. A 100-MHz programmed frequency step exhibits a 10-90% rise time of approximately 300 μs and settles within 1.1 ms, confirming the agility required for sub-Doppler cooling sequences."

General Comments:
1. Please provide an energy level diagram showing the relevant laser transitions for a general audience.

Response: We have produced an additional figure for the Supplementary Materials which shows the relevant laser transitions and the D1 and D2 ^{87}Rb structure. This figure will be referenced in the main text. We also updated the main text Figure 1 to include a panel with a simplified version of this energy level diagram.

Revision: Main text, "Sub-Doppler atom cooling", paragraph 5

"The 780-nm laser drives a red-detuned ^{87}Rb $5S_{1/2}\, F = 2 \rightarrow 5P_{3/2}\, F' = 3$ transition, while a separate 795-nm DBR laser acts as the repump, addressing the ^{87}Rb $5S_{1/2}\, F = 1 \rightarrow 5P_{1/2}\, F' = 2$ transition (see Supplementary Fig. 5)"

Revision: Supplementary Information, "MOT system and characterization and measurement"

"...The relevant energy level transitions are shown in Supplementary Fig. 5."

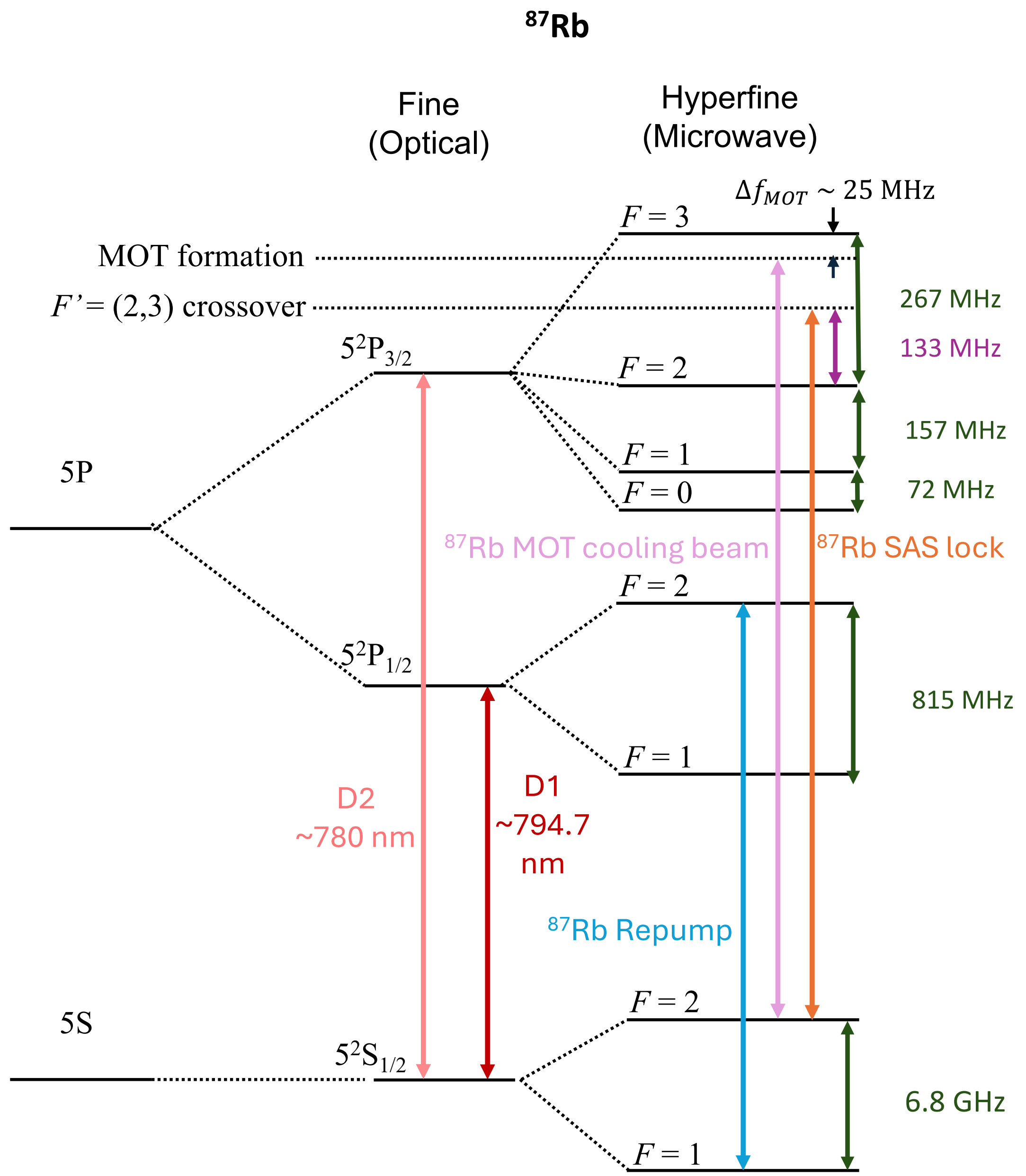

"**Supplementary Fig. 5: Energy level diagram.** Energy level diagram of ^{87}Rb including the D1 (light red) and D2 (dark red) transitions and the hyperfine structure. Relevant experimental laser frequencies are color coded: the 795-nm repump beam (light blue), and the 780-nm MOT cooling beam (pink, red-detuned by 25 MHz) and the ^{87}Rb saturation absorption spectroscopy (SAS) lock frequency (orange). During the MOT formation, the cooling laser rapidly switches between the green and orange frequency settings by means of the PZT-actuated resonator."

Revision: Main text, Figure 1

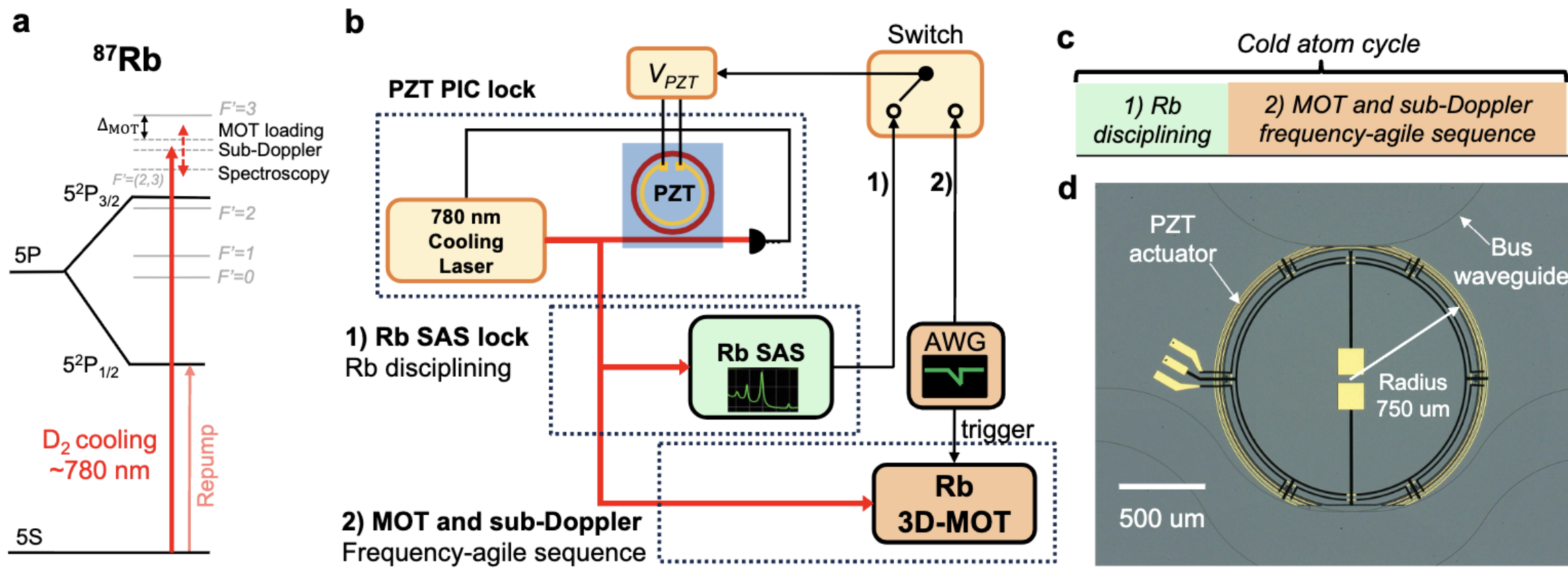

"…**a)** Simplified ^{87}Rb energy-level diagram showing the cooling and repump transitions and the rapid PZT-controlled detuning of the 780-nm cooling laser used to transition between magneto-optical trap (MOT) loading, sub-Doppler cooling, and spectroscopy referencing…"

Revision: Main text, Results, first paragraph

"The PZT-on-SiN resonator provides an integrated platform for locked laser frequency control, combining short-term stability with fast tuning for programmable control and long-term atomic referencing. In laser-cooled ^{87}Rb systems, rapid frequency tuning of the cooling laser is required to transition from magneto-optical trap (MOT) loading to sub-Doppler cooling, as illustrated in Fig. 1a. The working principle of the PZT-controlled agile resonator cavity used to implement this functionality is shown in Fig.1b–d."

2. Please describe coherently which parts of your laser system are COTS (e.g. repump and SOA lasers) and which are custom PIC-based subcomponents (e.g. the PZT-tuned resonator).

Response: We agree with the reviewer and have appended a paragraph outlining the architecture, specifically stating which components are commercially available and which are custom.

Revision: Main text, Results, first paragraph

"...anchoring the PIC-locked laser to an atomic transition and reducing the long-term laser frequency drift. Our laser system combines commercial off-the-shelf (COTS) components with a custom PIC. The COTS components include the cooling and repump DBR lasers, a fiber-coupled SOA for optical power modulation and shuttering, and the free-space ^{87}Rb SAS setup. Combined with SOA-based power modulation, the PIC-based frequency control removes the need for external acousto-optic or electro-optic modulators (AOMs/EOMs) for sub-Doppler cooling."

3. To illustrate the Doppler-cooling and sub-Doppler PGC processes,

o Please explain coherently the cooling and repump beams:
(1) the optical transitions of the required lasers and how each laser is locked to its corresponding atomic transition; and

Response: We have added a detailed energy level diagram at Supplementary Figure 5 in the Supplementary information where the frequencies of the lasers are denoted. Please see the text additions below. Additionally, we have included a more simplified energy level diagram in the first figure of the paper:

Revision: Figure 1 updated:

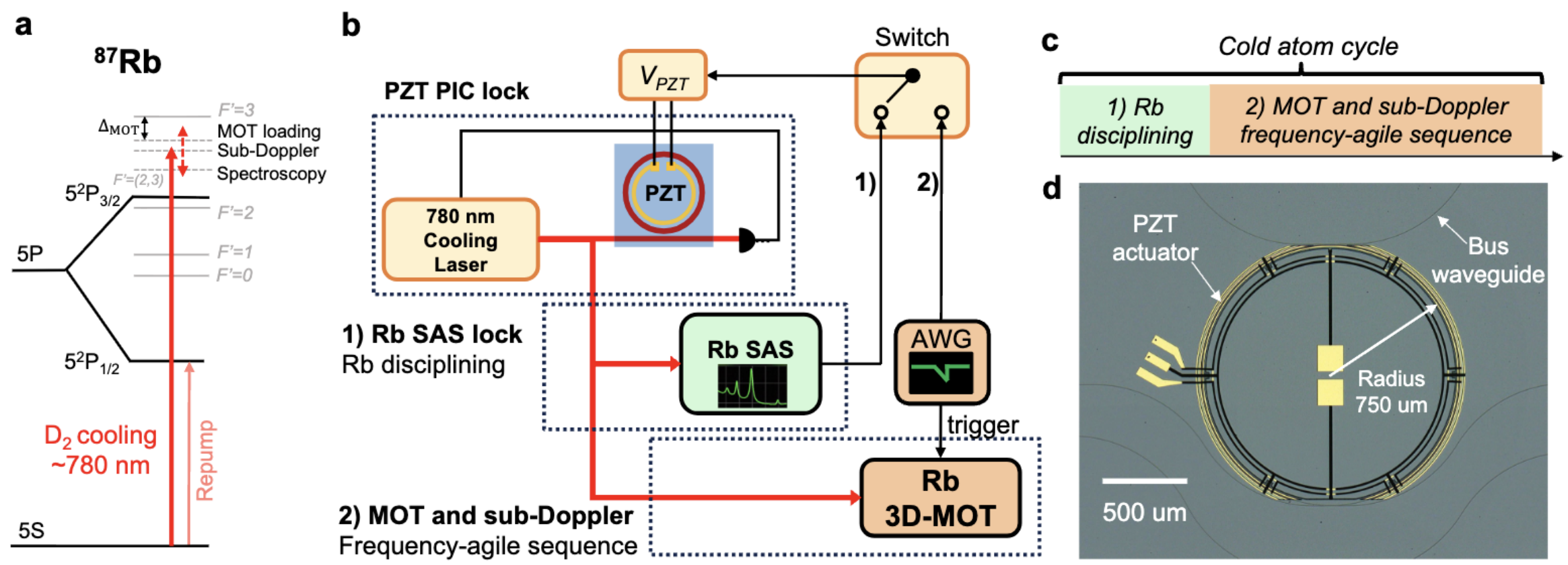

Revision: Main text, "Sub-Doppler atom cooling", paragraph five:

"The 780-nm laser drives a red-detuned ^{87}Rb $5S_{1/2}$ $F = 2 \longrightarrow 5P_{3/2}$ $F' = 3$ transition, while a separate 795-nm DBR laser acts as the repump, addressing the ^{87}Rb $5S_{1/2}$ $F = 1 \rightarrow 5P_{1/2}$ $F' = 2$ transition (see Supplementary Fig. 5).

Revision: In Supplementary Information, "Laser locking", first paragraph:

"The PZT-on-SiN resonator device is packaged and integrated into the experimental system as shown in Supplementary Figure 2. The packaged photonic integrated circuit (PIC) is mounted on a temperature-controlled stage and wirebonded to provide electrical access to both the PZT actuator and integrated thermal tuners (Supplementary Fig. 2b,c). Laser stabilization is implemented using a Pound–Drever–Hall (PDH) locking scheme and the schematic of the full laser stabilization architecture is shown in Supplementary Figure 3a. The 795-nm repump laser is stabilized to ^{87}Rb saturation absorption spectroscopy (SAS) via a Red Pitaya field-programmable gate array (FPGA) platform running the open-source Linien laser-locking software[7], which modulates the laser current through a Vescent D2-105 laser controller. The 780-nm cooling laser incorporates a dual-stage laser-to-PIC and PIC-to-SAS locking scheme. The laser-to-PIC lock employs a second Red Pitaya FPGA, which receives a photodiode signal from the output fiber of the PIC resonator and stabilizes the laser to the transmission minimum of the PIC resonance. During operation, the PIC-to-SAS lock provides periodic referencing of the PIC resonator to a second stable ^{87}Rb SAS setup. This lock employs another Vescent D2-105 laser controller and a Vescent D2-125 laser servo. The PIC-to-SAS lock is routinely unlocked and relocked during the

experiment to allow the laser frequency to be rapidly driven to different values by shifting the PIC cavity resonance via the PZT element."

(2) the schemes for frequency control and intensity control & stabilization, as well as the optical-switching mechanism (including extinction ratio).

Response: Frequency control of the cooling laser is provided entirely by the laser current feedback based on the laser tracking the PZT PIC resonance. Intensity stabilization was not used in this experiment, i.e. we did not use an intensity servo for any of the beams going to the MOT, for the optical paths going into the spectroscopy, or for the PZT PIC package.

We use the Thorlabs BOA780P semiconductor optical amplifier (SOA) for the optical shuttering in time-of-flight measurements and in the optical power control in sub-Doppler PGC. The shuttering is performed via modulation of the injection current (i.e., the forward bias of the diode). When the injection current is reduced to near zero, the device no longer provides gain and behaves as a low-transmission (absorptive) waveguide. Using this method, we measure an optical on/off ratio (rather than a traditional extinction ratio) of 55 dB (34.7 mW on, 0.100 µW off), where the off-state power includes residual leakage, ASE, and background contributions. This switching method, while slower than an AOM, is significantly faster than the millisecond timescales of our experiment. We have updated the text accordingly.

See an optical shuttering test on this SOA:

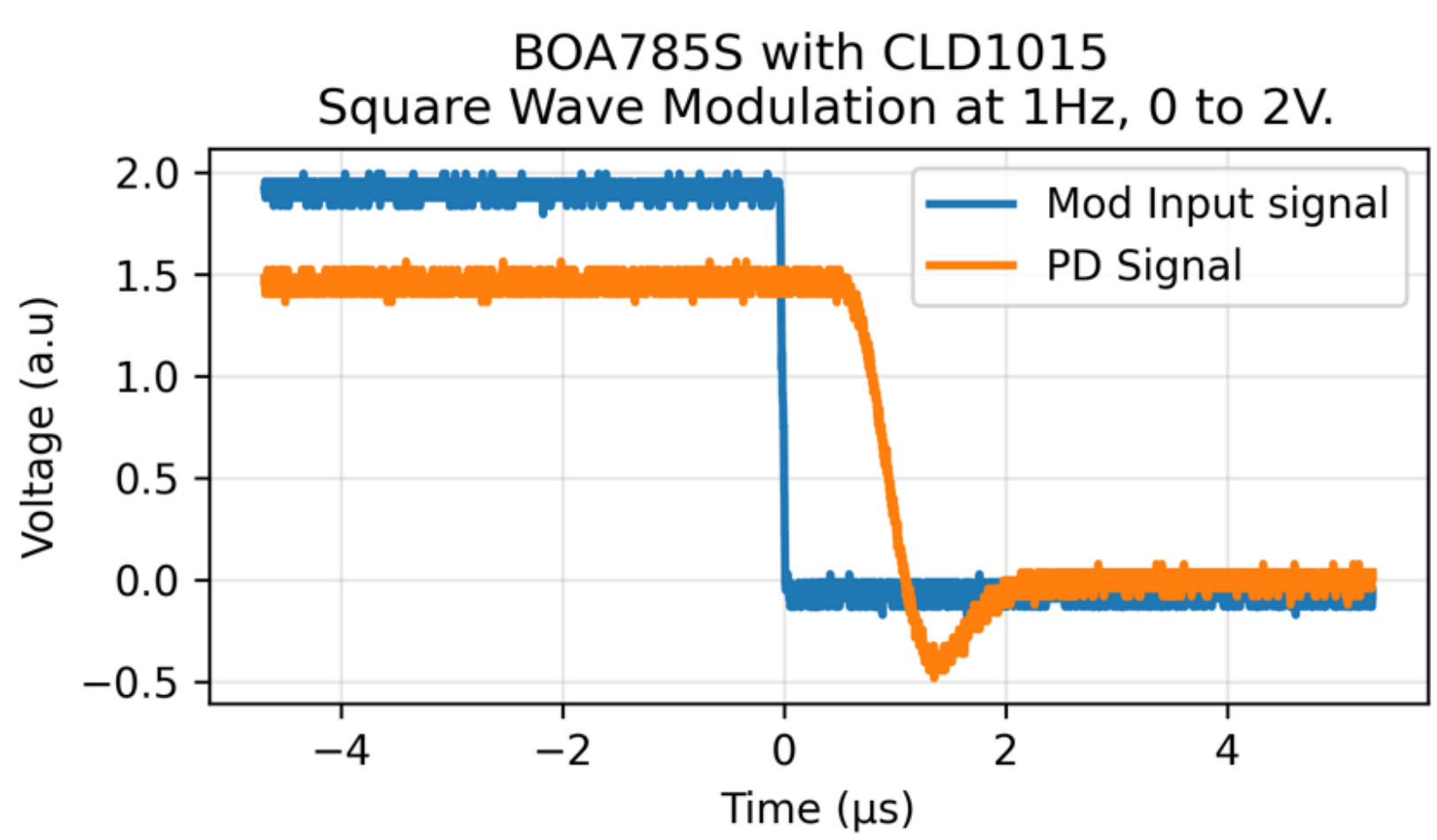

Revision: Supplementary information, Supplementary Figure 7, caption:

"All time-of-flight (TOF) measurements are performed within a repeating 305 ms control sequence, with the TOF delay τ_{TOF} varied between runs. SAS re-referencing occurs during two 25-ms windows at the beginning and end of each cycle when the relay-jump transistor-transistor logic (TTL) pulse is low. MOT loading begins at τ_{seq} = 10 ms and continues until 238 ms for sequences including PGC, or until 250 ms when PGC is omitted. During PGC, the magnetic field is switched

off at 238 ms (250 ms without PGC) and remains off until 275 ms. At τ_{seq}=250 ms, the cooling and repump beams are shuttered via gain switching of the SOA (current modulation), achieving an optical on/off ratio of 55 dB, and the atomic cloud subsequently undergoes ballistic expansion for a variable τ_{TOF}. Imaging is performed using a 0.5-ms camera exposure, after which the laser to the cold atoms is optically shuttered and is returned to the SAS reference point to prepare for the next cycle."

o Please explain the configuration of the atom-detection/fluorescence beam used to measure atom temperature and atom number.

Response: We agree that these explanations should be presented clearly and in more detail. We have added the text in red below to address these details.

Revision: Main text, "Sub-Doppler atom cooling"

"A 0.5 ms illumination pulse, applied at Δ_{MOT} (and optionally at $\Delta = 0$ in future runs), captures the atomic fluorescence image. The illumination pulse is generated by the cooling laser, which is shuttered from the atoms via an SOA during the time of flight (TOF). During this period, the laser is set to the MOT formation (red-detuned) frequency and subsequently flashed onto the atom cloud via SOA control. At t_{seq} = 290 ms, the system re-engages ^{87}Rb referencing by first returning the PZT to the lock point and then re-enabling the SAS feedback."

To address the second point, the following text is modified in the main text, "Sub-Doppler atom cooling" section:
"After this ramp, all MOT and repump light is switched off at t_{seq}=250 ms, initiating a TOF measurement with delays ranging from 0 to 6 ms. A 0.5 ms illumination pulse, applied at Δ_{MOT} (and optionally at $\Delta = 0$ in future runs), captures the atomic fluorescence image. The illumination pulse is generated by the cooling laser, which is shuttered from the atoms via an SOA during the time of flight (TOF). During this period, the laser is set to the MOT formation (red-detuned) frequency and subsequently flashed onto the atom cloud via SOA control. At t_{seq} = 290 ms, the system re-engages ^{87}Rb referencing by first returning the PZT to the lock point and then re-enabling the SAS feedback."

4. Please add error bars on your key data plots (e.g. atom temperature and atom number measurements).

Response: We have added error bars to our atom temperature measurement plot (main text, Figure 4). We include Gaussian-fit uncertainty in σ^2 and incorporate this error in the extraction of temperature via the linear fit. We also include uncertainty in the imaging calibration (μm per pixel). Additionally, we recomputed our fits to also extract σ_0, rather than basing the fit on the first measured time-of-flight. Details of the error bars have been added to the Supplementary Materials.

For the calculation of uncertainty with atom number, other uncertainties have been added to Supplementary Table 1 including for Ω and η_{geo}. The effects of uncertainty in camera gain or quantum efficiency are not included in our analysis. Atom number uncertainty has been computed

via Monte Carlo propagation. Information on this calculation has been added to the Supplementary Materials.

Revision: Main text, Figure 4. Updated with error bars.

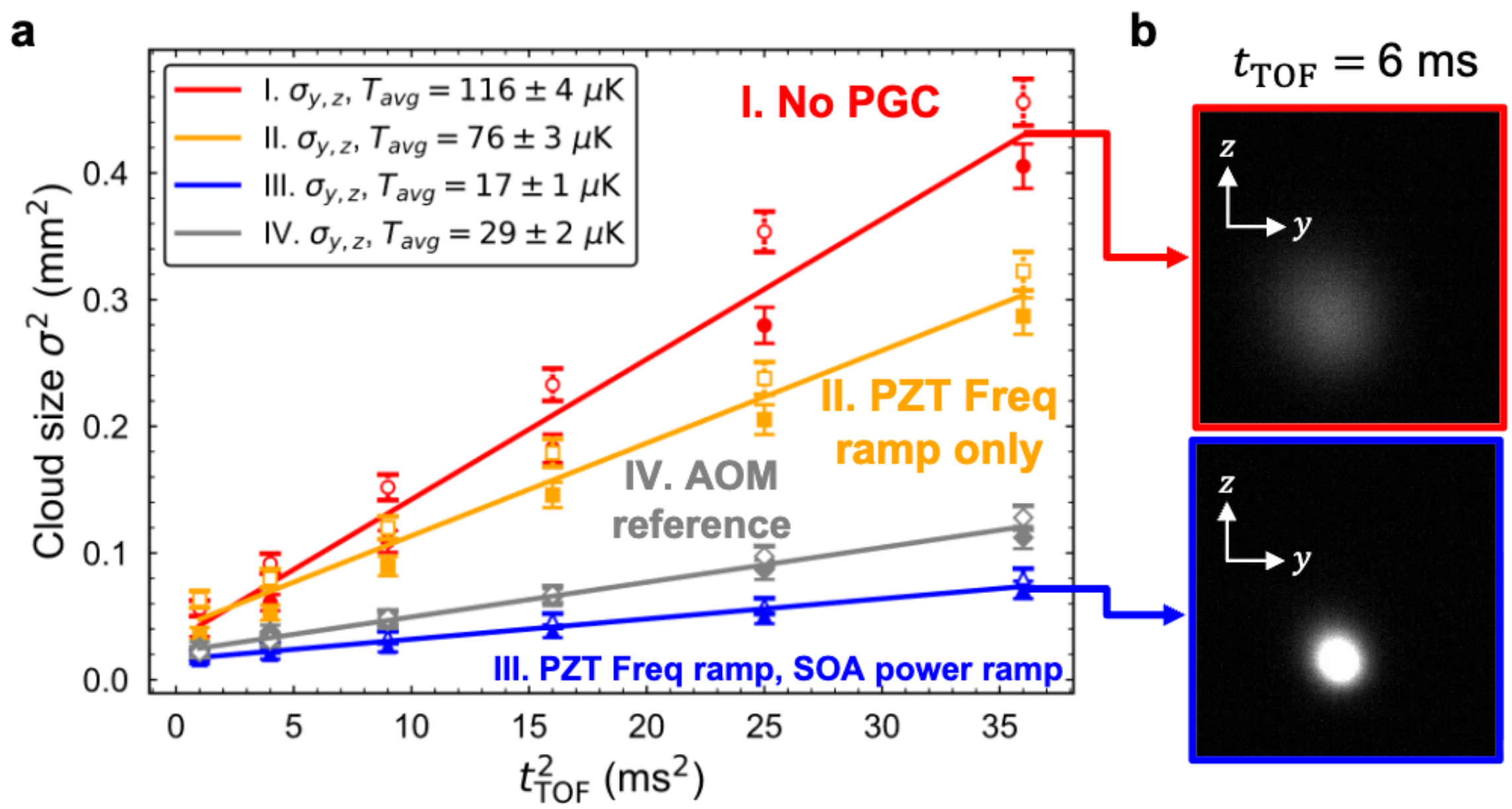

Figure 4 caption:

"Each datapoint is extracted from a 10-shot accumulated fluorescence image (each with a 0.5 ms exposure). Vertical error bars represent the propagated Gaussian-fit uncertainty in σ^2, including a one-pixel error floor. The quoted temperature uncertainties include both the fit/extraction uncertainty and the systematic uncertainty from the imaging calibration added in quadrature."

Revision: Supplementary information, temperature uncertainty calculation

"The procedure was performed independently for each of the two image axes, yielding fitted widths σ_y and σ_z. The covariance matrix from the Gaussian fit was used to estimate the statistical fitting uncertainty in each extracted width. Specifically, the one-standard-deviation uncertainty in the fitted width was taken as the square root of the covariance matrix element Cov(σ, σ). To avoid underestimating the uncertainty associated with fitting a smooth accumulated image, a conservative one-pixel extraction floor was added in quadrature to the Gaussian fit uncertainty.

The image scale was calibrated using a USAG resolution target, yielding a 12.86 µm/pixel. Based on a spread among measurements of multiple features with manual extraction, a conservative 2% uncertainty was assigned to the calibration. Because the extracted temperature scales as the square of the length calibration, this contributes a 4% systematic uncertainty to the atom temperature. This calibration uncertainty is not included in the individual data-point error bars, since it is common to all measurements and therefore systematic. It is, however, included in the final reported temperature uncertainty according to

$\delta T\_tot = \sqrt{(\delta T\_fit^2 + \delta T\_cal^2)}$

Revision: Supplementary information, atom number uncertainty calculation

"Atom number uncertainty was computed via Monte Carlo propagation. Each experimentally uncertain parameter (camera gain and quantum efficiency were neglected) is sampled from independent Gaussian distributions centered on the measured value and with standard deviation given by estimated uncertainty. The full atom number is repeatedly computed from the sampled parameter set. Reported atom number is taken as the mean of this distribution, and the associated uncertainty is taken as its standard deviation. This approach avoids linearization assumptions which perform poorly in the highly nonlinear scattering expression.

Atom number uncertainty (20.3% in total) was dominated by systematic error in imaging calibration with slight contribution from shot-to-shot uncertainty in imaging light intensity and detuning."

Revision: Main text, Figure 5 caption:

"Note that atom number uncertainty (calculated via Monte Carlo propagation, see Supplementary Note 2: Atom number calculation and Supplementary Table 1) is dominated by systematic error in imaging calibration and is ~20%. This constant errorbar is neglected from the plot for clarity."

Revision: Supplementary information, Supplementary Figure 8 caption:

"a): Note that the atom number uncertainty (calculated via Monte Carlo propagation, see Supplementary Materials section "Atom number calculation") is dominated by systematic error in imaging calibration and is 20.3%. This constant errorbar is neglected from the plot for clarity."
b): "Atom number uncertainty is again 20.3% which is dominated by systematic error in imaging system calibration. The constant error bar is again neglected from the plot for clarity."

5. Please coherently explain the pro and cons of this sideband-free scheme for next-generation, low-SWaP laser system architectures.
o This approach may enable multiple beams (for cooling, detection, and state preparation) at different frequencies from the same laser in a time-sequenced cycle.

Response: Yes, this approach may simplify and lower the SWaP of quantum systems by replacing multiple GHz-spaced optical frequencies, which are typically generated using combinations of AOMs and EOMs, with a single integrated frequency reference capable of rapid resonance tuning during different temporal stages of an experimental sequence.

The primary advantages of this approach are reduced electrical power consumption, reduced system size and complexity, and improved scalability through CMOS-compatible photonic integration. In contrast to conventional RF-driven modulation approaches, the demonstrated stress-optic tuning mechanism operates with nanowatt-scale static electrical power consumption.

Furthermore, integrated resonator-based tuning may be advantageous in applications where parasitic optical frequency components are undesirable. For example, EOM-generated sidebands can drive unwanted transitions in Raman or other multilevel atomic systems, leading to parasitic couplings and degraded control fidelity.

Potential disadvantages of this approach compared to conventional AOM/EOM architectures include lower modulation bandwidth, a more limited accessible tuning range set by the resonator design, and the requirement that frequency shifts be implemented relative to optical cavity resonances. Although these resonances can be engineered for specific operating frequencies, conventional AOMs and EOMs may remain preferable for applications requiring very large frequency shifts, broadband tunability, or high-speed pulse modulation. We include a summarized version of this in the discussion section of the main text.

Revision: Main text, Discussion, paragraph 3:

"...This highlights the cavity's intrinsic ability to serve as a short-term holdover cavity, which itself arises from the excellent short-term coherence provided by the high-Q SiN cavity and its low-power PZT actuation. This functionality eliminates the need for bulk optic reference cavities and further enables integration, compactness, and low-power operation. Experiments requiring multiple GHz-spaced optical frequencies used asynchronously throughout an experimental sequence, typically generated using multiple spectroscopy-referenced lasers and combinations of AOMs and EOMs, may instead be enabled by a single rapidly tunable integrated frequency reference. In addition, resonator-based tuning may be advantageous in applications where parasitic optical frequency components are undesirable, such as Raman-based atomic control schemes. However, conventional AOM and EOM architectures may still remain advantageous for applications requiring very large frequency shifts, broadband operation, or higher modulation bandwidths."

o Sequence: first, the laser is locked to the resonator at frequency 1 (Rb discipline); next, the output is shut off via an optical switch, and the laser is tuned to frequency 2 (no Rb discipline) to produce a second beam; then the switch is reopened to resume output; next, the output is shut off again, the laser is tuned to frequency 3 (Rb discipline), and finally, the optical switch is reopened to resume the output.

Response: The reviewer's description of the sequence is correct, and we thank the reviewer for the suggestion to detail the Rb-disciplining procedure and justify timing choices (see response below).

o The authors may need to detail (1) the Rb-disciplining procedure—for example, why a 25-ms interval is required and whether it allows sufficient time for frequency scanning and relocking to the atomic transition (SAS locking),

Response: An explanation of the 25-ms disciplining period is added to the text (see below).

Frequency scanning is unnecessary as the system demonstrates reliable relocking to SAS error signal simply by driving to the starting voltage setting. ^{87}Rb-disciplining in this manner has been successfully employed in up to 30 second intervals without issue.

Subsequent work has shown that we can reduce this re-referencing interval down to 4 ms without loss of relocking robustness. Any shorter time interval and the relocking will occasionally miss (likely due to piezoelectric creep settling times).

Revision: Main text, "Results":

"Each sequence begins with a 25-ms ^{87}Rb referencing interval followed by the MOT stage and concludes with a 12-ms triangular frequency ramp corresponding to the polarization-gradient cooling (PGC) sequence. The 25-ms ^{87}Rb referencing interval used for this data collection was an overly generous choice; it was selected to ensure re-locking stability. However, this re-referencing time has since been reduced to a 4-ms interval without additional control complexity."

(2) the allowable number of Rb-disciplining applications for a given cycle time (from short to long),

Response: We can select the rate of Rb-disciplining applications with a wide range of freedom. We have demonstrated disciplining rates of 30mHz up to 100 Hz. The downside of long intervals between referencing is the accumulation of (cavity suppressed) laser frequency drifts. A sentence is added to address this.

Revision: Main text, "Resonator agility measurements", third paragraph

"…This confirms that the hybrid PDH with SAS scheme provides both short-term stability from the cavity and long-term drift suppression from the atomic reference. The re-referencing period can be selected according to the short-term frequency-stability requirements of the experiment. We have demonstrated re-referencing rates from 30 mHz to 100 Hz, including stable relocking after a 30 s MOT formation period in which the spectroscopy reference was applied only once per cycle. This further demonstrates the long-term drift suppression provided by the cavity."

(3) and any delays or dead times (e.g. in scan and relocking modes) within or between Rb-disciplining applications.

Response: As mentioned above, there is no scanning prior to this relocking. The entire process (including driving the PZT to spectroscopy, re-referencing SAS, and driving back to cooling frequency) has been completed within a single 10-ms window during which time the atoms are released for a time-of-flight temperature measurement. This allows the re-referencing to take place in an atom interferometer setup without additional deadtime.

o Frequency jumps via the PZT-tuned resonator from tens of MHz are acceptable, but larger jumps more than hundreds of MHz can destabilize the lasing condition and cause mode hopping. Please address this issue quantitatively.

Response: The DBR lasers we use in this experiment have mode-hop free regions in the tens of GHz, which greatly exceed the hundreds of MHz shifts we demonstrate in this work.

Revision: Main text, Results, first paragraph

"A commercial single-frequency 780-nm distributed Bragg reflector (DBR) semiconductor laser (Photodigm) is PDH locked to the resonator (Fig. 1b) near the middle of the 20 GHz laser mode-hop-free region."

o Lasing conditions can be affected by frequency jump and frequency scanning. Does the instantaneous laser linewidth differ in switched and static configurations?

Response: The instantaneous linewidth, often referred to as the fundamental linewidth, was not directly measured during PZT actuation. However, the frequency content of the control signal applied during tuning is limited by the feedback-loop bandwidth, which is estimated to be 10–100 kHz. In contrast, the instantaneous linewidth is typically determined by frequency-noise components at Fourier frequencies near 1 MHz and above. Therefore, the PZT control signal is not expected to directly influence the instantaneous linewidth.

Large frequency jumps can alter the laser injection current and consequently the intracavity optical power. In our experiment, a 100 MHz frequency step corresponds to a current change of approximately 0.35 mA, based on the measured laser tuning coefficient of 280 MHz/V and a laser-controller modulation coefficient of 1 mA/V. Using the Photodigm DBR laser slope efficiency of 0.97, this current variation corresponds to an optical power change of approximately 0.3 mW. For a nominal output power of approximately 140 mW, this represents a 0.2% power variation.

Since the fundamental linewidth scales approximately inversely with optical power (LW ~ 1/P), the corresponding change in instantaneous linewidth is also approximately 0.2%. The instantaneous linewidth of the Photodigm DBR laser at our operating current is approximately 330 kHz; therefore, the expected linewidth change during a 100 MHz frequency step is less than 1 kHz. We therefore do not expect a significant difference in instantaneous linewidth between the static and dynamically switched operating conditions investigated in this work.

6. Please describe in detail how on-chip power control and optical switching will be implemented in your approach. Specifically:

o Do you plan to replace the COTS SOA with a fully integrated, PIC-based SOA or with a PZT-tuned resonator for optical power ramping and switching in PGC or other sequences?

Response: On-chip power control and switching can be implemented using a critically coupled resonator, add/drop resonator, or a long-delay Mach-Zehnder interferometer[6]. This PZT-tuned "shutter" resonator can achieve an extinction ratio of over 10 dB[6,7]. By identifying the offset voltage for the resonator to keep the resonance near the laser frequency and moving the resonance onto and off of the laser frequency. This offset can be achieved with a combination of a static voltage offset for the PZT as well as the thermal tuner. Importantly, to shutter both cooling and repump frequencies, we can either design the resonator to have a lower FSR (6.8 GHz) to match

the frequency spacing, or use a pair of resonators each designed to be used with each laser frequency.

By mapping the resonator transmission transfer function to the control, the output power waveform can be tuned to achieve a square wave shutoff or a programmable shutoff waveform such as the one used for the PGC sequence.

The shutter rings will drift over time. By incorporating an output monitor tap after the shutters, we can engage a side-of-fringe lock with PID control to effectively act as an intensity servo and to keep the resonance in a known location, provided that the incident power onto the rings is power stable.

Revision: Main text, Discussion, paragraph 5 (citations numbers are as given by the revised manuscript, not the references in this document):

"We aim to implement power ramping and shuttering through critically coupled resonators driven by PZT amplitude control, while fluorescence feedback from the MOT provides absolute frequency referencing. We can achieve >10 dB extinction ratio[19,22] via shifting this resonance onto and off of the laser frequency. Recent demonstrations of PZT-actuated photonic devices have achieved optical shuttering with a 1.7 μs (90–10%) switching time and 21.5 dB extinction ratio[22]. Square wave shutoff or programmable power sequences may be implemented via mapping the resonance transmission transfer function in the control hardware. Additionally, we are developing an FPGA-based controller for complex waveform generation, data processing, and feedback control to increase frequency precision and agility[35–37]. This system concept represents a path toward a chip-scale cold-atom instrument where laser frequency, power, and atomic readout are all governed by the same photonic platform."

o What switching times and extinction ratios do you expect?

Response: The switching speed and extinction ratio will depend on the specific PZT-actuated photonic element used for amplitude control. In recent measurements[6] of a PZT-actuated coil Mach–Zehnder interferometer (MZI), we demonstrated a 90–10% optical switching time of 1.7 µs and an extinction ratio of 21.5 dB. In a separate PZT-actuated 780 nm add-drop ring resonator, we measured a resonance linewidth of 539 MHz and a small-signal f180deg frequency of 9 MHz, corresponding to sub-microsecond actuation capability. The 780-nm device exhibited an extinction ratio of approximately 12 dB. Based on these results, we expect integrated PZT-based shuttering elements to provide microsecond-scale switching times and extinction ratios in the range of approximately 10–20 dB and switching times on the order of microseconds.

Revision: Main text, Discussion, paragraph 5

"We aim to implement power ramping and shuttering through critically coupled resonators driven by PZT amplitude control, while fluorescence feedback from the MOT provides absolute frequency referencing. We can achieve >10 dB extinction ratio[19,22] via shifting this resonance onto and off of the laser frequency. Recent demonstrations of PZT-actuated photonic devices have achieved optical shuttering with a 1.7 μs (90–10%) switching time and 21.5 dB extinction ratio[22].

Square wave shutoff or programmable power sequences may be implemented via mapping the resonance transmission transfer function in the control hardware. Additionally, we are developing an FPGA-based controller for complex waveform generation, data processing, and feedback control to increase frequency precision and agility[35–37]. This system concept represents a path toward a chip-scale cold-atom instrument where laser frequency, power, and atomic readout are all governed by the same photonic platform."

Combined with integrated electronics for data processing and feedback (e.g., field programmable gate array (FPGA)-based control), this system concept represents a path toward a chip-scale cold-atom instrument where laser frequency, power, and atomic readout are all governed by the same photonic platform."

7. Please explain the advantages and limitations of this approach under harsh environmental conditions, such as thermal cycling and vibration. For example:
o The thermoelectric cooler (TEC) used to stabilize the PZT-tuned resonator can suppress moderate temperature fluctuations, but its performance may be insufficient during large ambient temperature swings or when the thermal mass heats up.
o A micro resonator is more robust than a bulk cavity, yet it remains susceptible to temperature- and vibration-induced resonance shifts.

Response: We agree that robustness under thermal cycling and vibration is important and clarify how our approach handles these conditions below.

First, locking the cavity resonance to an atomic transition is a more refined form of stabilization than holding the temperature constant, because we actuate the resonance directly against an absolute atomic reference rather than relying on passive thermal control. Several actuators maintain this lock across different timescales: the TEC provides slow, coarse stabilization of the chip temperature, the PZT tunes the resonance, and a separate thermal tuner corrects fast, kHz-rate temperature-induced shifts (although it is unused in this work). The TEC's role is therefore not to suppress all ambient drift on its own, but to keep the PZT control signal within its usable range and out of the hysteresis regime; the atomic lock and fast thermal tuner compensate for the residual temperature excursions that the TEC alone cannot follow.

For robustness against device failure, the resonators are fabricated at the wafer scale and can be densely integrated, providing redundancy.

To reduce sensitivity to vibration and large ambient swings, the enclosure of the package can be enclosed in a larger package with a resistive heater and can include a layer of Teflon for greater vibration isolation. See for example Fig. S2 in N. Chauhan et al. (https://doi.org/10.1038/s41467-026-69948-2) ref. [8].

We thank the reviewer for this comment and add the following line to our "Device fabrication and packaging section:

Revision: main text, "Device fabrication and packaging", new paragraph:

"The compact, integrated package can facilitate (via a second layer of packaging) additional thermal insulation and vibrational isolation to further mitigate the effects of temperature swings and vibrations if employed in harsh environments which could not be compensated by an aluminum package and TEC temperature control alone."

8. Please quantify the frequency stability of the 780-nm laser in your approach. While Figs. 5 and S8 qualitatively demonstrate the stability and repeatability of the tuned laser frequency, a more convincing plot would show the switched-frequency, Allan deviation (ADEV) and frequency noise (Hz^2/Hz), both with and without the Rb disciplining.

Response: We agree that quantitative frequency-stability metrics strengthen the manuscript. In the revised manuscript, we have added both Allan deviation (ADEV) and frequency-noise PSD measurements derived from the laser–laser beat-note frequency records acquired with and without Rb disciplining. Note that in the "No Rb ref" case, the same programmed frequency jumps are applied, but the Rb feedback correction is disabled. These results confirm that the Rb-disciplined configuration improves the long-term frequency stability for averaging times above approximately 4 s.

The ADEV is plotted only for averaging times $\tau > 0.3$ s. At shorter averaging times, the frequency-counter record is affected by the intentionally applied agile frequency sequence and the data processing used to isolate the quasi-static monitoring intervals. This produces a nonphysical discontinuity in the Allan deviation near the transition between the raw counter sampling interval and the down-sampled monitoring record. Since the purpose of this analysis is to quantify long-term switched-frequency stability and drift, rather than the sub-cycle dynamics of the frequency jump, we restrict the plotted ADEV to averaging times where the beat-note record is uniformly sampled and represents the quasi-static frequency hold condition.

We further note that the Rb-disciplined ADEV does not exhibit ideal $1/\sqrt{\tau}$ scaling because the resonator is only loosely disciplined to the atomic reference. The finite spectroscopy discriminator slope, residual servo error, and environmental perturbations limit the transfer of atomic-reference stability to the resonator, resulting in Allan deviation behavior that differs from that expected for purely white-frequency noise.

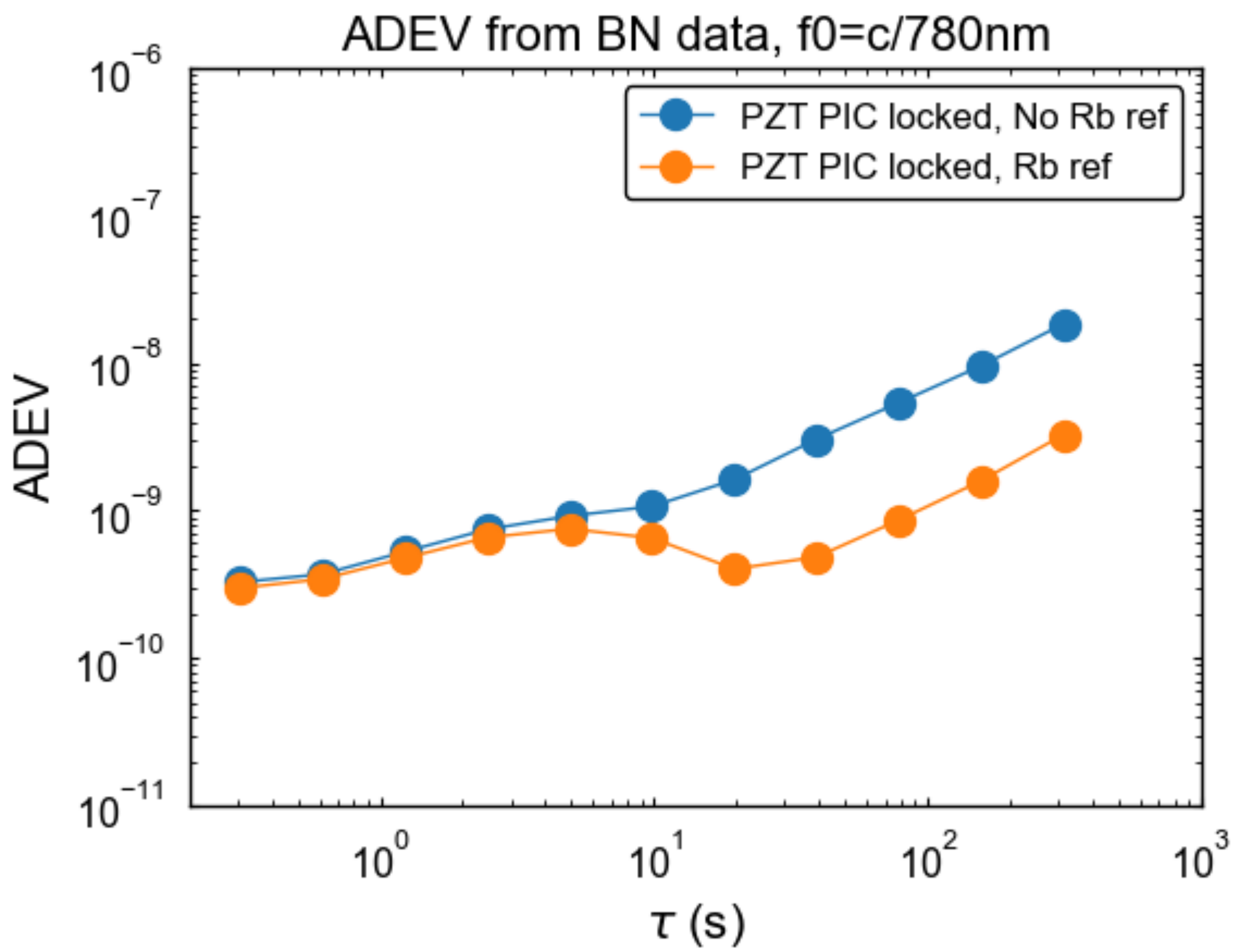

Frequency-noise spectrum calculated from the beat-note frequency time series. Below the faster motion of the resonator creates extra frequency content which makes it difficult to evaluate meaningful metrics. We feel that the lower offset frequencies are more relevant and we choose to just include the ADEV data for this particular measurement.

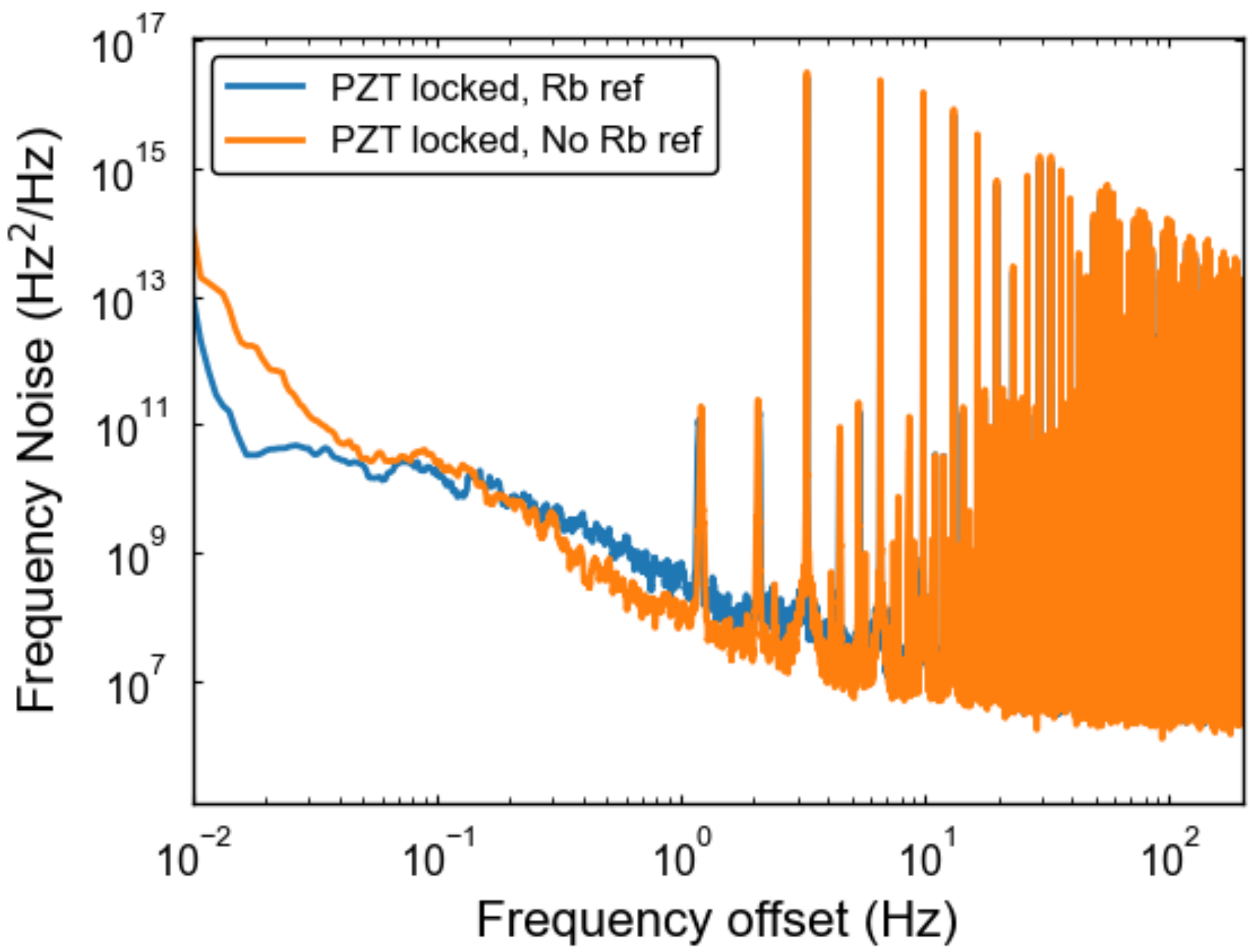

Revision: Supplementary Information, added new Figure S4

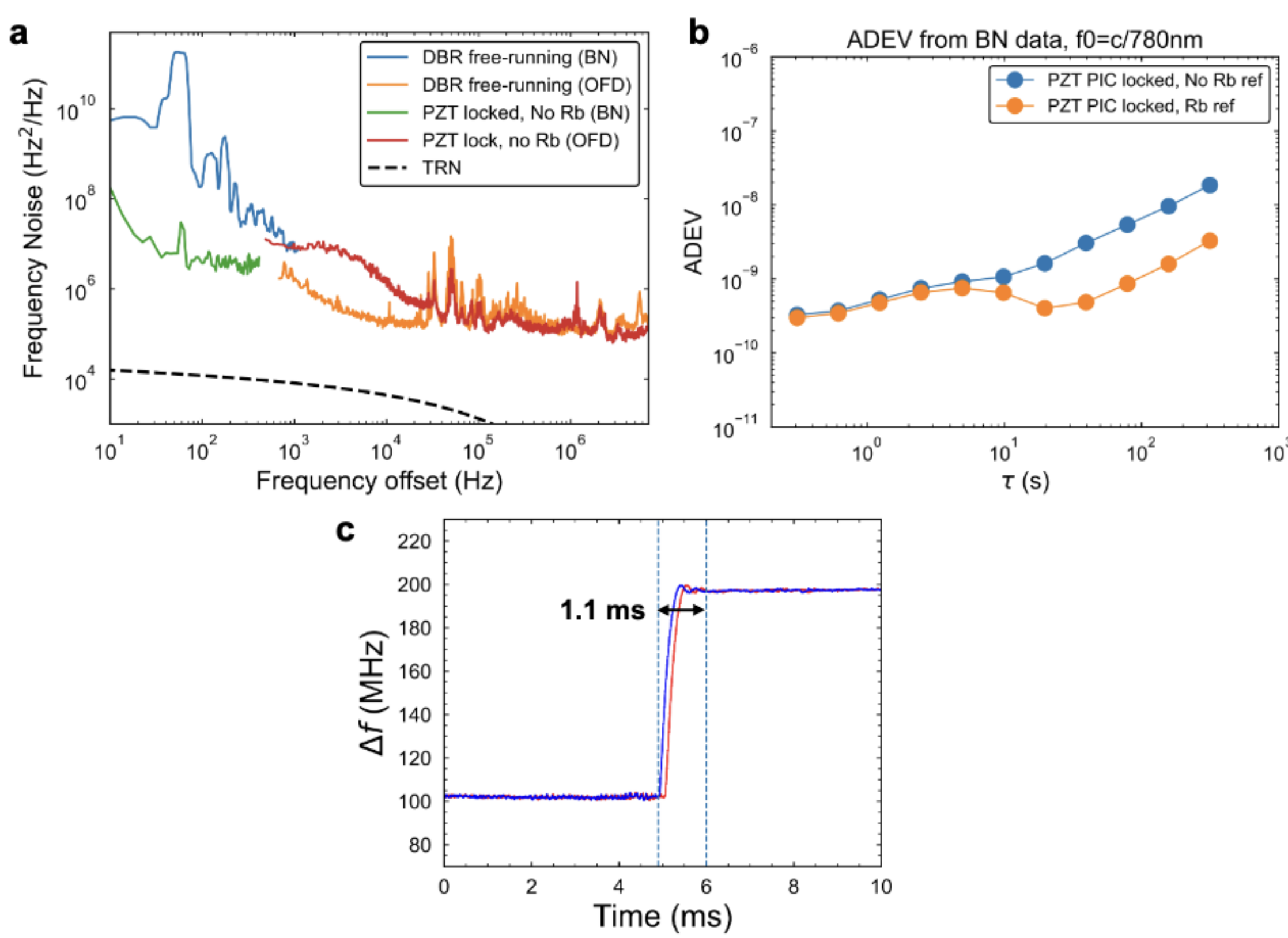

Supplementary Figure 4: Frequency-noise, stability, and agility measurements. a) Frequency-noise spectra of the free-running and PZT-locked 780 nm DBR laser measured using both an optical frequency discriminator (OFD) and a laser–laser beat-note (BN). The PZT lock suppresses low-frequency laser noise, while residual frequency noise is limited by the PDH discriminator slope and servo noise. The dashed black curve shows the calculated thermo-refractive noise (TRN) limit of the resonator. b) Allan deviation (ADEV) calculated from the beat-note frequency time series of Fig. 5B for operation with and without Rb disciplining. The Rb-disciplined configuration exhibits improved long-term frequency stability for averaging times above approximately 4 s. c) Laser beat-note of the PZT-locked laser during a voltage step applied to the PZT actuator measured with a 10 μs frequency counter gate time. A 100 MHz frequency step exhibits a rise time of approximately 300 μs and settles to the final value in approximately 1.1 ms.

Revision: Supplementary Note 1, Laser locking, last paragraph

"The laser frequency-noise spectra measured using both an optical frequency discriminator (OFD) and an independent laser--laser beat note are shown in Supplementary Fig 4a. Locking the DBR laser to the PZT-actuated resonator suppresses low-frequency frequency noise relative to the free-running laser, while the residual frequency noise is limited by the modest PDH discriminator slope and servo noise. Based on the cavity linewidth (184 MHz), detected optical power ~50 uW, and agreement between the measured in-loop and out-of-loop noise spectra, we estimate a PDH discriminator slope of approximately 1-5 nV Hz^{-1}. Supplementary Fig.4b further shows that Rb disciplining improves long-term frequency stability for averaging times above approximately 4 s. We further note that the Rb-disciplined ADEV does not exhibit ideal $\tau^{-1/2}$ scaling because the resonator is only loosely disciplined to the atomic reference. The finite spectroscopy discriminator slope, residual servo error, and environmental perturbations limit the transfer of atomic-reference stability to the resonator, resulting in Allan deviation behavior that differs from that expected for purely white-frequency noise. In addition to frequency stability, rapid and repeatable frequency actuation is required for laser-cooling sequences. Supplementary Fig.4c therefore characterizes the dynamic response of the PZT actuator. A 100 MHz programmed frequency step exhibits a 10-90% rise time of approximately 300 μs and settles within 1.1 ms, confirming the agility required for sub-Doppler cooling sequences."

In your discussions, please address:
o How does PZT hysteresis influence the frequency stability?

Response: The dominant source of long-term frequency drift is the resonance-frequency drift of the SiN cavity, arising from environmental perturbations such as temperature fluctuations and the thermo-optic effect. The dual-stage locking architecture periodically references the cavity-stabilized laser to the Rb transition and compensates for these slow drifts. However, because the atomic correction is applied periodically rather than continuously, the cavity resonance may drift between successive Rb updates. PZT hysteresis introduces an additional source of uncertainty by causing the relationship between applied voltage and resonance-frequency shift to depend on the recent actuation history. As a result, a fixed control voltage does not always correspond to the same frequency offset. In our system, the Rb reference is used to calibrate the cavity resonance frequency, after which predetermined voltage steps are applied to generate the desired frequency

jumps. Consequently, the absolute frequency reached after a given voltage step can vary slightly from cycle to cycle due to PZT hysteresis and cavity drift.

Revision: Main text, "Resonator agility measurements", paragraph 2:

"Figure 5b shows the laser beat-note recorded over 20 minutes of continuous operation. While we periodically referenced the PZT cavity to the ^{87}Rb SAS (top), the beat-note remained stable near 78 MHz during the ^{87}Rb lock. Slow drift in resonator response to a fixed PZT drive voltage leads to a gradual shift in cooling-light detuning during extended operation, particularly during the first hour after system startup. Quantitatively, Fig. 5b shows a 0.7 MHz/min (predominantly upward) drift in cooling detuning, Δ_{MOT}. This behavior is attributed to long-timescale effects in the PZT actuator. These effects, as well as hysteresis and creep[9], are discussed further in Supplementary Note 1."

Revision: Supplementary Note 1, paragraph 3 (these references found at the end of this document):

The PZT actuator exhibits hysteresis and creep which can affect the absolute frequency of the locked laser after the spectroscopy referencing step. PZT hysteresis is a reversible, history-dependent response between applied voltage and induced strain within a single voltage sweep, while creep describes a time-dependent strain relaxation following a single voltage step[9]. This differs from the single-MHz-scale, minute-scale drift observed in Fig. 5b which is attributed to long-timescale changes in effective mechanical response of the actuator, consistent with accumulated domain evolution and thermal or fatigue-related effects in the PZT material. While hysteresis and creep are endemic to piezoelectric actuation, their effects on frequency positioning can be compensated by closed-loop control[7,8,10].

o What are the noise and drift sources that affect short-term and long-term stability?

Response: The dominant sources of short-term and long-term instability are different. Short-term stability is primarily limited by the strength of the PDH lock to the photonic resonator. Due to the relatively low cavity Q factor and extinction ratio, the PDH discriminator slope is small, limiting the achievable suppression of the free-running laser frequency noise. In addition, the weak discriminator slope increases the sensitivity of the lock to servo and detector noise, which contributes to the residual frequency noise observed at short averaging times.

Long-term stability is limited by the accuracy with which the resonator can be referenced to the Rb transition. The atomic lock also exhibits a relatively small error-signal slope, limiting the correction bandwidth and the ability of the resonator resonance to continuously track the Rb hyperfine transition. Additional long-term instability arises from drift of the SiN resonator resonance frequency, PZT hysteresis, temperature fluctuations, and systematic effects within the saturated-absorption reference, including optical-power fluctuations, and environmental perturbations of the spectroscopy setup. These effects are substantially reduced, but not entirely eliminated, by the Rb disciplining loop. A more detailed discussion of the atomic-referenced locking architecture and its associated noise sources is provided in Ref. [2].

o How does stability vary as the interval between disciplining events changes (i.e. with different cycle times)?

Response: The single-cycle stability, in terms of random frequency wander, proved insignificant at the different intervals between frequency-disciplining events. We have tried 100 ms, 290 ms, and 500 ms, as listed in a different reviewer comment response. However, a different factor *is* very tied to this parameter. We refer the reviewer to Figure 5d, which not only demonstrates the high single-cycle stability of a 290 ms interval between disciplining, but also shows a settling time where the cooling laser frequency takes some time (~50 ms) to fully settle into the final MOT formation frequency. This settling time is consistent, yet can prove troublesome. We aim to address this challenge in future work by more advanced voltage waveforms sent to the PZT. We have added data for the control voltage jump locked laser frequency response, see the response in the comment below for *Switching Time*.

Revision: Main text, Discussion:

"…Additionally, we are developing an FPGA-based controller for complex waveform generation, data processing, and feedback control to increase frequency precision and agility [7, 8, 10]. This system concept represents a path toward a chip-scale cold-atom instrument where laser frequency, power, and atomic readout are all governed by the same photonic platform."

9. Please quantify the agility of the PZT-tuned resonator, especially for applications requiring rapid cold atom generation. For example:

o What is the switching time between two frequencies?
o Does the switching time depend on the tuning direction (i.e., is there hysteresis)?
o Does it scale with the frequency difference between the starting and target frequencies?
o Could you define a "slew rate" for the resonator?

Response:

Switching time. We have added measurements of the step response of the PZT-actuated resonator using a shorter frequency-counter gate time (10 μs) than that reported in the main text (1.2 ms). For a 100 MHz frequency step, the laser frequency settles to within approximately 2% of its final beat-note value in approximately 1.1 ms. The corresponding 10–90% rise time is approximately 300 μs. The switching dynamics are determined by the combined response of the PZT actuator, photonic resonator, and PDH feedback loop. The switching time is expected to increase with larger frequency excursions, since the actuator must traverse a larger displacement and the feedback loop must reacquire the desired operating point over a larger tuning range. We did not perform a systematic characterization of switching time as a function of tuning direction. However, the dynamic tuning measurements presented in the manuscript show that the resonator closely follows both increasing and decreasing tuning commands.

Slew rate. The effective tuning slew rate can be defined as the frequency excursion divided by the 10–90% rise time. For the 100 MHz step shown here, this corresponds to an effective slew rate of approximately 0.33 MHz/μs.

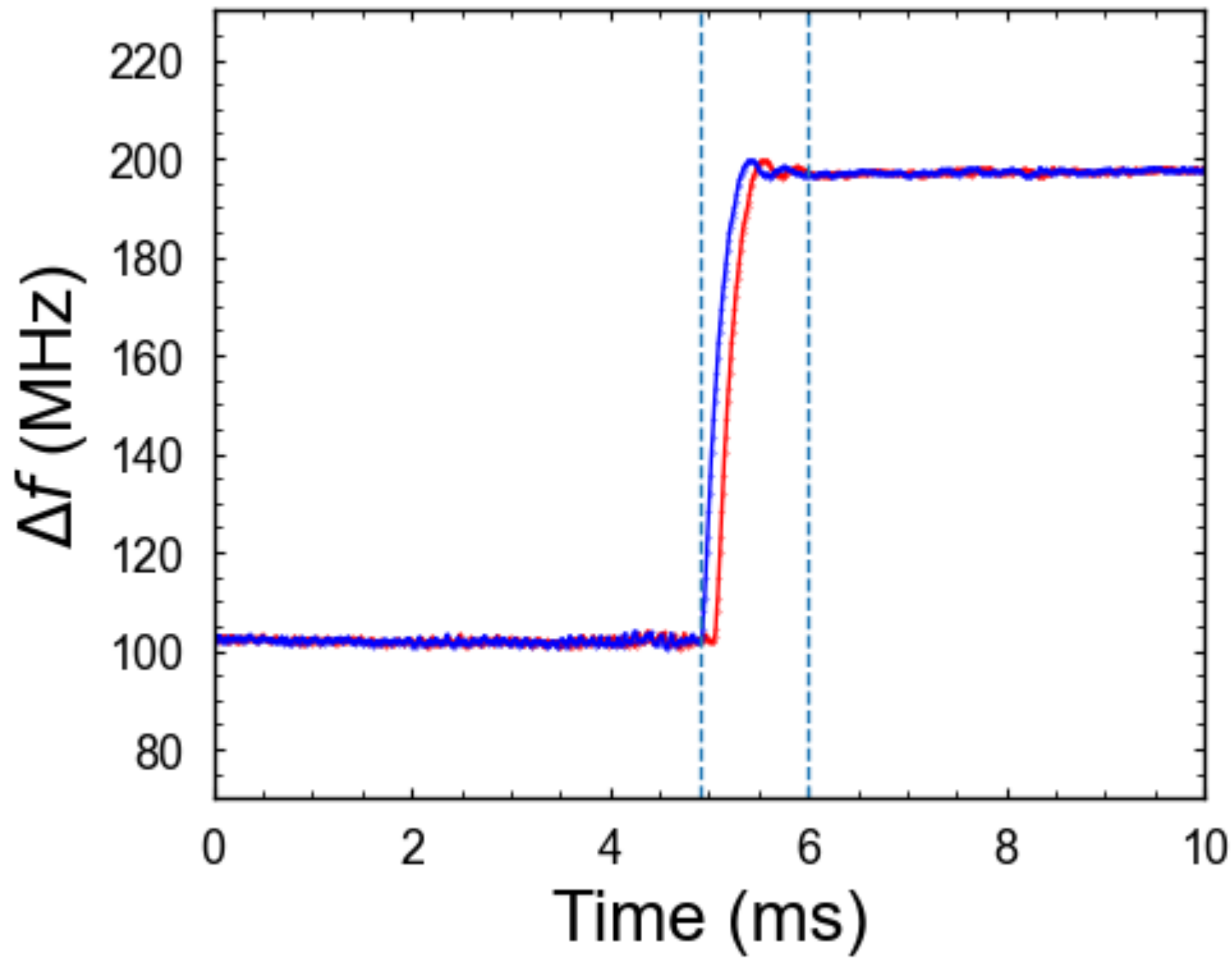

Revision: Main text, "Resonator agility measurements":

"Because the resonance shift is a stress-optic mechanical effect and the PDH laser-to-PIC lock must follow that shift (set by the PDH locking range and strength), the switching time depends on the frequency difference between starting and target frequencies. In a benchmark test, a 100 MHz frequency jump takes 300 μs, giving an effective slew rate of 0.33 MHz/μs (Supplementary Fig. 4c)."

Revision: Supplementary Information, added new Supplementary Figure 4:

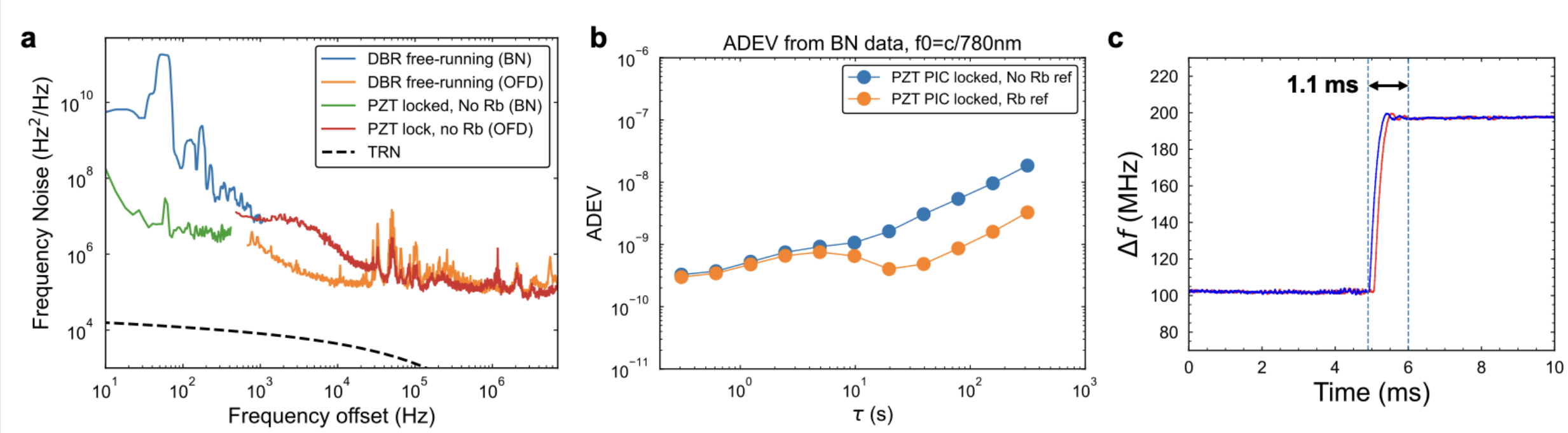

Supplementary Fig. 4: Frequency-noise, stability, and agility measurements. a) Frequency-noise spectra of the free-running and PZT-locked 780 nm DBR laser measured using both an optical frequency discriminator (OFD) and a laser–laser beat-note (BN). The PZT lock suppresses low-frequency laser noise, while residual frequency noise is limited by the PDH discriminator slope and servo noise. The dashed black curve shows the calculated thermo-refractive noise (TRN) limit of the resonator. b) Allan deviation (ADEV) calculated from the beat-note frequency time series

of Fig. 5B for operation with and without Rb disciplining. The Rb-disciplined configuration exhibits improved long-term frequency stability for averaging times above approximately 4 s. c) Laser beat-note of the PZT-locked laser during a voltage step applied to the PZT actuator measured with a 10 μs frequency counter gate time. A 100 MHz frequency step exhibits a rise time of approximately 300 μs and settles to the final value in approximately 1.1 ms.

Comments on the Figures:

• Figure 1: Working principle of rubidium sub-Doppler cooling using an agile integrated PZT-controlled silicon nitride cavity.
- Please consider including an energy level diagram showing the relevant laser transitions

Response: We thank the reviewer for the suggestion. We have added a simplified energy-level diagram to Fig. 1a illustrating the cooling and repump transitions in ^{87}Rb, along with the frequency-agile detuning applied during the sub-Doppler cooling sequence. The purpose of this panel is to provide a high-level overview of the role of the agile 780-nm cooling laser within the experimental sequence. A more detailed energy-level and laser-transition diagram has been added to the Supplementary Information (Supple. Fig. 5) and is referenced in the main text.

Revision: Main text, Figure 1

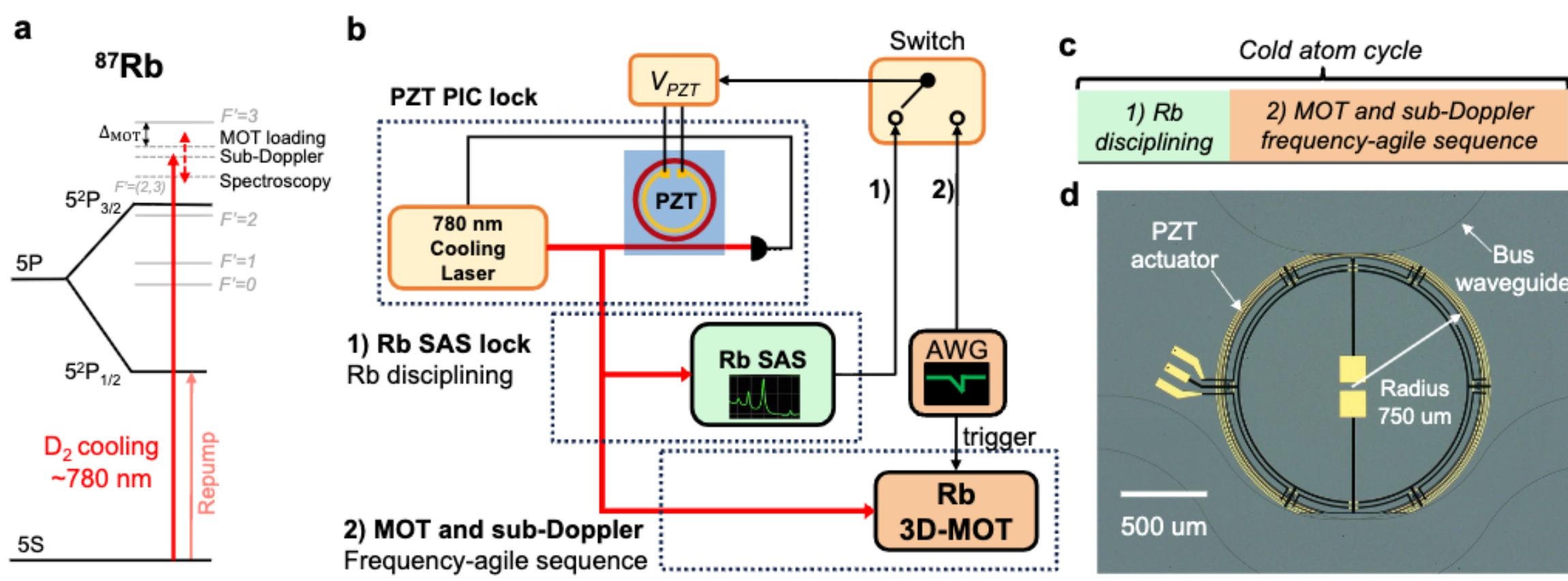

Figure 1: Working principle of rubidium-87 (^{87}Rb) sub-Doppler cooling using an agile integrated PZT-controlled silicon nitride cavity. a) Simplified ^{87}Rb energy-level diagram showing the cooling and repump transitions and the rapid PZT-controlled detuning of the 780 nm cooling laser used to transition from magneto-optical trap (MOT) loading to sub-Doppler polarization-gradient cooling (PGC). b) A 780-nm distributed Bragg reflector (DBR) laser is Pound-Drever-Hall (PDH) locked to a photonic integrated circuit (PIC) resonator containing a lead zirconate titanate (PZT) stress-optic actuator.…"

Revision: Supplementary Information, "MOT system and characterization and measurement"

"...The relevant energy level transitions are shown in Supplementary Fig. 5."

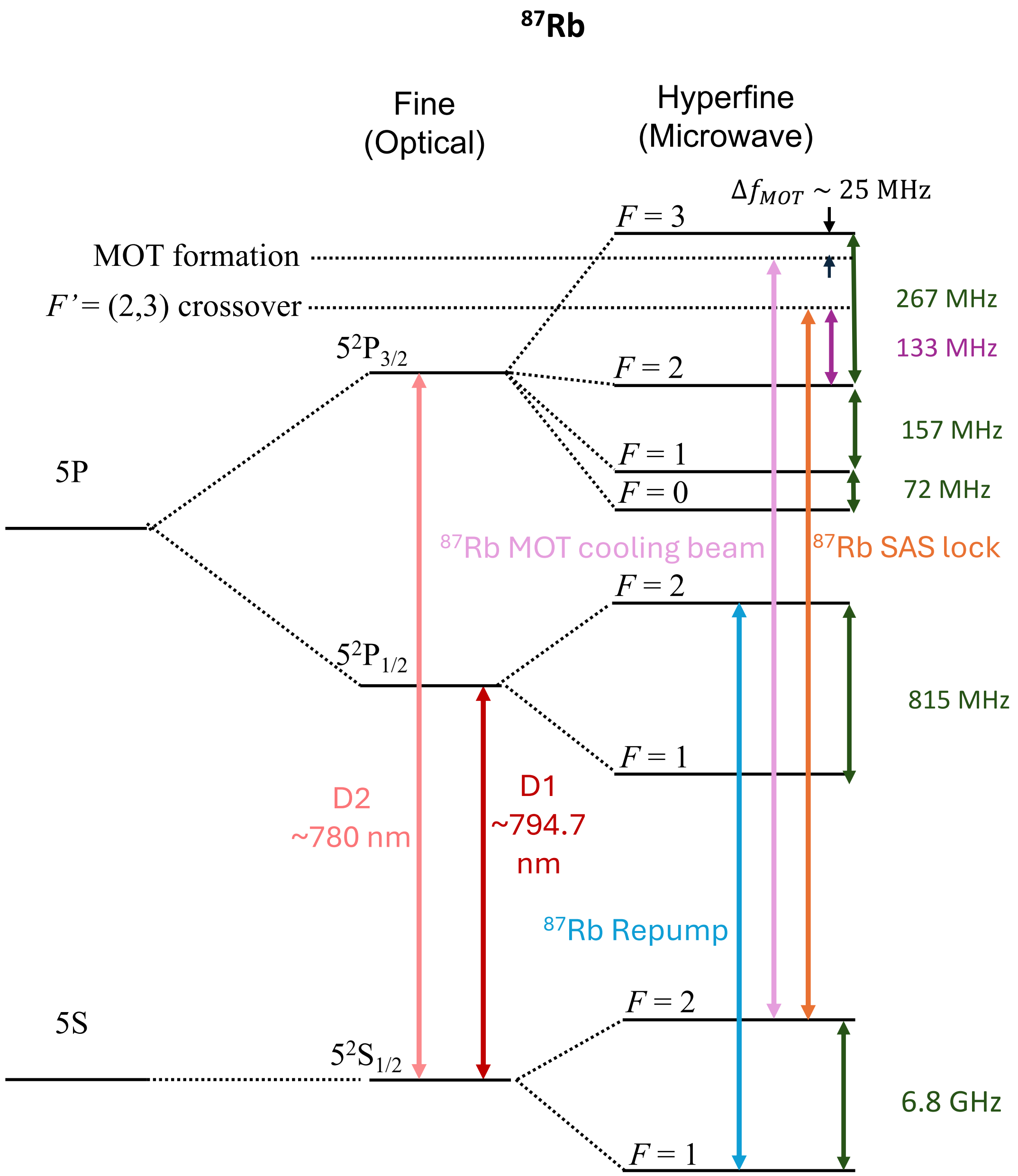

"**Supplementary Fig. 5: Energy level diagram.** Energy level diagram of ^{87}Rb including the D1 (light red) and D2 (dark red) transitions and the hyperfine structure. Relevant experimental laser frequencies are color coded: the 795-nm repump beam (light blue), and the 780-nm MOT cooling beam (pink, red-detuned by 25 MHz) and the ^{87}Rb saturation absorption spectroscopy (SAS) lock frequency (orange). During the MOT formation, the cooling laser rapidly switches between the green and orange frequency settings by means of the PZT-actuated resonator."

and clarify which parts of your laser system are COTS and which are custom, PIC-based subcomponents.

Response: We have updated the main text ("Results" section) to better specify which parts of our laser system are COTS and which are custom (see revision below).

Revision: Main text, Results, first paragraph

"...anchoring the PIC-locked laser to an atomic transition and reducing the long-term laser frequency drift. Our laser system combines commercial off-the-shelf (COTS) components with a custom PIC. The COTS components include the cooling and repump DBR lasers, a fiber-coupled SOA for optical power modulation and shuttering, and the free-space ^{87}Rb SAS setup. Combined with SOA-based power modulation, the PIC-based frequency control removes the need for external acousto-optic or electro-optic modulators (AOMs/EOMs) for sub-Doppler cooling."

• Figure 2: PZT PIC resonator design and characterization.
- What is the mechanism affecting the variation in the tuning strength (MHz/V) among the different dies?

Response: The variation in the tuning strength positive and negative is due to the offset between the PZT electrodes which affects the stress field and therefore the stress-optic tuning effect. For devices of the same offset, variation in the tuning strength depends on the modal overlap between the waveguide mode and the stress field, and the PZT film quality and uniformity across the wafer.

Revision: Main text, Figure 2 caption

"**Fig. 2: PZT PIC resonator design and characterization. … f)** Map of the 4" wafer DUV stepper lithography exposure reticles. Each reticle contains many devices; we report the data (PZT tuning strength, Q_i) for only a single add-thru resonator device in the reticle. Green outline: device with largest tuning strength, blue outline: device used for atom cooling demonstration, N/A: not applicable due to device damage. Variation in tuning strengths between dies is due to differences in PZT electrode offsets (which affects the stress field), variations in modal overlap between the waveguide and the stress field, and variations in PZT film quality/uniformity."

• Figure 3: Cold atom experiment diagram.
- You currently lock the cavity (and thus the laser) to the F=2-> F'=2,3 crossover in your SAS cell, then apply a PZT-voltage jump to reach the true F=2 -> F'=3 cooling line.
- Have you tried instead to lock to the F=2 -> F'=3 peak? That would avoid the per-cycle frequency jump and may lead to better long-term stability in terms of PZT cavity control.

Response: The reviewer is correct that locking to the F=2 → F'=3 peak directly would improve long-term stability of PZT cavity control. However, the saturated absorption feature is 6-times weaker for F=2 → F'=3 in comparison to the cross-over which potentially limits robustness in the re-referencing stage. Moreover, a F=2 → F'=3 reference point would still require a frequency jump (albeit a much smaller one) to achieve ideal detuning for MOT formation (and later another ramp for PGC).

Revision: Main text, "Sub-Doppler atom cooling":

"The timing diagram for one experimental cycle is shown in Figure 3C. Each sequence begins with ^{87}Rb disciplining at the F' = (2, 3) crossover transition for 25 ms. While F'=3 may be a

more natural locking point since it matches the cooling transition, the F' = (2, 3) crossover feature is used instead since it has a six-fold better signal-to-noise ratio (SNR) in the absorption. This allows robust and consistent laser relocking during ^{87}Rb re-referencing."

o What is the maximum off-resonance hold time (after disabling the F'=2,3 crossover lock and jumping to F'=3)? Can you extend this hold time by using a small frequency jump or scan range—which perturbs the lasing condition less (for example, by locking to directly to F'=3)?

Response: We have tested progressively longer delays between referencing the F'=2,3 crossover lock. We were able to stably run the system with re-referencing delays up to 30 seconds. We were unable to test further delays at this time due to a breakdown in our control system after delays longer than 30 seconds (which we never anticipated in any experimental use case). Please see the plot below of the MOT fluorescence as measured by an APD during a 30 second delay between spectroscopy referencing. Please note that our APD has a very small sensitive area and with the spatial motion of the MOT we introduce some intensity noise not attributed to the stability of the cavity. Still, this demonstrates the ability to hold a reasonable frequency stability over long periods between spectroscopy referencing events. We have added a line to the paper's text to include the takeaway result of this test.

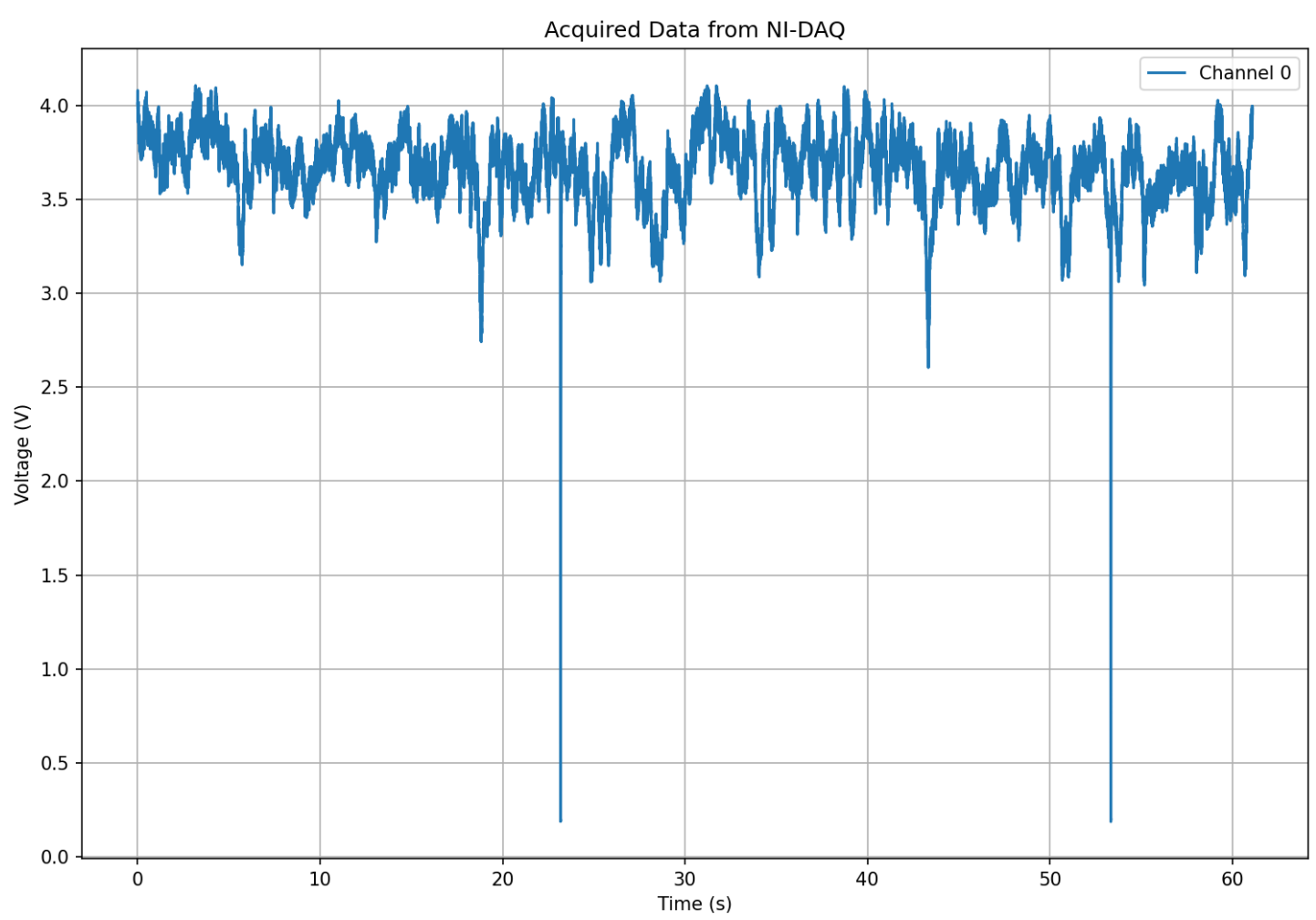

And zoomed in on one of those re-referencing dips:

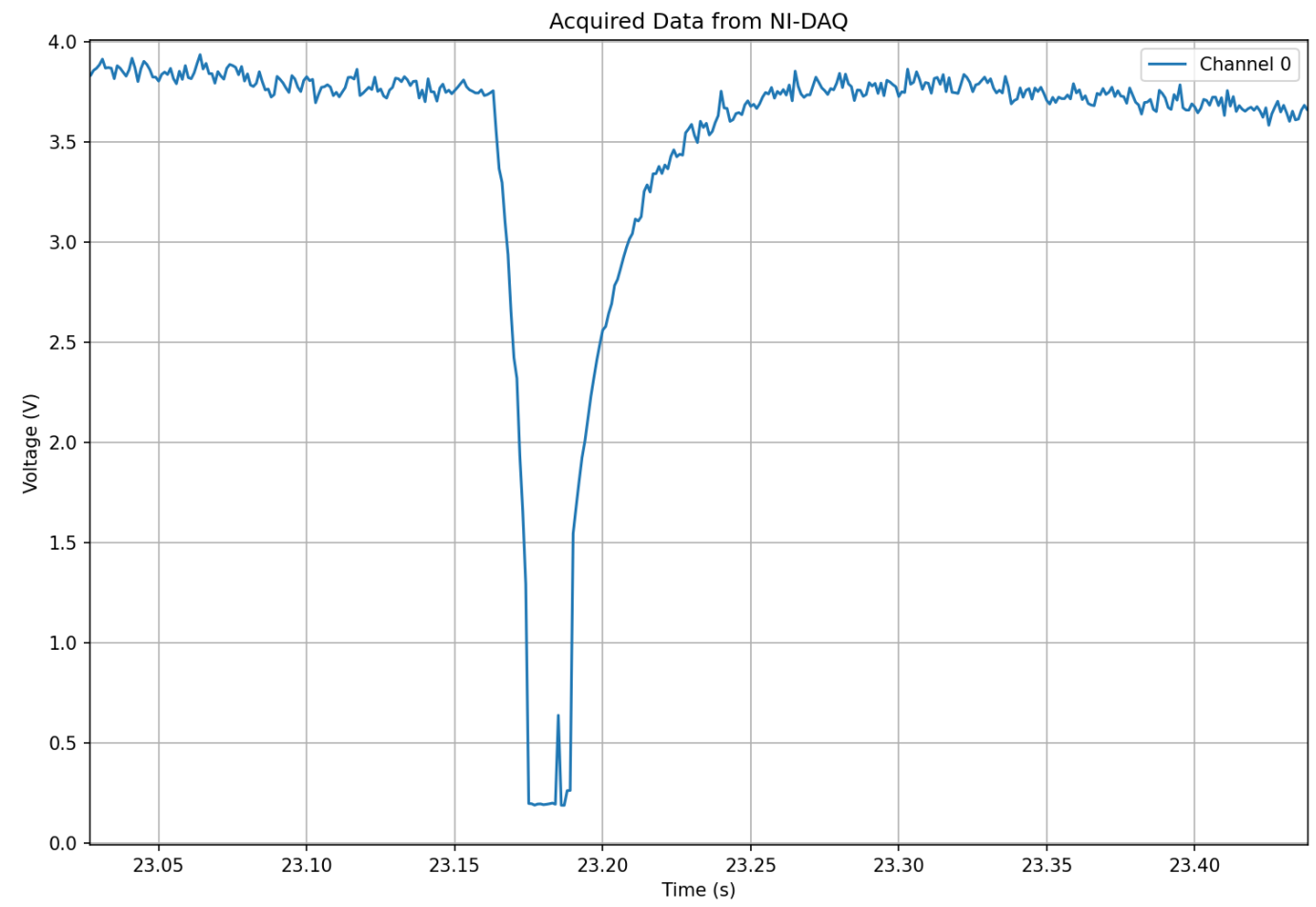

Revision: Main text, "Resonator agility measurements", third paragraph:

"In another stress-test of the methodology, we have attempted a 30 second MOT formation period (i.e. the spectroscopy is only referenced once every 30 seconds). We had no difficulty re-referencing spectroscopy after such a delay; this further demonstrates the long-term frequency drift suppression offered by the cavity."

• Figure 4: MOT temperature measurements for different cooling laser control configurations.
- Do you use the same parameters on cooling, repump, atom-detection/fluorescence beams and TOF measurement protocols for (I) No PGC, (II) PZT Freq ramp only, (III) PZT Freq ramp & SOA power ramp, and (IV) AOM reference?

Response: Yes, the same parameters are used. The caption for Supplementary Figure 6 has been modified to emphasize this, and the same addition is added here for clarity:

Revision: Main text, Figure 4 caption:

"In each case identical parameters were used for sequence timing, cooling and repump frequencies and powers, and fluorescence detection."

Revision: Supplementary Figure 6:
In each case identical parameters were used for sequence timing, cooling and repump frequencies and powers, and fluorescence detection

- Are the horizontal and vertical atomic temperatures the same?

Response: The horizontal and vertical atomic temperatures are indeed different, a common occurrence in MOTs due to different conditions in the transverse trapping beams and/or asymmetry in the field gradients. Figure 4 shows the cloud squared radii (as errorbars) along the z (dotted bars) and y (solid bars). The temperature is colder in the y-axis and slightly warmer in the z-axis.

The temperatures listed in the legend of Figure 4 are an average of the two axes. The caption has been modified to better reflect this. Please note the raw data for each axis here:

**Case I**	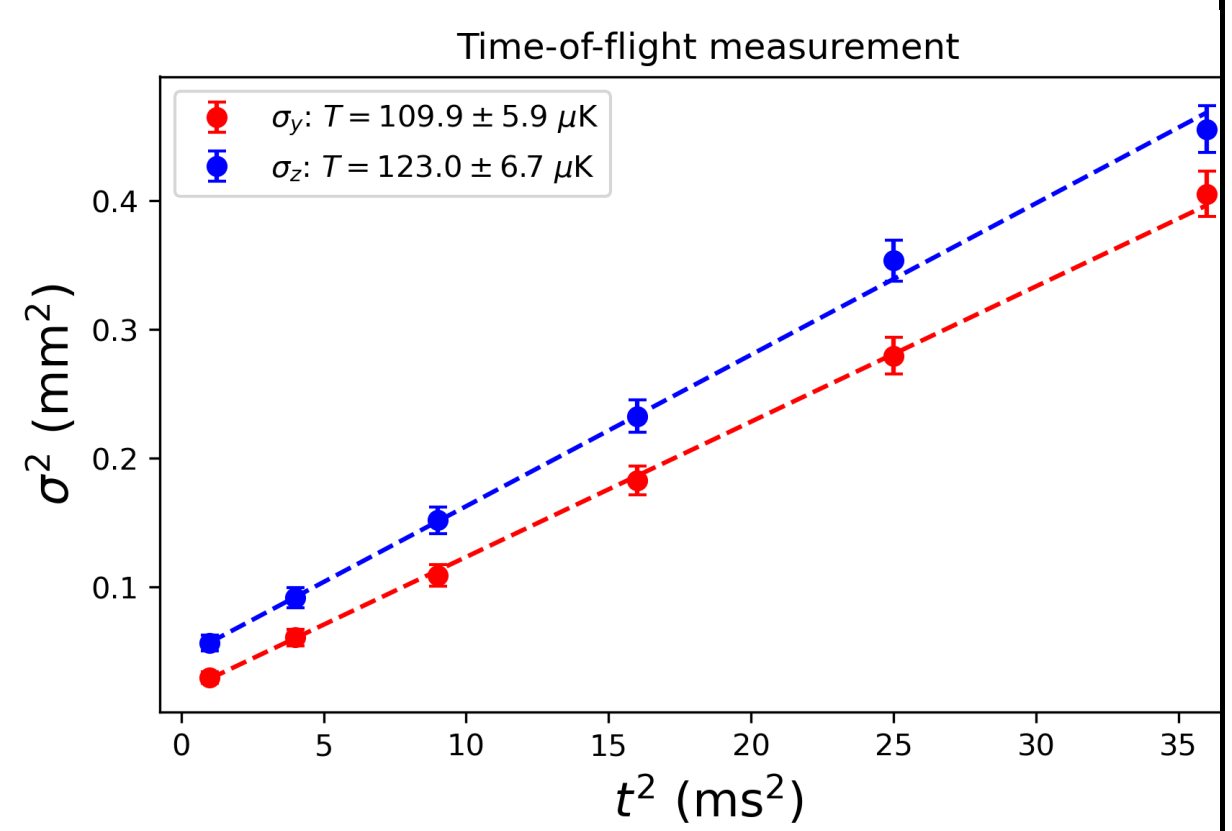
**Case II**	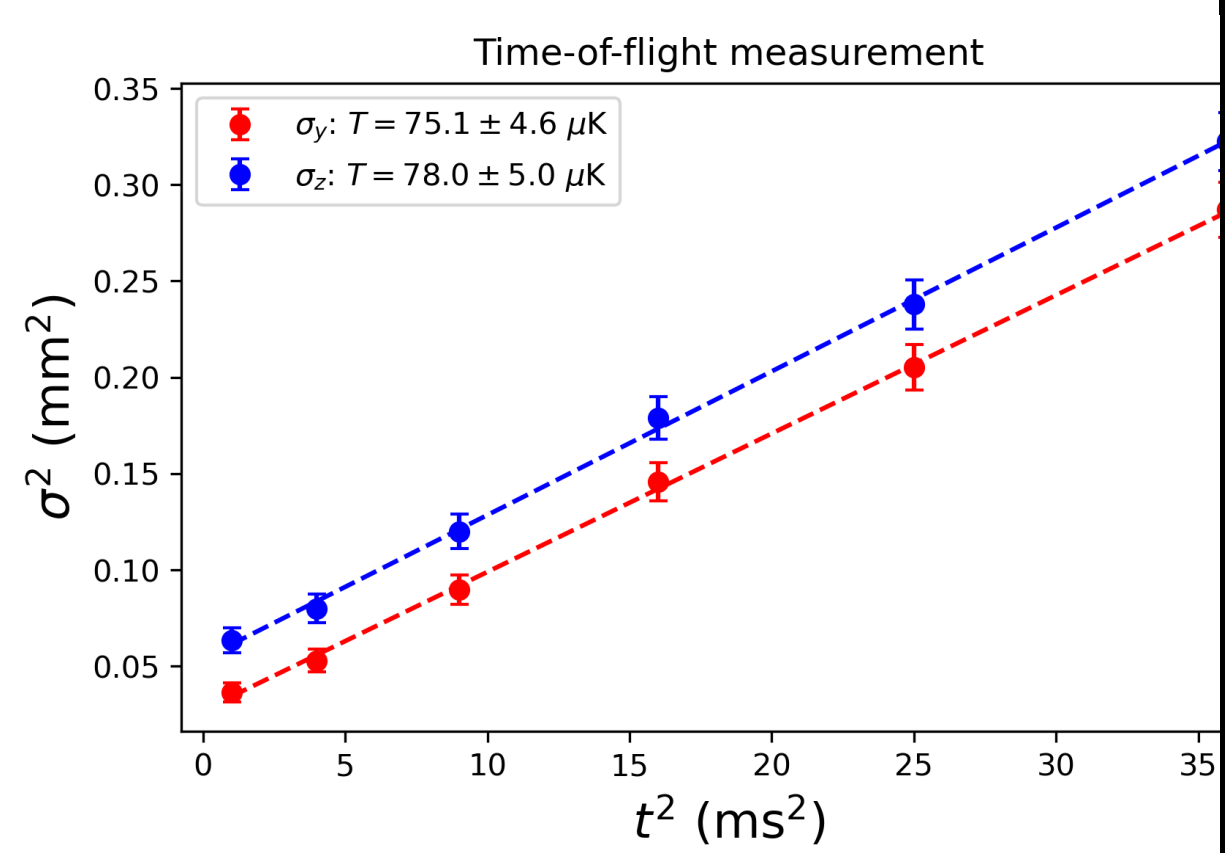
**Case III**	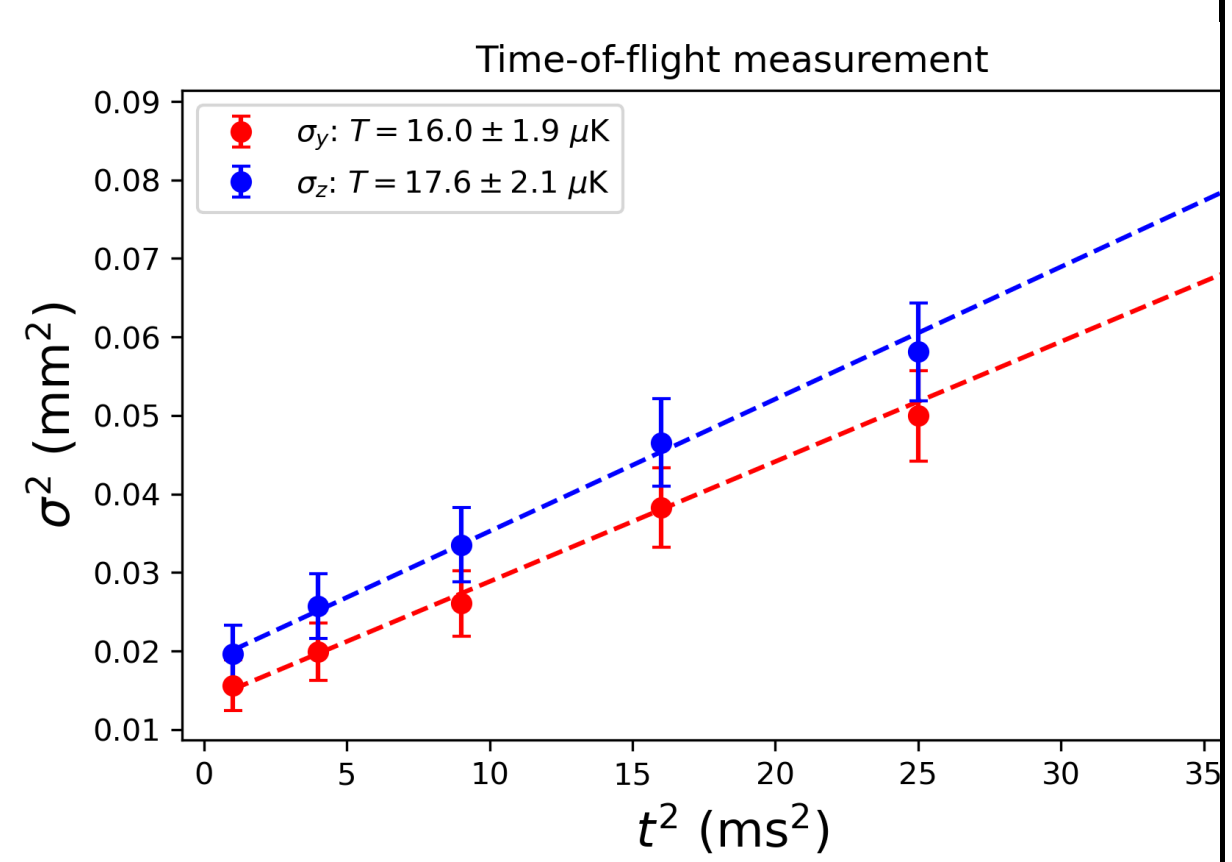
**Case IV**	

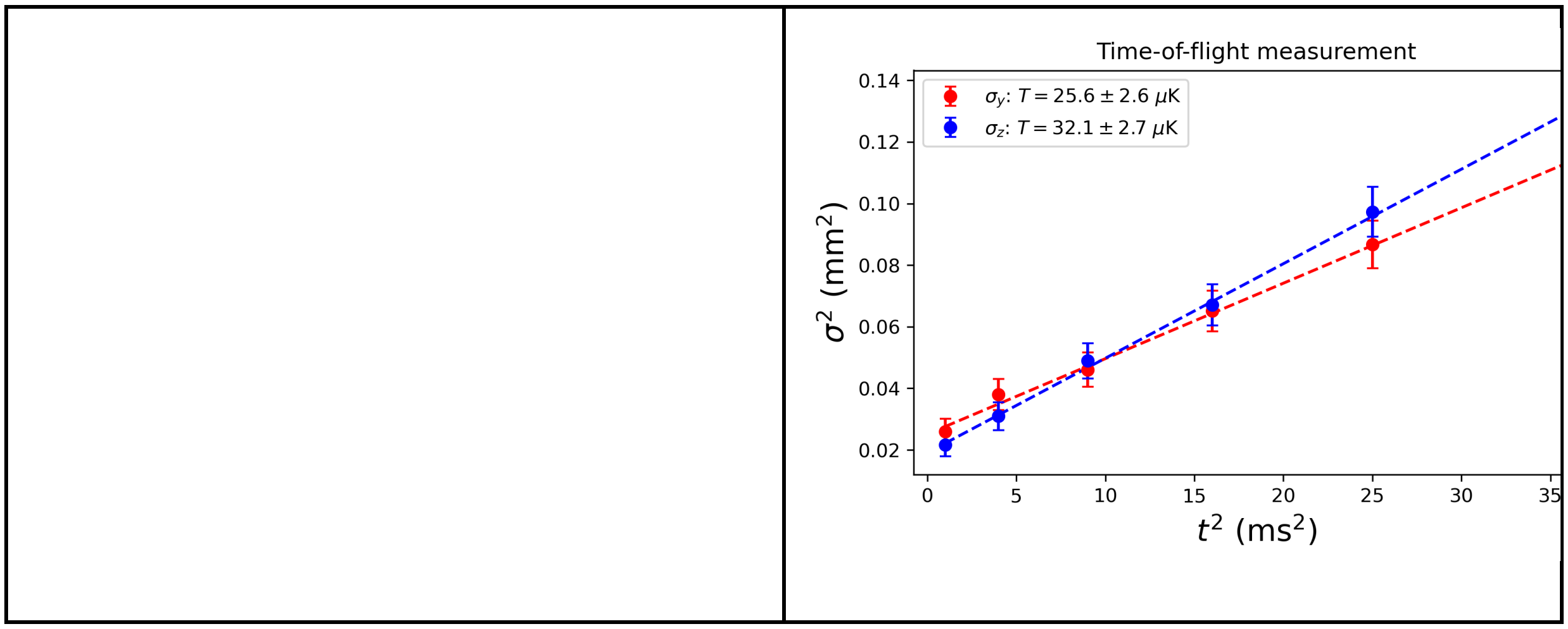

Revision: Main text, Figure 4 caption:

Figure 4: **MOT temperature measurements for different cooling laser control configurations.** **a)** Time-of-flight (TOF) atom cloud temperature measurements, with the squared cloud radius along the z (open markers) and y (filled markers) dimensions for different squared TOF times t^2_{TOF}. The linear fits are for $\sigma_i^2 = \sigma_{i,0}^2 + \frac{k_B T_i}{m} t^2_{\mathrm{TOF}}$, where σ_i is the Gaussian standard deviation of the cloud along an axis (y, z), $\sigma_{i,0}$ is the initial width of the cloud, k_B is the Boltzmann constant, m is the mass of a single ^{87}Rb atom, and T_i is the temperature along an axis. The temperatures reported in the legend are an average of both axial temperatures (which slightly differ due to asymmetries in beam alignment and in the magnetic field gradient in each axis). The schematic for the experiment

- Please include error bars on the data points.

Response: This has been resolved, see our response to your comment #4, above.

Revision: Updated text given in response to comment #4, above.

• Figure 5: Cooling laser frequency agility measurements.
- The changing atom number/fluorescence can be attributed to various sources. How can you know that the change in atom signal is primarily due to frequency changes? For example, are the laser intensities also stabilized? How does the frequency stabilities compare during the MOT vs probing stages?

Response: We agree that we should directly point the reader to our best data addressing this point: Supplementary Figure 8a. This figure shows the inverse relationship between the normalized mean squared error (NMSE) of a beat note measurement (between the cooling laser and a spectroscopy-locked reference laser) v. MOT brightness (monotonically related to atom number) over a 20-minute period, which displays strong correlation. We agree that the reader would benefit from this reference in the caption of Figure 5 so the additional line will be added (see below).

To address the reviewer's second question, frequency stability is effectively identical during the MOT formation and probing stages of our experiment. The probing takes place between 1 and 6 ms after the PGC (which itself is 12 ms in duration immediately after MOT formation). From the 100 cycle overlaid data in Figure 5d, we see strong consistency in laser frequency in both individual and short, minute-scale, comparisons of experimental cycles. It is only over a longer term (>1-minute timescales) that we observe an overall shift in laser frequency jumps. We have additionally added a sentence to Figure 5d caption (see below).

Revision: Main text, Figure 5 caption:

"Supplementary Fig. 8a demonstrates a strong correlation between cooling laser frequency drift and trapped atom number changes, suggesting that laser frequency drift is a primary factor in the observed variation in the atom fluorescence.".

Revision: Main text, Figure 5d caption:

"Laser drift, which would smear the overlaid traces in this figure, only becomes prominent at timescales greater than one minute."

- Figure 5 remains qualitative. On page 8 (second paragraph), the authors describe a slow drift in MOT fluorescence over long continuous operation, which they attribute to frequency-jump errors when the PZT resonator moves from the "disciplined" voltage to the MOT-detuning voltage. However, no numerical value for this draft appears in Figure 5 or the main text.

Response: We have added and modified a paragraph in the main text to quantify the drift seen in Figure 5B. Please refer to the updated text below:

Revision: Main text, "Resonator agility measurements", paragraph 2:
Figure 5b shows the laser beat-note recorded over 20 minutes of continuous operation. While we periodically referenced the PZT cavity to the ^{87}Rb SAS (top), the beat-note remained stable near 78 MHz during the ^{87}Rb lock. Slow drift in resonator response to a fixed PZT drive voltage leads to a gradual shift in cooling-light detuning during extended operation, particularly during the first hour after system startup. Quantitatively, Fig. 5b shows a 0.7 MHz/min (predominantly upward) drift in cooling detuning, Δ_{MOT}. This behavior is attributed to long-timescale effects in the PZT actuator. These effects, as well as hysteresis and creep[9], are discussed further in Supplementary Note 1.

- In Supplementary Materials (regarding Figure S6 [Now labelled S8]), the authors mentioned "Without Rb-referencing, the cooling laser exhibits a ~50 MHz drift over 20 minutes, whereas with Rb-referencing, the drifts are reduced to the single-MHZ range over the same interval." With the SAS servo disabled, but that measurement does not directly characterize the short-term drift or hysteresis over a single 235 ms MOT + PGC cycle. The manuscript specifies a 223 ms MOT loading time followed by a 12 ms PGC ramp, implying that without re-referencing every 235 ms the cavity frequency would wander beyond the trap detuning.

Response: Thank you to the reviewer for bringing attention to an omission in our manuscript. For the data presented in the manuscript, it is true that we employ re-referencing every 290 ms (235 ms for MOT loading). What we neglected to state was that the 290 ms was selected as a tradeoff between optimizing SNR of the fluorescence measurement while keeping a sufficiently fast repetition rate for this proof-of-principle demonstration. We have since reduced this sequence time to 100 ms to achieve 10 Hz rates (data on the incremental improvement is not included in this paper).

In summary, the 290 ms sequence time has little to do with the limitations of short-term drift. In fact, significantly longer periods (such as second-scale) periods between re-referencing spectroscopy may still adequately suppress short-term frequency shifts. We have demonstrated re-referencing intervals of 30 seconds while sustaining a MOT (see earlier answer). We do not present the data since we cannot collect beatnote data at present since the frequency counter was a rental. Early in our experimental work, 500 ms sequences were used to similar effect. We add the following to a paragraph in the "Sub-Doppler atom cooling" section correct this omission.

Revision: In Main Text, "Sub-Doppler cooling" subsection:
The timing diagram for one experimental cycle is shown in Figure 3C. Each sequence begins with ^{87}Rb disciplining at the F' = (2,3) crossover transition for 25 ms. While F'=3 may be a more natural locking point since it matches the cooling transition, the F' = (2, 3) crossover feature is used instead since it has a six-fold better signal-to-noise ratio (SNR) in the absorption. This allows robust and consistent laser relocking during ^{87}Rb re-referencing. The PZT voltage is then stepped by $\sim$1 V to set the cooling laser at the MOT detuning Δ_{MOT} relative to the F'=3 cooling transition, and the MOT is loaded for 223 ms. Following MOT loading, the V_{agile} waveform linearly ramps the laser detuning by an additional 15 MHz over 12 ms to implement polarization-gradient cooling (PGC). After this ramp, all MOT and repump light is switched off at $t=250$ ms, initiating a TOF measurement with delays ranging from 0 to 6 ms. A 0.5 ms illumination pulse, applied at Δ_{MOT} (and optionally at $\Delta = 0$ in future runs), captures the atomic fluorescence image. The illumination pulse is generated by the cooling laser, which is shuttered from the atoms via an SOA during the time of flight (TOF). During this period, the laser is set to the MOT formation (red-detuned) frequency and subsequently flashed onto the atom cloud via SOA control. At *t=290* ms, the system re-engages ^{87}Rb referencing by first returning the PZT to the lock point and then re-enabling the SAS feedback. The 290-ms total interval between ^{87}Rb-spectroscopy re-referencing events was chosen to optimize fluorescence measurement SNR while keeping sufficiently fast (Hz-level) repetition rates needed for applications such as atom interferometry. Additional total interval times, including 100 ms and 500 ms, were tested without noticeable effects on single-cycle laser frequency drifts.

- Please clarify whether the PZT resonator's drift during one cooling cycle imposes such a limit and distinguish this slow drift from the separate issue of PZT hysteresis and frequency-jump accuracy.

Response: As answered in the above response, this drift during one cooling cycle does not impose such a limit. We agree that we should clearly distinguish this slow drift from the separate PZT hysteresis and frequency jump accuracy/speed (which is referred to as piezo creep). These

phenomena are found in all piezo devices and current work aims to better address these issues. The slow drift comes from a change in PZT mechanical response after repeatedly cycling the device and accumulated on minute scales. The hysteresis is a single cycle process which gives different mechanical responses depending on the direction of applied voltage change. The piezo creep is a single cycle process which stretches out the time for the piezo to reach its final position. Please refer to Pribošek et al. [9] for a more complete description of these effects and methods used to mitigate them, which are the subject of our ongoing work.

Revision: Supplementary Note 1, paragraph 3 (references written here refer to the numbering at the bottom of this document, not the revised manuscript):

The PZT actuator exhibits hysteresis and creep which can affect the absolute frequency of the locked laser after the spectroscopy referencing step. PZT hysteresis is a reversible, history-dependent response between applied voltage and induced strain within a single voltage sweep, while creep describes a time-dependent strain relaxation following a single voltage step[9]. This differs from the single-MHz-scale, minute-scale drift observed in Fig. 5b which is attributed to long-timescale changes in effective mechanical response of the actuator, consistent with accumulated domain evolution and thermal or fatigue-related effects in the PZT material. While hysteresis and creep are endemic to piezoelectric actuation, their effects on frequency positioning can be compensated by closed-loop control[7,8,10].

- Is the lower plot in figure 5B continuously holding the MOT, or is this also cycling the MOT load/detection cycle while just not re-referencing to the SAS signal in between?

Response: In the lower plot in Figure 5b, we still cycle the MOT between load/detection and provide the same "re-referencing" time as in the upper plot; however, at $t = 0$ we no longer allow the Vescent servo box to relock to the error signal of the atoms (as it does in the upper plot).

• Figure 6: Example integrated cold atom system enabled by the 780 PZT resonators.
- Fig. 6 shows the detailed vision for a fully integrated cold-atom instrument.
- Do you use a standard 3D MOT for this experiment, or a MOT with grating couplers as shown in Fig. 6?

Response: This 3D-MOT architecture is identical to that of the A. Isichenko et al *Nat. Commun* **14**, 3080 (2023) ref [10], i.e. the "PICMOT" architecture. We clarify that the retro-reflector mirrors are omitted for clarity.

Revision: Main text, Fig. 6 caption:

"The retro-reflector mirrors and waveplates are omitted from the 3D-MOT beam delivery portion for clarity."

• Figure S1: PZT device characterization.
- How did you address PZT hysteresis in the experiment?

Response: We acknowledge that PZT hysteresis is an unavoidable consequence of this method and this work is primarily focused on proof-of-principle demonstration rather than addressing hysteresis. Present, unfinished work concerns minimizing the impact of hysteresis by driving more complex waveforms to compensate for creep and hysteresis (see ref [11–14] in this document, which are now cited in the manuscript). In this work, hysteresis is a minimal enough issue that we can drive the laser away from the spectroscopy and return with the same voltage setting and still easily relock to the error signal. As seen in Supplementary Figure 1b, over extremely wide driving voltages hysteresis becomes a significant issue. Centered near the 3 to 10V driving voltages, behavior is much more linear.

To address the reviewer's comment, we add the following updated text to the outlook section of the discussion:

Revision: Main text, Discussion:

"We aim to implement power ramping and shuttering through critically coupled resonators driven by PZT amplitude control, while fluorescence feedback from the MOT provides frequency referencing. Additionally, we are developing an FPGA-based controller for complex waveform generation, data processing, and feedback control to increase frequency precision and agility [7, 8, 10]. This system concept represents a path toward a chip-scale cold-atom instrument where laser frequency, power, and atomic readout are all governed by the same photonic platform."

- Figure S1A shows an S21 measurement for die 6, but then figure S1B shows a hysteresis curve for die 3. Why are different characterizations being presented for different devices? Is data being cherry picked here, or was there some other reason for showing different data for different devices?

Response: Thank you for pointing out this inconsistency. We have updated the figure and caption below. The only difference is that our measurement method used for this die 3 was for modulation frequencies starting at 300 kHz compared to that of Fig. 2c (10 kHz). This figure has been updated to include data for both die 3 and die 6. Note that we now report hysteresis tuning data for both die 3 and die 6 in the updated Fig S1.

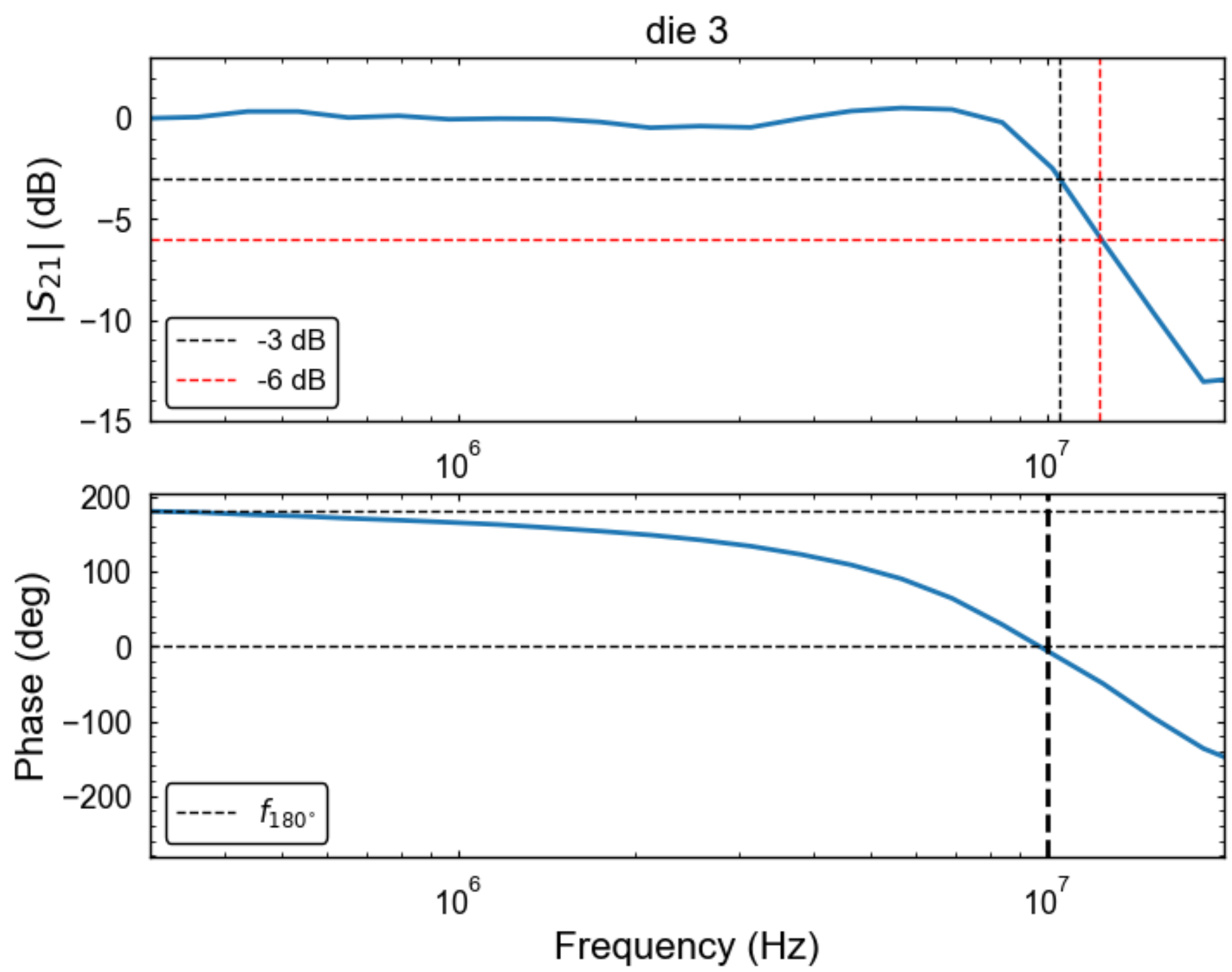

Revision: Supplementary information, Fig S1a

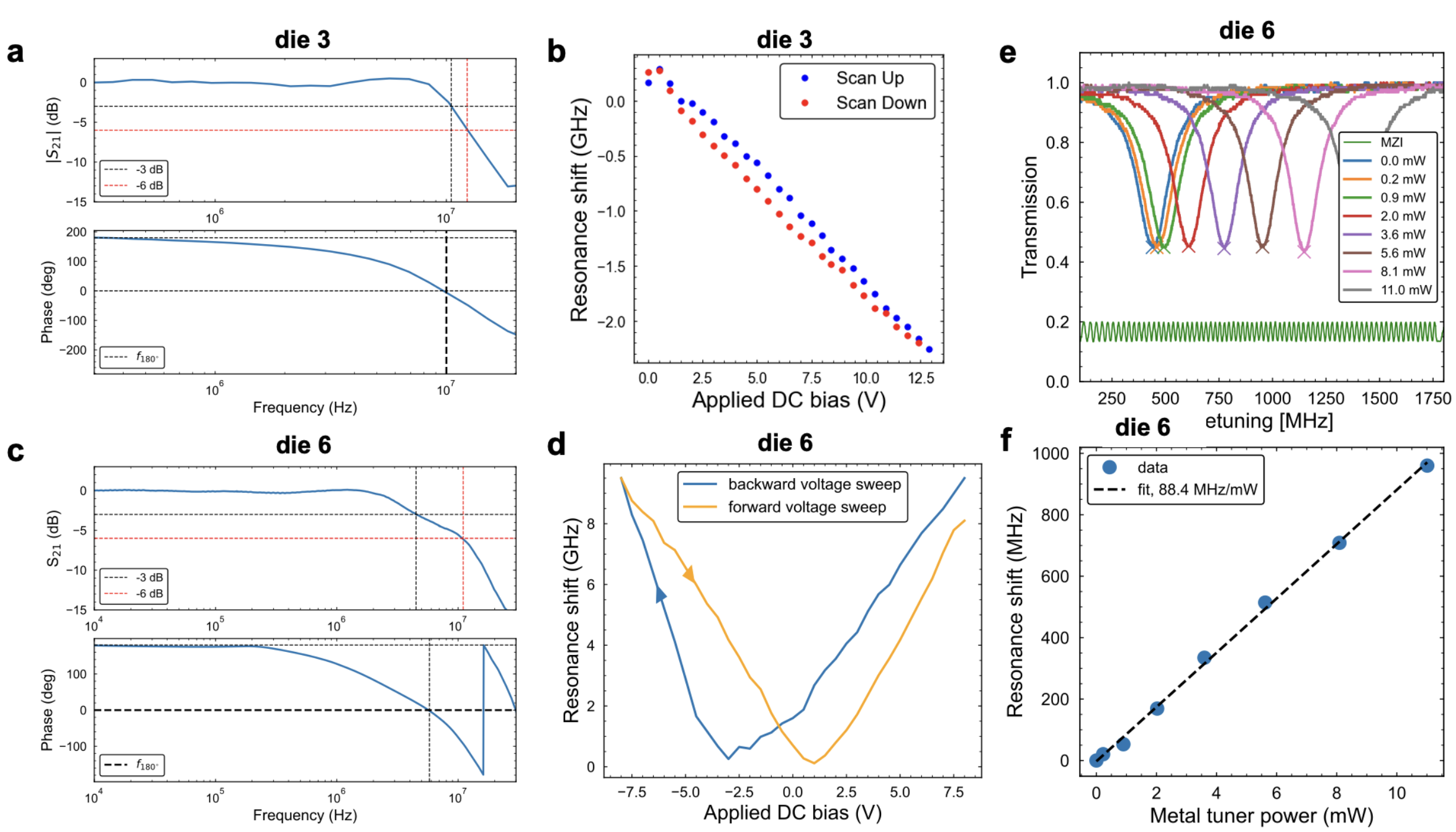

"**Supplementary Figure 1: PZT device characterization.** a) Frequency response measurements of device die 3 used for the sub-Doppler demonstration. The -3 dB and -6 dB response roll-offs are at 10.5 MHz and 12.3 MHz, respectively. The 180 degree phase lag point is at 10 MHz. b) Hysteresis measurements for die 3 for positive applied voltage. c) Frequency response

measurements of device die 6. The -3 dB and -6 dB response roll-offs are at 4.5 MHz and 11 MHz, respectively. d) Hysteresis for die 6, for voltage sweeps from negative to positive. e) Thermal tuner measurements for die 6, f) demonstrating a tuning strength of 88.4 MHz/mW."

• Figure S2: PZT PIC packaging.
- Could you explain the detailed optical power levels at each stage used for the PDH locking and the Rb SAS locking—accounting for all insertion losses, fiber-coupling losses, and fiber-to-waveguide coupling losses? Please start from the nominal output power of the 780 nm COTS laser (e.g., 50-100 mW).

Response: To provide more information on optical train power losses, a new section has been added to the supplementary information which can provide answers to the first part of this reviewer's question. This information is now pointed to in the caption of main text Figure 3.

Revision: Main text, Figure 3 caption

"A full description of optical train power losses is provided in the Supplementary Material."

Revision: Supplementary Information, New subsection "Optical train power losses":

"After isolation, the 780-nm cooling DBR laser outputs ~68 mW into free space at the chosen drive current. Of this, 2.4 mW is sent into the SAS branch. The elliptical beam profile of the DBR free-space output limits fiber-coupling efficiencies downstream. The remaining power is sent to the following arms:

- **Cooling arm.** We supply ~22 mW of free-space optical power to the fiber coupling into our polarization maintaining (PM) semiconductor optical amplifier (SOA). After amplification, the cooling light is launched back into free space by a collimator, giving a (Gaussian) free-space output power of 34 mW, most of which is bound for the atom trap. Separately, we deliver 3.5 mW of 795-nm repump light to the atom trap.
- **Resonator arm.** We supply ~16 mW of free-space optical power to the resonator arm. The light is coupled into a fiber and delivered toward the resonator through a fiber-to-fiber coupling, at which point we measure 1.4 mW; the coupler itself adds a further ~20\% loss. The packaged device then has a fiber-to-chip coupling loss of ~9 dB, higher than our typical 3-4 dB due to poor modal matching between the fiber mode and the PIC. The 900-nm waveguide width was not optimized for fiber coupling at the time of the device design. Adding a 250-nm taper is estimated to reduce the fiber-to-chip loss to aroud -3 dB and future devices for this SiN thickness will use this taper.
- The rest of the power is delivered to a beam dump."

Why the propagation loss (20 dB/m) in this paper is higher than another reference [30] (0.44 dB/m)?

Response: The devices used in this work have a more tightly confined mode and therefore higher propagation losses compared to previous work (0.44 dB/m) [2]. The waveguide dimensions are 900 nm by 120 nm, whereas reference 30 (as given in original manuscript reference ordering) is 4 μm

by 40 nm. Additionally, the devices from ref. 30 were fabricated at a CMOS foundry and the devices from this work were fabricated in the UCSB cleanroom facility. The devices in ref. 30 by M. Corato-Zanarella, et al. [15] achieved a propagation loss of 18 dB/cm at the wavelength 785 nm, due to a much more tightly confining waveguide which enables devices as small as 10-μm radius.

• Figure S3: Laser stabilization.
- You state that a 1.2 MHz direct-laser-current modulation is used to generate the error signals for both the PDH lock and the Rb SAS locks. In Rb-disciplining mode, you may need to scan and relock to the F=2 → F' = 2,3 crossover line. How much additional time do you need for this procedure? Do you leave on the laser output on Rb atoms during this scanning and relocking modes? (also, related to Fig. S5 [Now labelled Suppl. Fig. 7])

Response: We agree that Supplementary Figure 3 is misleading in this regard. Some of these comments are addressed in our response to this reviewer's comment #5, and we again include here the additional text added to address that point. As included in this paragraph, a frequency scan is unnecessary in this experiment. The issues of hysteresis are small enough (and the atomic crossover feature wide enough) such that driving the cavity back to the saved SAS lockpoint is always within the region of the error signal for the correct spectral crossover feature (F'=(2,3) crossover). In this data collection, we gave a longer-than-necessary settling time for the PZT before relocking to spectroscopy. Since this data collection, we have reduced the entire re-referencing process to just 7 ms (including 1.5 ms on either side for frequency switching and 4 ms for actual re-locking), including driving the PZT down to SAS frequency and back up to MOT formation frequency. At 7 ms, we experience no re-locking issues. Pushing much further can introduce instability due to the settling time of the PZT. We also add an additional line to the Supplementary Figure 3 caption to help make clear that such a frequency ramp as pictured in Supplementary Figure 3c is unnecessary during our experimental run.

Revision: Supplementary information, Supplementary Figure 3 caption:

"c) Error signal for the ^{87}Rb SAS while ramping the PZT with the laser-to-PIC lock engaged. From the known separation of the hyperfine peaks (F' = (1, 3) and F' = (2, 3), 75 MHz) we extract a frequency tuning strength of -190 MHz/V which is similar to the measured value in Figure 2. Note that a frequency sweep as shown in this figure is only necessary once when setting up the experiment. When re-referencing during looping experimental cycles, the PZT is simply driven back to a stored voltage setting corresponding to the F' = (2, 3) spectroscopy feature."

Revision: Main text, "Results":

"Each sequence begins with a 25-ms ^{87}Rb referencing interval followed by the MOT stage and concludes with a 12-ms triangular frequency ramp corresponding to the polarization-gradient cooling (PGC) sequence. The 25-ms ^{87}Rb referencing interval used for this data collection was an overly generous choice; it was selected to ensure re-locking stability. However, this re-referencing time has since been reduced to a 4-ms interval without detriment (or any additional control complexity)"

• Figure S4 [Now labelled Suppl. Fig. S6]: MOT system configurations for temperature measurements.

- Do you use the same parameters of cooling, repump, atom-detection/fluorescence beams and TOF protocols for (I) No PGC, (II) PZT Freq ramp only, (III) PZT Freq ramp & SOA power ramp, and (IV) AOM Freq ramp & SOA power ramp?

Response: We employ the exact same experimental sequence, cooling and repump frequencies and powers, and detection beam for cases I, II, III, and IV. The only differences are those described in the text and caption of Supplementary Figure 4 (now renamed 6). We add this line for clarity:

Revision: Supplementary information, Supplementary Figure 4 (now relabeled 6) caption:

"In each case identical parameters were used for sequence timing, cooling and repump frequencies and powers, and fluorescence detection."

• Figure S5 [Now labelled Suppl. Fig. S7]: Experiment timing.
- In Table S1, the symbol "Δ" is given as 25.5 MHz. Does this correspond to the MOT detuning (ΔMOT) labeled in Figure S5 [Now labelled Suppl. Fig. S7]? Please use the same notation in both the table and the figure.

Response: Yes, these have the same meaning. The symbol in the table will be updated for consistency.

Revision: Supplementary Material
All references to detuning are now written "Δ_{MOT}"

- In Figure S5 [Now labelled Suppl. Fig. S7], the SAS-reference detuning ΔSAS ref is 133 MHz (the frequency difference between the F'=2,3 crossover to the F'=3 line). Subtracting Δ_{MOT} (25.5 MHz) gives 107.5 MHz, yet the timing diagram labels this step as 118 MHz. Can you explain that discrepancy?

Response: This discrepancy was indeed an error. During all Cases (I through IV) the detuning 25.5 MHz was used for Δ_{MOT}. The 18 MHz corresponded to a different (non-MOT forming) beat note test we had performed. This value should not have been shown in the experimental control diagram. Thank you for pointing out this error. We have updated this figure with the figure shown below, which accurately tells the experimental story with 25.5 MHz detuning and a 107.5 MHz jump from the spectroscopy lock frequency.

Revision: Supplementary information, Supplementary Figure 7:

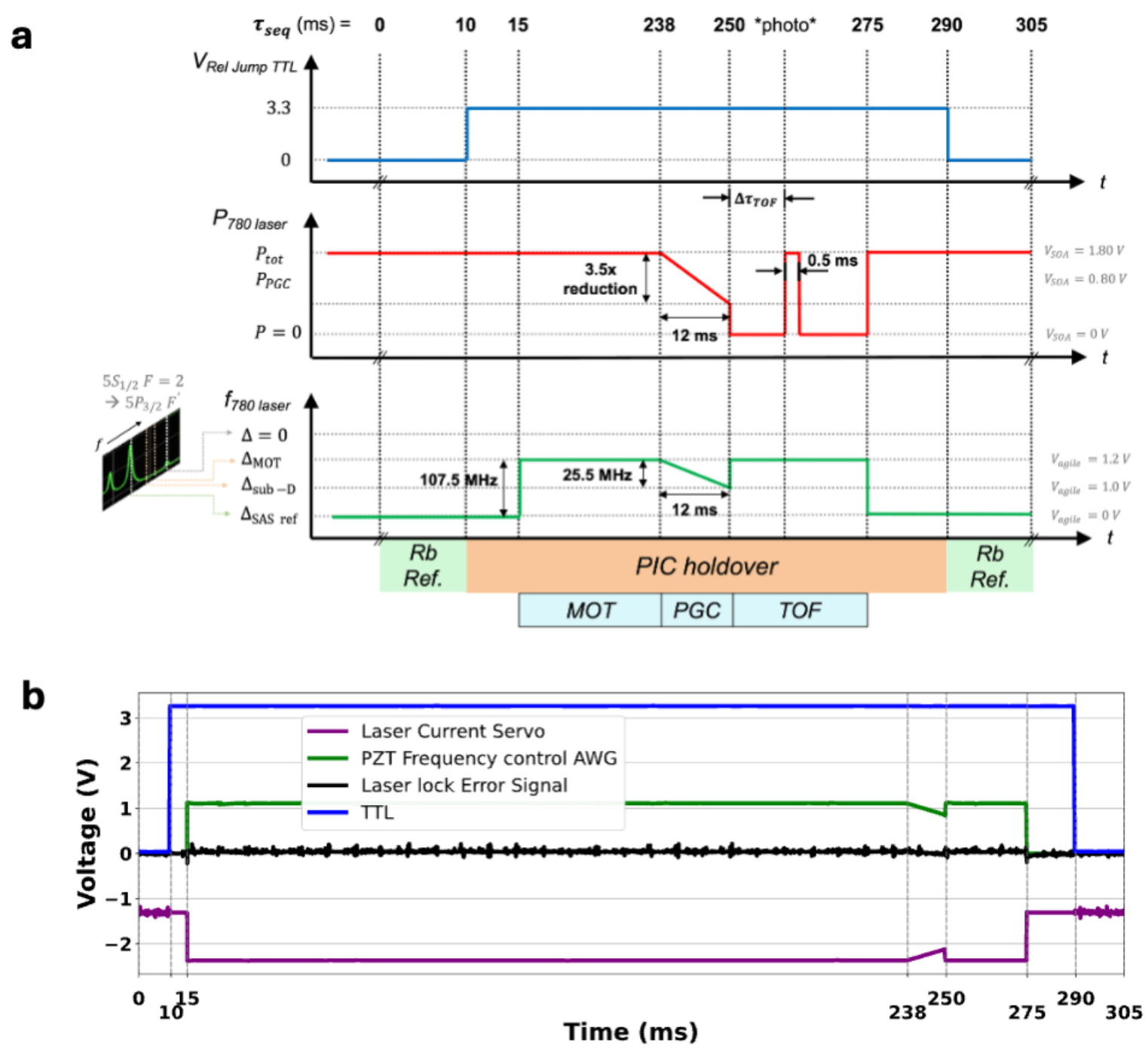

- What extinction ratio do you achieve when the SOA is turned off (P=0)?

<u>Response:</u> As addressed in the previous comment, reproduced again here. We measure an optical on/off ratio (rather than a traditional extinction ratio) of 55 dB (34.7 mW on, 0.100 µW off), where the off-state power includes residual leakage, ASE, and background contributions.

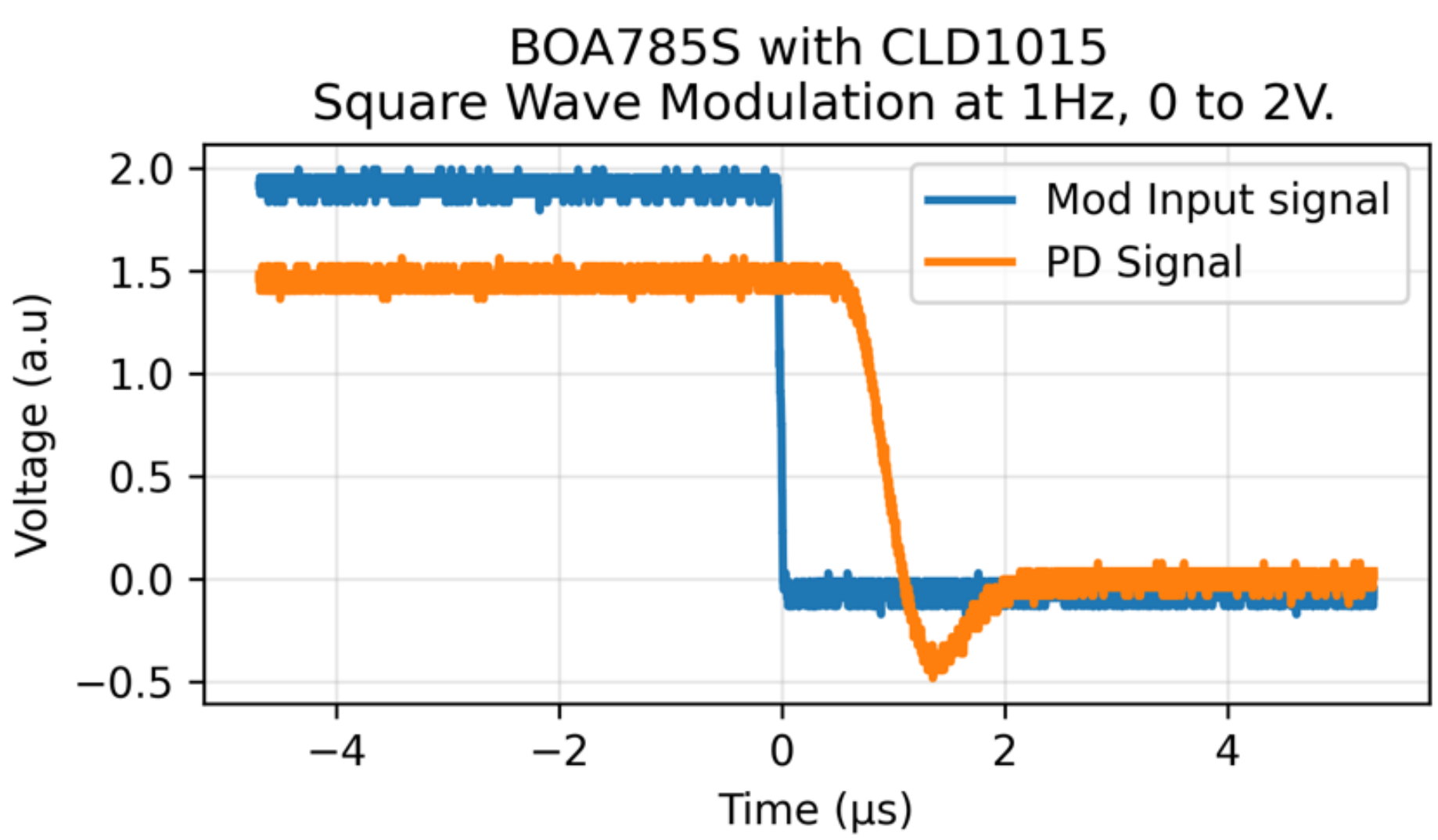

- In Figure S5 [Now labelled Suppl. Fig. 7], can the authors make plots for S5A and S5B have a common x axis so that timing sequences from both plots can be aligned?

Response: The figure has been updated to the figure below (which has now become Supplementary Figure 7). While the plots do not have exactly the same x-axis spacings (the plot (a) was spaced for clarity not to scale), labelling the same time points in (a) and (b) and aligning the start times allows sufficient comparison. Additionally, plot (b) now starts at the same point of the sequence as part (a) which makes the comparison far clearer. Thank you, this has improved the figure.

Revision: Supplementary Information, Supplementary Figure 7

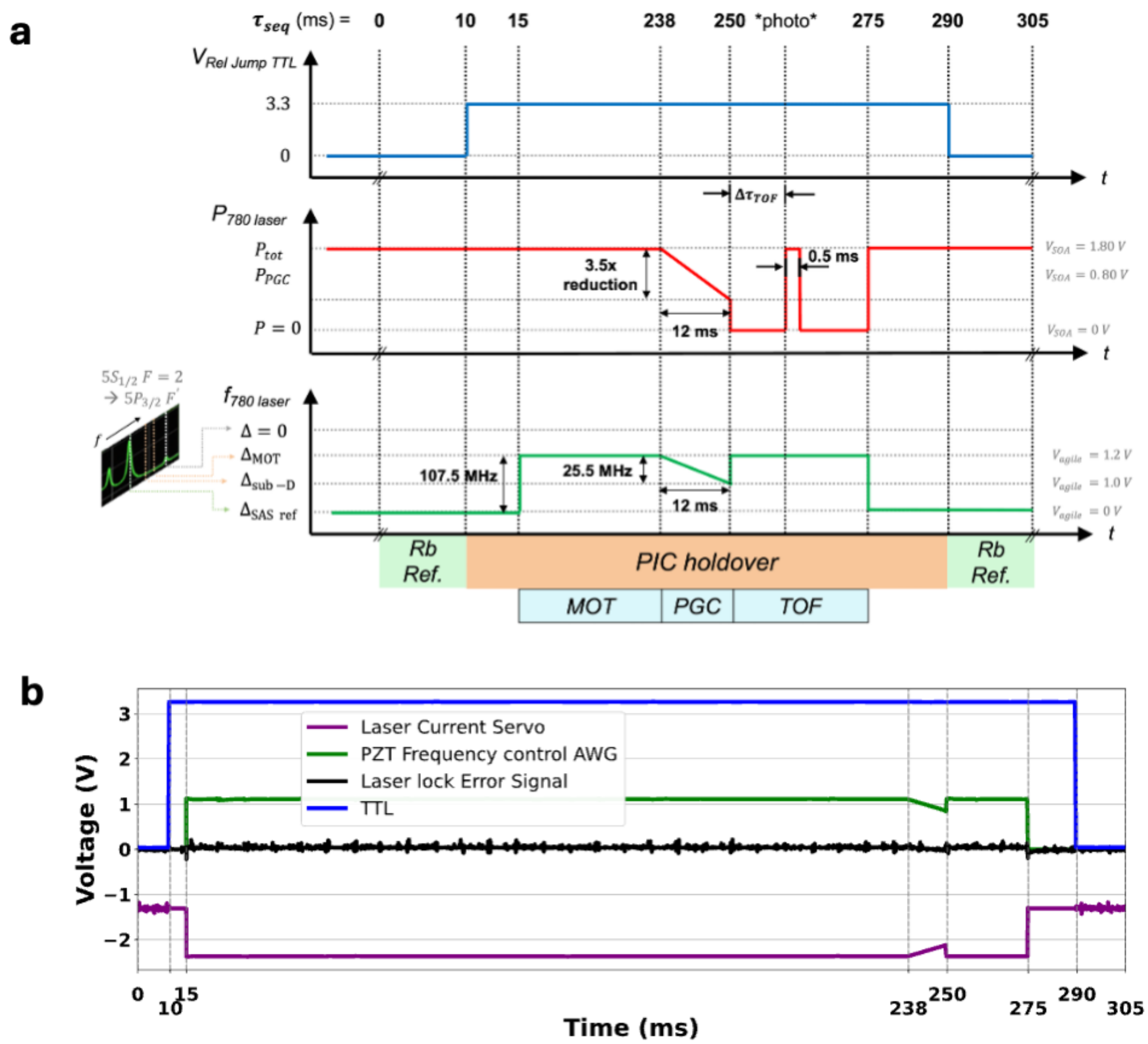

• Figure S6 [Now labelled Suppl. Fig. S8]: PZT Hysteresis and atom number stability.
- To which 87Rb D2 transition is the reference laser locked in these beat-note measurements?
- What zero-point or normalization is used for the beat-note frequency axis?

Response: This should have been included. The reference laser in the beat note is continuously PDH-locked to the ^{87}Rb F=1 → F'=(1,3) transition, which is a little less than 80 MHz redshifted from the cooling SAS reference ^{87}Rb F=1 → F'=(2,3) transition.

Revision: Main text, Figure 5 caption:

"The reference laser is continuously locked to the ^{87}Rb F=1 → F'=(1,3) crossover transition."

- In Fig. S6B [Now labelled Suppl. Fig. 8b], the drift discussed for Fig. 5B on page 8 of the main text is not coherently discussed. If the device was allowed to warm up for several hours of continuous operation, that should be stated in the figure or caption. If so, why were the data for Fig. 5B not recollected or presented once the PZT resonator was fully warmed up?

Response: We agree with the reviewer that our discussion of Figure 5b and Figure 6b in their respective captions (and in the main body) was lacking. This slow (minute-scale) drift brings to light a limitation in our current control system. Future work aims to address this issue (see our response and edits in reply to your Fig. S1 comment). At the time the beat-note data in Figure 5b were acquired, we had not yet identified that the long-term drift plateaued after extended continuous operation. After this behavior was recognized, the same frequency-counter configuration used for the original beat-note measurement was no longer available, so we were unable to repeat the exact Figure 5b measurement under the warmed-up operating condition. We have therefore clarified this limitation in the main text and caption, and we added Supplementary Figure 8b to show that, after extended operation under stable laser and resonator conditions, the PZT-PIC control scheme can maintain a stable atom number. In future systems, this slow drift can be mitigated either by allowing sufficient thermal/electrical settling before operation or, more robustly, through active feedback as described in our response to the Supplementary Figure 1 comment.

Revision: Supplementary information, Supplementary Figure 8b caption

"Continuous 30-minute record of MOT atom number using the PZT cavity for spectroscopy referencing and for PGC, during which the PZT hysteresis was negligible. This data was collected after powering-on the system for over two hours. This warm-up time allows for the stress-optic response of the PZT to better stabilize, though this level stability is not guaranteed on hours-long timescales. Future work will compensate for the time dependence of PZT drive voltage to resonance shift by means of a slow trim loop using MOT fluorescence as an error signal[13]."

Revision: Main text, "Resonator agility measurements":

"Figure 5b shows the laser beat-note recorded over 20 minutes of continuous operation. While we periodically referenced the PZT cavity to the ^{87}Rb SAS (top), the beat-note remained stable near 78 MHz during the ^{87}Rb lock and 185 MHz during MOT formation. We observed a slow drift in MOT fluorescence over long continuous operation (see extended atom number stability data in Supplementary Figure 8a). The data presented in Figure 5b was taken immediately after system power-up when the drift is most significant (although this was not yet known at the time). The drift in long-term response eventually plateaus after approximately an hour of operation."

Additional comments:

1. The Pt tuners are mentioned but their functionality and use in this work is not described. A brief comment and a reference to [23] would be helpful.

Response: The integrated thermal tuners were not used in the experiments reported in this work. However, they are included in the device design because they provide an additional tuning mechanism that complements the PZT actuator and can be used to extend the resonator tuning range. In this work, the PZT actuator was characterized up to 10 V, corresponding to approximately 10 GHz of resonance tuning. For larger or slower frequency adjustments, the resonator temperature can be modified either through the integrated thermal tuner or the TEC setpoint.

The thermal tuner provides an intermediate tuning mechanism, with a response speed faster than the TEC but slower than the PZT actuator. As a result, it is well suited for slow control loops that compensate for long-term drift or PZT hysteresis while reserving the PZT for rapid frequency tuning. Measurements of the thermal actuator on these devices show a tuning efficiency of 87 MHz/mW. Similar thermal tuning approaches were employed in Ref. [23, Isichenko, Optica 2026], where thermally actuated ultra-high-Q resonators were used for large-range frequency control and atomic-referenced laser stabilization. We have added a brief discussion and reference to Ref. [23 Isichenko, Optica 2026] in the revised manuscript.

Revision: Supplementary information, Supplementary Figure 1. Included panels e,f for the thermal tuning measurements for die 6.

"... e) Thermal tuner measurements for die 6, f) demonstrating a tuning strength of 88.4 MHz/mW".

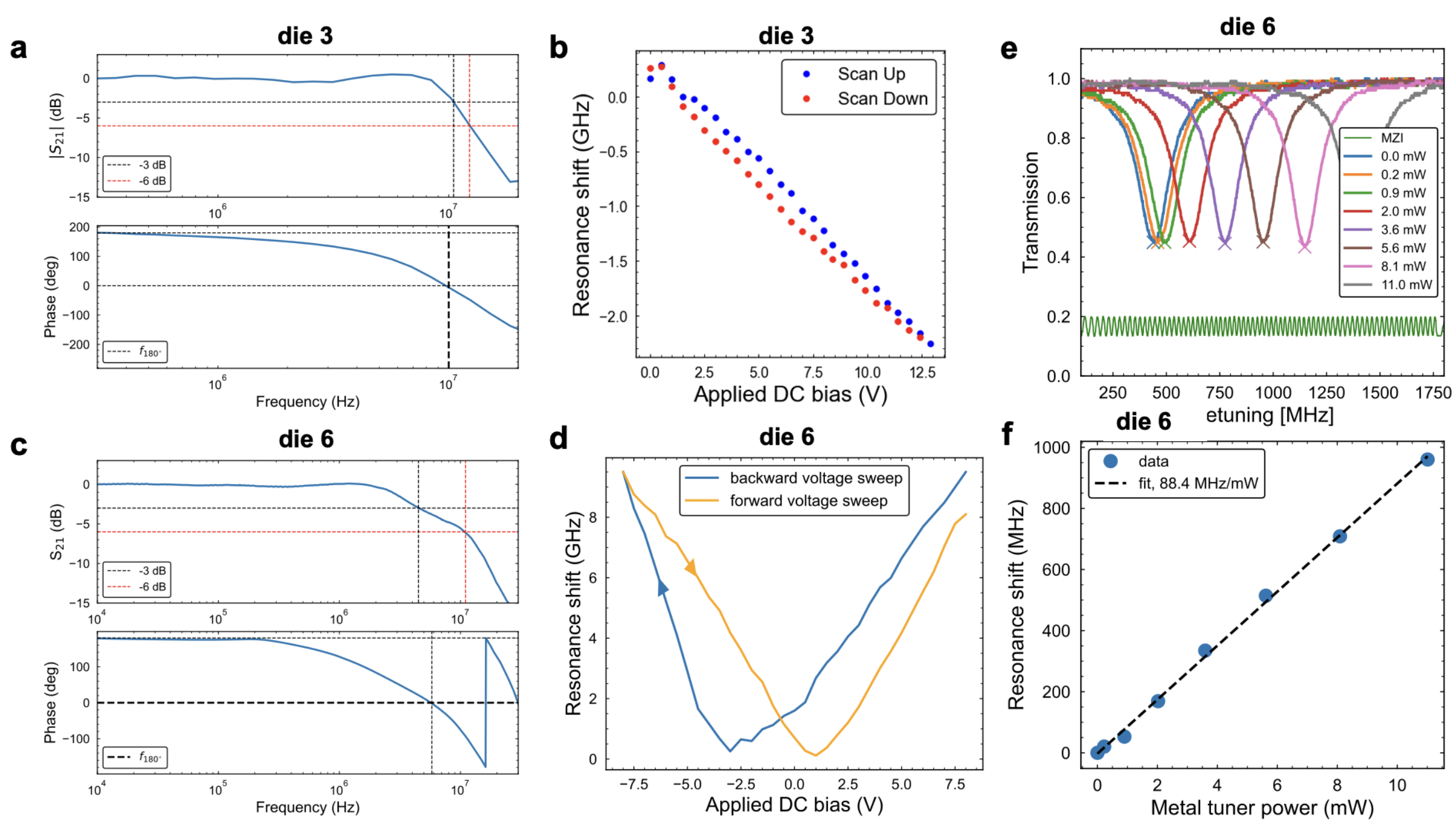

Revision: Supplementary information, "Thermal tuning"

"The resonator includes integrated platinum (Pt) thermal tuners. When used with the resonator, these devices exhibit a thermal tuning efficiency of 88.4 MHz/mW (Supplementary Fig. 1e,f). The

tuners consist of 250 nm thick Pt heaters with a width of 5 and an edge-to-edge separation of 20 from the waveguide. The Pt tuners were not used in the experiments reported here. Instead, frequency control was achieved using the PZT actuator and a thermo-electric cooler (TEC) integrated into the package. Similar Pt thermal tuners were previously employed for large-range frequency control and atomic-referenced laser stabilization in Ref. [6]. In future work, the Pt tuners could serve as a slow trim actuator for compensating long-term frequency drift or PZT hysteresis while reserving the PZT actuator for rapid frequency tuning."

2. The manuscript needs more proof-reading.

- There are many instances of the authors re-defining an acronym already defined earlier in the paper.

  Response: We thank the reviewer for their careful reading of our manuscript. We have gone through the manuscript again and removed duplicate entries such that each acronym is defined once per section including the abstract, the main body, the main figures, the supplementary material, and the supplementary figures.

  Revision: Updated acronyms include MOT, PGC, SAS, SOA, AWG, ESA, SIL, TOF, TRN, ER, FPGA, PDH, DBR, PIC, LO, FSR

- There are several instances of acronyms used that are not defined.

  Response: The reviewer is correct to point this out. We have gone through the paper again and ensured that all abbreviations are clearly defined at the time of first usage in a section. A list of added acronym definitions is given below.

  Revision: "field programmable gate array (FPGA)", "Pound–Drever–Hall (PDH)", "distributed Bragg reflector (DBR)", "photonic integrated circuit (PIC)", "local oscillator (LO)", and "free-spectral range (FSR)".

- There is a parenthesis missing in the bottom paragraph of page S2: "(Fig. S1A. For die 3, the -6dB roll-off is measured at 12.3 MHz".

  Response: Fixed.

  Revision: "For die 6, the -3 dB and -6 dB response roll-offs occur at 4.5 MHz and 11 MHz, respectively, and the 180◦ phase-lag point is observed at 5.8 MHz (Supplementary Fig. 1c)."

- There is a grammatical mistake on page 17: "(d) One period from of (c) overlaid with... ".\

  Response: We have removed the redundant word:

  Revision: "(d) One period of (c) overlaid with 100 consecutive cycles, showing the stability of the PZT response during this period"

- The font size on pages S10, S12, and S13 are different than the rest of the manuscript.

Response: Corrected.

- There are inconsistent references to rubidium and other elements throughout the paper, such as using "Rb", "rubidium", "87Rb", "titatium", "Ti", etc.

Response: We have modified the manuscript and now refer to the element as “rubidium-87” once at the start of the main body and the supplement and later consistently use “87Rb” throughout. Additionally, the usage of “SiN” and writing out “titanate” has been enforced for clarity.

Revision: “rubidium-87” at the start of important blocks, “^{87}Rb” subsequently. Also, “SiN” and “titanate” are used consistently throughout.

- There is inconsistent use of hyphens, both in hyphenated compound words and in reported numbers with units.

Response/Revision: We have gone through the manuscript again and consistently applied hyphenation (or lack thereof) for key phrases like “photonic integrated circuit”, “lead zirconate titanate”, “sub-Doppler”, “crossover”, “780-nm laser”, and “12-ms ramp”. Now, the only time hyphens are not used between a number and its unit are in the case that the number acts as a direct modifier for a noun.

References

1. Nguyen, M. D., Houwman, E. P. & Rijnders, G. Large piezoelectric strain with ultra-low strain hysteresis in highly c-axis oriented Pb(Zr0.52Ti0.48)O3 films with columnar growth on amorphous glass substrates. *Sci. Rep.* **7**, 12915 (2017).
2. Isichenko, A. *et al.* Multi-laser stabilization with an atomic-disciplined photonic integrated resonator. *Opt. Quantum* **4**, 232–240 (2026).
3. GmbH, W. Measurement of voltage noise density with a Red Pitaya. *WiredSense* https://www.wiredsense.com/tutorial/measurement-of-voltage-noise-density-with-a-red-pitaya.
4. lneuhaus. Red Pitaya DAC performance. *Shards of Silicon* https://ln1985blog.wordpress.com/2016/02/07/red-pitaya-dac-performance/ (2016).
5. Wang, W., Subhankar, S. & Britton, J. W. A practical guide to feedback control for Pound–Drever–Hall laser linewidth narrowing. *Appl. Phys. B* **131**, 146 (2025).
6. Montifiore, N. *et al.* Blue to near-IR integrated PZT silicon nitride modulators for quantum and atomic applications. *Opt. Express* **34**, 17295–17303 (2026).
7. Wang, J., Liu, K., Harrington, M. W., Rudy, R. Q. & Blumenthal, D. J. Silicon nitride stress-optic microresonator modulator for optical control applications. *Opt. Express* **30**, 31816–31827 (2022).
8. Chauhan, N. *et al.* Chip scale coil stabilized Brillouin laser driving a room temperature trapped ion qubit. *Nat. Commun.* https://doi.org/10.1038/s41467-026-69948-2 (2026) doi:10.1038/s41467-026-69948-2.
9. Pribošek, J., Diaci, J. & Sinzinger, S. Simple unimorph deformable mirrors fabricated from piezo buzzers. *J. Micromechanics Microengineering* **26**, 055009 (2016).

10. Isichenko, A. *et al.* Photonic integrated beam delivery for a rubidium 3D magneto-optical trap. *Nat. Commun.* **14**, 3080 (2023).
11. Nie, Z., Cui, Y., Huang, J., Wang, Y. & Chen, T. Precision open-loop control of piezoelectric actuator. *J. Intell. Mater. Syst. Struct.* **33**, 1198–1214 (2022).
12. Wang, X., Budinger, V. & Gourinat, Y. A Specific Methodology of Creep Compensation for Piezoelectric Actuators by Open-Loop Control. *Appl. Mech. Mater.* **281**, 141–145 (2013).
13. Devasia, S., Eleftheriou, E. & Moheimani, S. O. R. A Survey of Control Issues in Nanopositioning. *IEEE Trans. Control Syst. Technol.* **15**, 802–823 (2007).
14. Sabarianand, D. V., Karthikeyan, P. & Muthuramalingam, T. A review on control strategies for compensation of hysteresis and creep on piezoelectric actuators based micro systems. *Mech. Syst. Signal Process.* **140**, 106634 (2020).
15. Corato-Zanarella, M. *et al.* Widely tunable and narrow-linewidth chip-scale lasers from near-ultraviolet to near-infrared wavelengths. *Nat. Photonics* 1–8 (2022) doi:10.1038/s41566-022-01120-w.

In this manuscript, the authors demonstrate sub-Doppler cooling of ^{87}Rb atoms in a 3-D magneto-optical trap (MOT) using a sideband-free scheme based on a fully integrated, low-power, fast-tunable optical frequency reference instead of bulk AOMs/EOMs, especially for cooling and detection beams. Their integrated, PZT-tunable Si_3N_4 ring resonator ($Q \simeq 2–3 \times 10^6$) serves both as a short-term frequency reference and as a fast, low-power frequency shifter for a commercial-off-the-shelf (COTS) 780 nm laser. The laser is Pound-Drever-Hall (PDH) locked to the high-Q resonator, whose resonance is itself periodically disciplined to an ^{87}Rb saturation-absorption-spectroscopy (SAS) feature (the F = 2 → F' = 2, 3 crossover) to ensure long-term stability. During each cooling cycle, the SAS serve is temporarily gated off, and programmable voltages on the PZT actuator shift the cavity resonance with low power (≲10 nW)—and hence the locked laser—through the tens-of-MHz detunings needed for MOT loading, followed by a sub-Doppler polarization-gradient-cooling (PGC) ramp (15 MHz over 12 ms), while a COTS semiconductor optical amplifier (SOA) supplies intensity control. In their device characterization, the ring provides ≃10 GHz of total tuning (≈1 GHz/V) with an 11 MHz small-signal bandwidth. Using this sideband-free scheme—in which no bulk AOMs, EOMs, or multi-watt RF drivers are required—the authors cool atomic clouds to 16 μK and confirm atomic temperatures and atom numbers through several methods: PIC-based laser cooling for a steady-state MOT, PIC-based frequency ramp only, PIC-based frequency ramp & SOA intensity control, and conventional AOM-based frequency ramp & SOA intensity control.

This multidisciplinary work between atomic physics and integrated photonics is impressive and represents a significant step toward fully integrated, PIC-based cold-atom systems by eliminating bulky, high-power modulator chains. They showed (1) dual-stage PDH and SAS locking in a single PIC package with cycle-to-cycle repeatability; (2) atom temperature comparison and beat-note stability data, and device characterization, as well as a path shown toward a full PIC driven laser system.

I recommend accept with major revisions for publication in Nature Communications. The authors may need to present several results more quantitatively. In particular, although a companion manuscript (Ref [23], doi:10.48550/arXiv.2509.09124) reports static measurements of frequency stability and linewidth narrowing under quasi-static locking, the dynamic switching performance required for laser-cooling cycles warrants its own quantitative analysis.

General Comments:

1. Please provide an energy level diagram showing the relevant laser transitions for a general audience.

2. Please describe coherently which parts of your laser system are COTS (e.g. repump and SOA lasers) and which are custom PIC-based subcomponents (e.g. the PZT-tuned resonator).

3. To illustrate the Doppler-cooling and sub-Doppler PGC processes,
   - Please explain coherently the cooling and repump beams: (1) the optical transitions of the required lasers and how each laser is locked to its corresponding atomic transition; and (2) the schemes for frequency control and intensity control & stabilization, as well as the optical-switching mechanism (including extinction ratio)

- Please explain the configuration of the atom-detection/fluorescence beam used to measure atom temperature and atom number.

4. Please add error bars on your key data plots (e.g. atom temperature and atom number measurements).

5. Please coherently explain the pro and cons of this sideband-free scheme for next-generation, low-SWaP laser system architectures.
   - This approach may enable multiple beams (for cooling, detection, and state preparation) at different frequencies from the same laser in a time-sequenced cycle.
   - Sequence: first, the laser is locked to the resonator at frequency 1 (Rb discipline); next, the output is shut off via an optical switch, and the laser is tuned to frequency 2 (no Rb discipline) to produce a second beam; then the switch is reopened to resume output; next, the output is shut off again, the laser is tuned to frequency 3 (Rb discipline), and finally, the optical switch is reopened to resume the output.
   - The authors may need to detail (1) the Rb-disciplining procedure—for example, why a 25-ms interval is required and whether it allows sufficient time for frequency scanning and relocking to the atomic transition (SAS locking), (2) the allowable number of Rb-disciplining applications for a given cycle time (from short to long), and (3) any delays or dead times (e.g. in scan and relocking modes) within or between Rb-disciplining applications.
   - Frequency jumps via the PZT-tuned resonator from tens of MHz are acceptable, but larger jumps more than hundreds of MHz can destabilize the lasing condition and cause mode hopping. Please address this issue quantitatively.
   - What is the maximum off-resonance hold time (after disabling the F'=2,3 crossover lock and jumping to F'=3)? Can you extend this hold time by using a small frequency jump or scan range—which perturbs the lasing condition less (for example, by locking to directly to F'=3)?
   - Lasing conditions can be affected by frequency jump and frequency scanning. Does the instantaneous laser linewidth differ in switched and static configurations?

6. Please describe in detail how on-chip power control and optical switching will be implemented in your approach. Specifically:
   - Do you plan to replace the COTS SOA with a fully integrated, PIC-based SOA or with a PZT-tuned resonator for optical power ramping and switching in PGC or other sequences?
   - What switching times and extinction ratios do you expect?

7. Please explain the advantages and limitations of this approach under harsh environmental conditions, such as thermal cycling and vibration. For example:
   - The thermoelectric cooler (TEC) used to stabilize the PZT-tuned resonator can suppress moderate temperature fluctuations, but its performance may be insufficient during large ambient temperature swings or when the thermal mass heats up.
   - A micro resonator is more robust than a bulk cavity, yet it remains susceptible to temperature- and vibration-induced resonance shifts.

8. Please quantify the frequency stability of the 780-nm laser in your approach. While Figs. 5 and S6 qualitatively demonstrate the stability and repeatability of the tuned laser frequency, a more convincing plot would show the switched-frequency, Allan deviation (ADEV) and frequency noise (Hz^2/Hz), both with and without the Rb disciplining. In your discussions, please address:
   - How does PZT hysteresis influence the frequency stability?
   - What are the noise and drift sources that affect short-term and long-term stability?
   - How does stability vary as the interval between disciplining events changes (i.e. with different cycle times)?

9. Please quantify the agility of the PZT-tuned resonator, especially for applications requiring rapid cold atom generation. For example:
   - What is the switching time between two frequencies?
   - Does the switching time depend on the tuning direction (i.e., is there hysteresis)?
   - Does it scale with the frequency difference between the starting and target frequencies?
   - Could you define a "slew rate" for the resonator?

Comments on the Figures:

- **Figure 1**: Working principle of rubidium sub-Doppler cooling using an agile integrated PZT-controlled silicon nitride cavity.
  - Please consider including an energy level diagram showing the relevant laser transitions and clarify which parts of your laser system are COTS and which are custom, PIC-based subcomponents.
- **Figure 2**: PZT PIC resonator design and characterization.
  - What is the mechanism affecting the variation in the tuning strength (MHz/V) among the different dies?
- **Figure 3**: Cold atom experiment diagram.
  - You currently lock the cavity (and thus the laser) to the F=2-> F'=2,3 crossover in your SAS cell, then apply a PZT-voltage jump to reach the true F=2 -> F'=3 cooling line.
  - Have you tried instead to lock to the F=2 -> F'=3 peak? That would avoid the per-cycle frequency jump and may lead to better long-term stability in terms of PZT cavity control.
- **Figure 4**: MOT temperature measurements for different cooling laser control configurations.
  - Do you use the same parameters on cooling, repump, atom-detection/fluorescence beams and TOF measurement protocols for (I) No PGC, (II) PZT Freq ramp only, (III) PZT Freq ramp & SOA power ramp, and (IV) AOM reference?
  - Are the horizontal and vertical atomic temperatures the same?
  - Please include error bars on the data points.
- **Figure 5**: Cooling laser frequency agility measurements.
  - The changing atom number/fluorescence can be attributed to various sources. How can you know that the change in atom signal is primarily due to frequency changes? For example, are the laser intensities also stabilized? How does the frequency stabilities compare during the MOT vs probing stages?
  - Figure 5 remains qualitative. On page 8 (second paragraph), the authors describe a slow drift in MOT fluorescence over long continuous operation, which they attribute to

frequency-jump errors when the PZT resonator moves from the “disciplined” voltage to the MOT-detuning voltage. However, no numerical value for this draft appears in Figure 5 or the main text.

- In Supplementary Materials (regarding Figure S6), the authors mentioned “Without Rb-referencing, the cooling laser exhibits a ~50 MHz drift over 20 minutes, whereas with Rb-referencing, the drifts are reduced to the single-MHZ range over the same interval.” With the SAS servo disabled, but that measurement does not directly characterize the short-term drift or hysteresis over a single 235 ms MOT + PGC cycle. The manuscript specifies a 223 ms MOT loading time followed by a 12 ms PGC ramp, implying that without re-referencing every 235 ms the cavity frequency would wander beyond the trap detuning.
- Please clarify whether the PZT resonator’s drift during one cooling cycle imposes such a limit and distinguish this slow drift from the separate issue of PZT hysteresis and frequency-jump accuracy.
- Is the lower plot in figure 5B continuously holding the MOT, or is this also cycling the MOT load/detection cycle while just not re-referencing to the SAS signal in between?

- **Figure 6**: Example integrated cold atom system enabled by the 780 PZT resonators.
  - Fig. 6 shows the detailed vision for a fully integrated cold-atom instrument.
  - Do you use a standard 3D MOT for this experiment, or a MOT with grating couplers as shown in Fig. 6?
- **Figure S1**: PZT device characterization.
  - How did you address PZT hysteresis in the experiment?
  - Figure S1A shows an S21 measurement for die 6, but then figure S1B shows a hysteresis curve for die 3. Why are different characterizations being presented for different devices? Is data being cherry picked here, or was there some other reason for showing different data for different devices?
- **Figure S2**: PZT PIC packaging.
  - Could you explain the detailed optical power levels at each stage used for the PDH locking and the Rb SAS locking—accounting for all insertion losses, fiber-coupling losses, and fiber-to-waveguide coupling losses? Please start from the nominal output power of the 780 nm COTS laser (e.g., 50-100 mW). Why the propagation loss (20 dB/m) in this paper is higher than another reference [30] (0.44 dB/m)?
- **Figure S3**: Laser stabilization.
  - You state that a 1.2 MHz direct-laser-current modulation is used to generate the error signals for both the PDH lock and the Rb SAS locks. In Rb-disciplining mode, you may need to scan and relock to the F=2 → F’ = 2,3 crossover line. How much additional time do you need for this procedure? Do you leave on the laser output on Rb atoms during this scanning and relocking modes? (also, related to Fig. S5)
- **Figure S4**: MOT system configurations for temperature measurements.
  - Do you use the same parameters of cooling, repump, atom-detection/fluorescence beams and TOF protocols for (I) No PGC, (II) PZT Freq ramp only, (III) PZT Freq ramp & SOA power ramp, and (IV) AOM Freq ramp & SOA power ramp?
- **Figure S5**: Experiment timing.
  - In Table S1, the symbol “Δ” is given as 25.5 MHz. Does this correspond to the MOT detuning (Δ_{MOT}) labeled in Figure S5? Please use the same notation in both the table and the figure.

- In Figure S5, the SAS-reference detuning $\Delta_{SAS\ ref}$ is 133 MHz (the frequency difference between the F'=2,3 crossover to the F'=3 line). Subtracting Δ_{MOT} (25.5 MHz) gives 107.5 MHz, yet the timing diagram labels this step as 118 MHz. Can you explain that discrepancy?
- What extinction ratio do you achieve when the SOA is turned off (P=0)?
- In Figure S5, can the authors make plots for S5A and S5B have a common x axis so that timing sequences from both plots can be aligned?

- **Figure S6**: PZT Hysteresis and atom number stability.
  - To which ^{87}Rb D_2 transition is the reference laser locked in these beat-note measurements?
  - What zero-point or normalization is used for the bean-note frequency axis?
  - In Fig. S6B, the drift discussed for Fig. 5B on page 8 of the main text is not coherently discussed. If the device was allowed to warm up for several hours of continuous operation, that should be stated in the figure or caption. If so, why were the data for Fig. 5B not recollected or presented once the PZT resonator was fully warmed up?

Additional comments:

1. The Pt tuners are mentioned but their functionality and use in this work is not described. A brief comment and a reference to [23] would be helpful.
2. The manuscript needs more proof-reading.
   - There are many instances of the authors re-defining an acronym already defined earlier in the paper.
   - There are several instances of acronyms used that are not defined.
   - There is a parenthesis missing in the bottom paragraph of page S2: "(Fig. S1A. For die 3, the -6dB roll-off is measured at 12.3 MHz".
   - There is a grammatical mistake on page 17: "(d) One period from of (c) overlaid with... ".
   - The font size on pages S10, S12, and S13 are different than the rest of the manuscript.
   - There are inconsistent references to rubidium and other elements throughout the paper, such as using "Rb", "rubidium", "^{87}Rb", "titatium", "Ti", etc.
   - There is inconsistent use of hyphens, both in hyphenated compound words and in reported numbers with units.
